# Biopsychosocial and Occupational Health of Emergency Healthcare Professionals: A Systematic Review and Meta-Analysis

**DOI:** 10.3390/nursrep15120430

**Published:** 2025-12-04

**Authors:** Rafael Galindo-Herrera, Manuel Pabón-Carrasco, Rocío Romero-Castillo, Miguel Garrido-Bueno

**Affiliations:** 1 Department of Traumatology Emergencies, Virgen del Rocío Hospital, 41013 Seville, Spain; rafgalher@alum.us.es; 2 Research Group PAIDI-CTS-1050, “Complex Care, Chronicity and Health Outcomes”, Department of Nursing, Faculty of Nursing, Physiotherapy and Podiatry, University of Seville, 41009 Seville, Spain

**Keywords:** emergency healthcare professionals, burnout, workplace violence, job satisfaction, stress, depression, resilience, turnover intention

## Abstract

**Background/Objectives:** Emergency healthcare professionals are continually exposed to high clinical and organizational demands that compromise their mental, physical, and occupational health. This systematic review and meta-analysis examined the prevalence and interrelations of biopsychosocial and work-related health outcomes among emergency personnel, providing an integrated synthesis of recent empirical evidence. **Methods:** A systematic search of PubMed, Scopus, Web of Science, and CINAHL identified 6214 records, of which 50 studies met inclusion criteria and were analyzed (total *n* = 278,000 emergency professionals). Eligible studies (2020–2025) evaluated biopsychosocial outcomes (burnout, depression, stress, resilience, sleep quality) and occupational indicators (workplace violence, job satisfaction, effort-reward imbalance, engagement, turnover intention). Meta-analyses were conducted using random-effects models (DerSimonian-Laird method), producing pooled prevalence estimates for each outcome based on the number of studies that reported the corresponding variable. Risk of bias was assessed using the Joanna Briggs Institute tools, with most studies rated as moderate-to-high quality. **Results:** Pooled estimates showed fair self-perceived health in 44.0%, severe burnout in 10.7%, depressive symptoms in 35.1%, moderate-to-severe stress in 74.6%, and poor sleep quality in 40.1% of staff. Workplace violence affected 76.9% of professionals. Job satisfaction averaged 68.1%, turnover intention 62.1%, and effort-reward imbalance 61.9%. Resilience was predominantly moderate (33.9%). Considerable heterogeneity was observed; however, patterns were consistent across regions and professional roles. **Conclusions:** Emergency healthcare personnel face substantial biopsychosocial strain and occupational risks, driven by persistent structural pressures. Health systems should implement integrated organizational strategies to reduce violence, enhance psychological support, ensure safe staffing, and protect rest and recovery. Improving staff well-being is essential for maintaining a resilient and effective emergency care workforce.

## 1. Introduction

The World Health Organization defined health as a state of complete physical, mental, and social well-being [1]. Later contributions described health as the ability to adapt and manage oneself when facing physical, emotional, or social challenges [2].

Work or occupational health expands on this view by recognizing the direct impact of working conditions on professional well-being. In the healthcare sector, occupational health involves more than the absence of illness: it includes the presence of conditions that allow workers to remain physically and psychologically stable while fulfilling their roles [3]. Hospital emergency departments represent one of the most demanding environments within healthcare systems. Constant exposure to uncertainty, critical cases, time pressure, and emotional demands defines the daily reality of these services [4]. This demands constant physical presence and emotional preparedness. As a result, professionals accumulate stress over time. Without appropriate structural support, this situation increases the likelihood of long-term health deterioration.

Emergency workers often report physical symptoms such as fatigue, musculoskeletal disorders, sleep disturbances, and gastrointestinal problems [5]. Psychological difficulties are also frequent and include higher anxiety, work stress, lower resilience, and symptoms associated with burnout [6]. Resilience refers to the capacity of an individual to recover from adverse events and to maintain emotional stability under pressure [7]. Burnout is a psychological condition that arises in response to prolonged work-related stress. It involves three core components: emotional exhaustion reflects the feeling of being emotionally drained, depersonalization refers to a distant or indifferent attitude toward patients or colleagues, and a reduced sense of personal accomplishment describes a perception of inefficacy or failure in professional duties [8]. These experiences may negatively affect personal well-being, increase the risk of clinical errors, and reduce the quality of patient care [9].

Several organizational factors intensify these health risks. These include long and irregular shifts, insufficient rest periods, limited autonomy in decision-making, poor communication, lack of recognition, and exposure to verbal or physical violence. Many professionals also face contract instability and high turnover rates [10]. In many healthcare systems, these problems remain under-recognized. They are sometimes framed as an inevitable part of a caring profession rather than as indicators of organizational dysfunction [10,11].

The diversity and frequency of these problems [11] suggest a need to reach a more consistent understanding of their actual prevalence. However, the available literature is fragmented on which biopsychosocial and work health problems and protective factors are most widespread among emergency healthcare professionals, and few studies combine these factors into a unified model. A more comprehensive and integrated perspective may help promote healthy, safe, and sustainable work environments in hospital emergency services.

Therefore, this study sought to answer the following question, structured using the PEO framework: Among emergency healthcare professionals (P), what biopsychosocial and occupational exposures, including both risk and protective factors (E), are associated with their physical, psychological, and organizational well-being, and what is their prevalence and distribution (O)?

## 2. Materials and Methods

This was a systematic review with meta-analysis about the biopsychosocial and work health of emergency workers. It was carried out by four researchers (two of whom hold a PhD and two hold an MSc) in two subsequent phases: the systematic review and the meta-analysis phase.

The systematic review phase was conducted first to summarize the most recent evidence about the research topic. It followed the Cochrane Handbook for Systematic Reviews of Interventions, the PRISMA 2020 and AMSTAR-2 guidelines, and Joanna Briggs Institute (JBI) Checklist for Systematic Reviews and Research Syntheses to ensure a high methodological quality [12,13,14,15]. A review protocol was registered in PROSPERO (ID: CRD42025645767). The meta-analysis phase was conducted to provide further insights into the combined evidence of the findings.

### 2.1. Eligibility Criteria

A preliminary search was conducted based on a PEO framework to help establish eligibility criteria [16]. Included studies in the systematic review phase were conducted in adult hospital emergency settings (e.g., adult emergency departments, emergency intensive care units, and/or emergency surgery units), addressed the biopsychosocial and work health of emergency workers; were published in the last five years (2020–2025), did not follow a review, protocol, pilot or questionnaire validation study design; were not congress communications; did not appear as retracted in the Retraction Watch database; and did not include confounding information. Confounding information are factors that can distort the relationship between an intervention/exposure and an outcome [17]. In this review, confounding information was the assessment of work-related outcomes without any connection to the biopsychosocial health of emergency workers. No language restrictions were applied. Included studies in the meta-analysis phase reported the biopsychosocial or work health outcomes of emergency workers in a proportional manner (i.e., ratios, frequencies, prevalences, and/or proportions).

### 2.2. Search Strategy

A search strategy based on a PEO framework was developed with the Medical Subject Headings (MeSH) thesaurus-controlled vocabulary. Descriptors were combined with Boolean operators, truncations, quotation marks, and parentheses. General (PubMed, Scopus, Embase, and Web of Science) and nursing and psychology-specific (CINAHL, APA PsycInfo, and APA PsycArticles) databases were consulted between January and September 2025. No searches were conducted in any other additional resources.

The search strategy was as follows: (“Occupational health” OR “Workplace health” OR “Job stress” OR “Workplace safety” OR “Work environment” OR “Occupational risks” OR “Occupational diseases” OR “Health workforce”) AND (“Traumatology” OR “Trauma care” OR “Emergency departments” OR “Emergency services” OR “Urgent care” OR “Emergency Medical Services” OR “Emergency Nursing” OR “Emergency Service, Hospital”) AND (“Physical health” OR “Mental health” OR “Psychological stress” OR “Work-related stress” OR “Burnout” OR “Musculoskeletal disorders” OR “Occupational injuries” OR “Fatigue” OR “Resilience” OR “labor abandonment” OR Absenteeism OR “Change of service” OR “Intention to leave” OR “Personnel turnover”) AND (“Interventions” OR “Workplace interventions” OR “Well-being programs” OR “Stress management” OR “Preventive strategies” OR “Occupational safety interventions”) AND (Nurs* OR “Trauma nursing” OR “Emergency nurs*”) NOT (“surg*” OR “child*” OR “protocol*” OR “review*”). The search strategy was the same across all databases except for Scopus, in which the Boolean operator NOT was changed to AND NOT due to its technical requirements (Table S1, Supplementary Material).

### 2.3. Study Selection

Study selection was based on the previously mentioned eligibility criteria and was structured in three stages: pre-screening (in which duplicated studies were sorted out); title and abstract screening; and full text screening. It was independently carried out by two peer reviewers with Zotero v7.0.5 without any automation and/or artificial intelligence tools, and it was graphically represented in a PRISMA 2020 flowchart [13].

Inter-rater reliability was assessed with Cohen’s Kappa coefficient, calculated using the standard formula κ = [(Po − Pe)/(1 − Pe)] where Po represents the observed agreement and Pe represents the agreement expected by chance [18]. The inter-rater agreement rate was Kappa = 0.92. In case of disagreement, consensus was reached through consultation with a third reviewer.

### 2.4. Data Collection and Extraction

Data collection was independently carried out by two peer reviewers with Microsoft Excel version 2403^®^ (R.G.-H and M.G.-B). The inter-rater agreement rate was Kappa = 0.92. In case of disagreement, consensus was reached through consultation with a third reviewer (M.P.-C).

Data extracted for each study were: (a) authorship, publication year and region, (b) study design, (c) sample size, (d) JBI study methodological quality assessment [15], (e) study aim, (f) evaluation instruments, (g) mean, mode, and frequency of participants’ sociodemographic characteristics (e.g., age, gender, sex, and marital status), (h) mean, mode, and frequency of participants work-related outcomes (e.g., shift conditions, perceived workplace violence, effort-reward imbalance, job satisfaction, and turnover intention), and (i) mean, mode, and frequency of participants’ biopsychosocial health-related outcomes (e.g., self-perceived health, work stress, burnout, depression, resilience, and sleep quality).

The included studies’ quality assessment was independently carried out by two peer reviewers with the JBI critical appraisal tools for analytical cross-sectional studies, randomized controlled trials, and quasi-experimental studies [15].

### 2.5. Data Meta-Analysis and Synthesis

Data meta-analysis was simultaneously carried out by two reviewers using a proportion meta-analysis under a random-effects model and the DerSimonian-Laird method. In this context, a proportion meta-analysis plot (random effects) displays the pooled prevalence estimate derived from individual study proportions while accounting for both within-study and between-study variance [19]. Proportions were transformed using the Miller method based on the exact inverse Freeman–Tukey double arcsine transformation [20].

Between-study heterogeneity was assessed through the I^2^ index and Cochran’s Q statistic, while the between-study variance was estimated using a moment-based approach. To assess publication bias, both Egger’s regression test and, when applicable, the Harbord test for bias were performed [21].

The interval ranges used in the meta-analysis followed the scoring criteria defined by each validated scale applied in the included studies. Accordingly, the cut-off points and interpretive categories corresponded to those originally established for each instrument, ensuring conceptual alignment and methodological consistency across the pooled data.

A narrative synthesis of findings was carried out using a conceptual domain-based grouping method. These domains were inductive and simultaneously developed by two reviewers based on recurring patterns in the included studies. These were as follows: (1) characteristics of included studies (in which JBI scores were integrated [15]); (2) characteristics of participants; (3) biopsychosocial health of emergency workers; and (4) work health of emergency workers. These were also presented in a summary of findings table (Table S2, Supplementary Material).

## 3. Results

### 3.1. Characteristics of Included Studies

**Figure 1:**
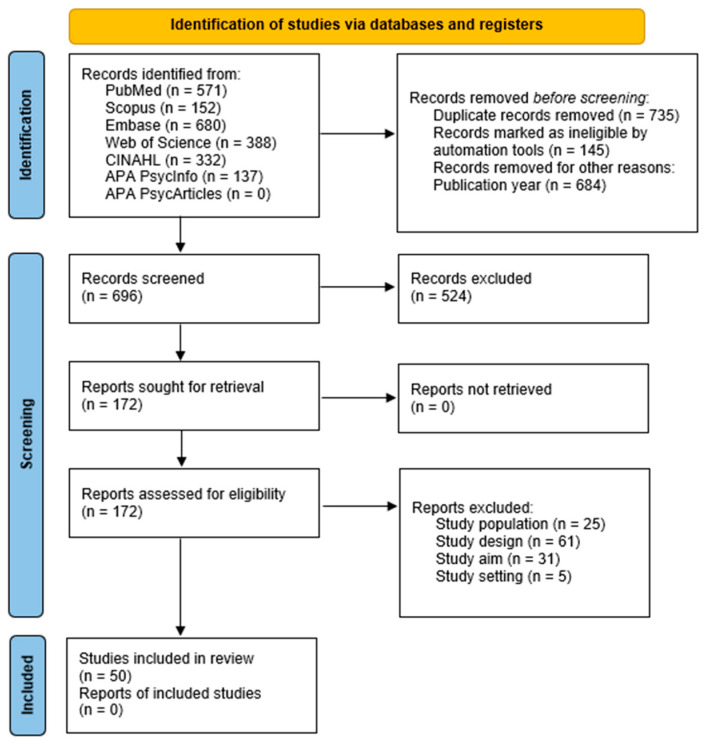
Study selection process PRISMA flow diagram.

A total of 50 studies met the inclusion criteria and were incorporated into the review, as detailed in the PRISMA 2020 flow diagram (Figure 1). They were cross-sectional descriptive (*n* = 47, 94%), longitudinal quasi-experimental (*n* = 2, 4%), and randomized controlled (*n* = 1, 2%) studies. They were published in 2024 (*n* = 12, 24%), 2025 (*n* = 11, 22%), 2023, 2022 (both *n* = 9, 18%), 2021 (*n* = 5, 10%), and 2020 (*n* = 4, 8%). They were conducted in China (*n* = 24, 48%), United States of America (*n* = 7, 14%), South Korea, Spain (both *n* = 3, 6%), Iran, Saudi Arabia (both *n* = 2, 4%), Brazil; Barbados, The Bahamas, Trinidad and Tobago; Germany, India, Italy, Poland, Serbia, Switzerland, and Turkey (all *n* = 1, 2%).

Methodological quality assessment of included cross-sectional descriptive studies revealed that most of them (*n* = 23, 48.9%) scored 8/8 items, that the study that scored the least (3/8 items) was Hancerlioglu et al. [22], and that they scored a mean of 6.9/8 items. Items that were most frequently not followed were item 5 (*n* = 19, 40.4%) and item 6 (*n* = 18, 38.3%). Regarding the quality of longitudinal quasi-experimental studies, the study that scored the most was Dastur et al. [23] (7/9 items), the one that scored the least was Hines-Stellisch et al. [24] (5/9 items), and they scored a mean of 6/9 items. Items that were most frequently not followed were item 2 (*n* = 1, 50%) and item 8 (*n* = 2, 100%). The quality assessment of the only randomized controlled trial included showed that it scored 5/13 items and that the items that were not followed were items 4–6 and 10–13. Scores for all individual studies were presented (Table S2, Supplementary Material).

The study with the largest sample was Jiang et al. [25] (*n* = 111,577), the one with the smallest was Hines-Stellisch et al. [24] (*n* = 10), and their mean sample was *n* = 5589 people.

### 3.2. Characteristics of Participants

All studies reported their participants’ characteristics, except for Dastur et al. [23]. They were male (*n* = 74,846, 42.3%) and female (*n* = 102,206, 57.7%) from *n* = 46 (92%) studies that reported the gender or sex of their sample (Table S2, Supplementary Material).

The sample was a maximum of 61–70 years [26], a minimum of 18 [27], and a mean of 33.40 years old. Twelve (24%) studies also presented their participants’ age with intervals: *n* = 13,256 (28.48%) were ≤30 years, *n* = 20,131 (43.25%) were 30–40 years, *n* = 9579 (20.58%) were 40–50 years, and *n* = 3579 (7.69%) were ≥50 years. Percentages used the denominator *n* = 46,545 participants from the studies whose categories could be mapped. Nine (18%) studies used incompatible age interval cut-offs: Chen et al. [28] (≤ 31: *n* = 4089; >31–≤ 37: *n* = 4117; >37–≤ 43: *n* = 3291; >43: *n* = 3746), Fei et al. [29] (< 25: *n* = 3493; 25–34: *n* = 10,540; >34: *n* = 3549), Kim et al. [30] (<30: *n* = 40; 30–34: *n* = 59; ≥35: *n* = 31), Choi et al. [31] (≤25: *n* = 24; 26–30: *n* = 55; 31–35: *n* = 32; ≥36: *n* = 20), Huang et al. [32] (≤25: *n* = 122; 26–30: *n* = 120; 31–35: *n* = 145; 36–40: *n* = 48; >40: *n* = 31), Ma et al. [33] (<30: *n* = 257; 30–39: *n* = 201; >39: *n* = 64), Hao et al. [34] (≤30: *n* = 141; 31–40: *n* = 136; >40: *n* = 28), Ramanjaneyulu et al. [35] (<30: *n* = 18; 30–45: *n* = 41; >45: *n* = 14), and Jiang et al. [25] (≤30: *n* = 2600; 31–40: *n* = 4977; ≥41: *n* = 2880).

They were single or unmarried (*n* = 25,291, 25.2%), had a partner or were married (*n* = 74,044, 73.9%), or were separated or divorced or widowed (*n* = 854, 0.9%). Percentages used the denominator *n* = 100,189 participants from *n* = 31 (62%) studies whose categories could be mapped.

The sample had a minimum of 31.80 years [36], a maximum of 69.25 years [37], and a mean of 17.65 years of professional experience. Fourteen (28%) studies also presented it with intervals: *n* = 3977 (63.18%) had ≤10 years, *n* = 1782 (28.31%) had 11–20 years, and *n* = 536 (8.51%) had >20 years of professional experience. Percentages used the denominator *n* = 6295 participants from the studies whose categories could be mapped.

They had a minimum of 3.53 years [38], a maximum of 10.70 years [39], and a mean of 5.80 years of experience in emergency departments. Thirteen (26%) studies also presented it with intervals: *n* = 4064 (6.41%) were ≤2 years, *n* = 13,046 (20.59%) were 3–5 years, *n* = 38,083 (60.10%) were 6–10 years, *n* = 8061 (12.72%) were 11–20 years, and *n* = 113 (0.18%) were >20 years. Percentages used the denominator *n* = 63,367 participants from the studies whose categories could be mapped.

Among the eighteen (36%) studies that reported the primary shift worked, *n* = 1937 (26.6%) people worked day morning and afternoon (*n* = 1887, 10.2%), night (*n* = 16,494, 89.4%), 12 h day/evening (*n* = 50, 0.3%), or 12 h night shifts (*n* = 18, 0.1%). Percentages used the denominator *n* = 18,449 participants from the studies whose categories could be mapped.

They worked a minimum of 40.29 h [40], a maximum of 54.80 h [37], and a mean of 47.55 h per week. Five (10%) studies also presented the hours worked per week with intervals: *n* = 2019 (38.46%) worked ≤40 h/week, *n* = 2971 (56.60%) worked 41–58 h/week, and *n* = 259 (4.93%) worked ≥59 h/week. Percentages used the denominator *n* = 5249 participants from the studies whose categories could be mapped. Xie et al. [41] reported its sample daily working instead: *n* = 268 (26.4%) worked 8 h/day, and *n* = 746 (73.6%) worked >8 h/day.

### 3.3. Biopsychosocial Health of Emergency Workers

Biopsychosocial health of emergency workers was assessed through self-perceived health, burnout, depression, stress, resilience, and sleep quality outcomes in included studies.

#### 3.3.1. Self-Perceived Health of Emergency Workers

Global self-perceived health among emergency healthcare professionals clustered predominantly around the midrange, with “fair” or “general” health being the most frequently reported category. A pooled meta-analysis of ten studies estimated the prevalence of fair self-perceived health at 44.0% (95% CI 0.41–0.47), while good health was reported by 30.7% (95% CI 0.25–0.37) and poor health by 16.3% (95% CI 0.12–0.21). Heterogeneity was very high across all categories (I^2^ > 98%, *p* < 0.001), reflecting broad contextual and methodological variability among studies.

Publication bias was assessed using Egger’s or Harbord’s tests, depending on the sample distribution, with no significant bias detected (*p* > 0.05).

**Figure 2:**
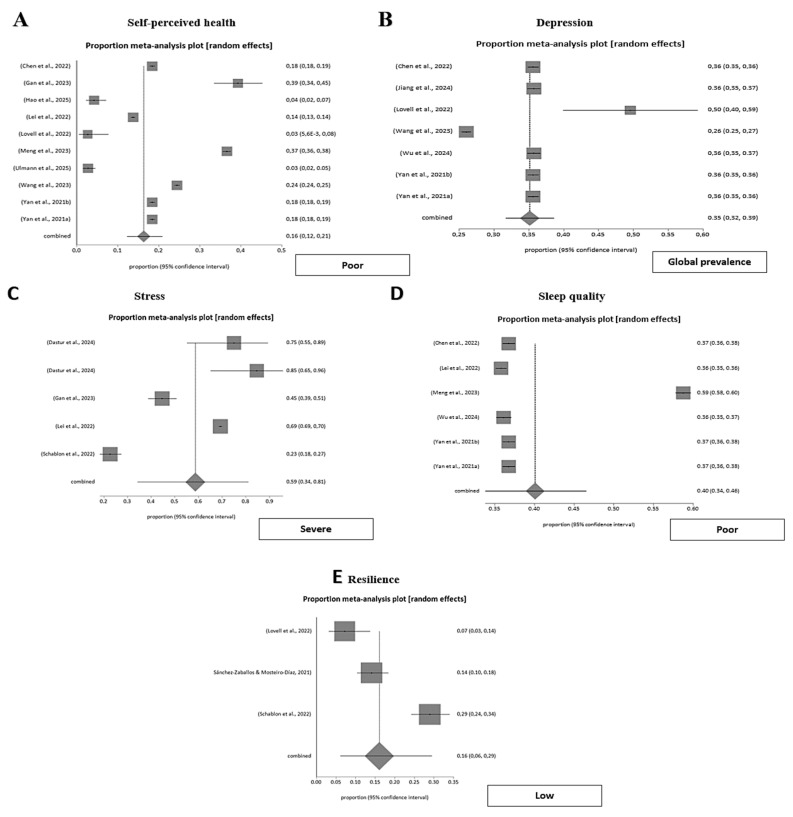

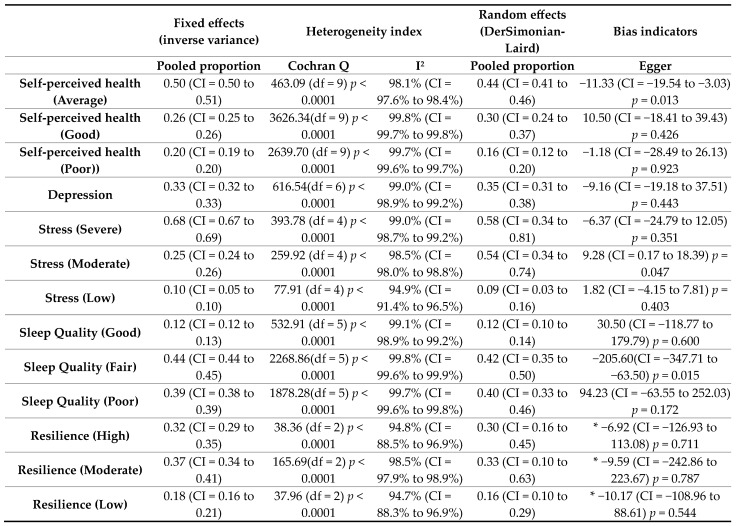
Prevalence of biopsychosocial health levels among emergency healthcare workers. * = Harbord: bias. (**A**) Self-Perceived Health Forest Plot; (**B**) Depression Forest Plot; (**C**) Stress Forest Plot; (**D**) Sleep Quality Forest Plot; (**E**) Resilience Forest Plot [23,25,27,28,34,39,42,43,44,45,46,47,48,49,50].

Overall, these findings confirm that most emergency professionals perceive their health as fair rather than good, consistent with the demanding nature of emergency care. This pattern suggests an underlying imbalance between physical well-being, psychological strain, and occupational stress, highlighting the cumulative impact of workload intensity and shift-related fatigue on perceived health (Figure 2).

For instance, 50.7% reported fair health in Chen et al. [28] (good 30.9%, poor 18.4%), Yan et al. [42,43] (good 30.88%, fair 50.71%, poor 18.42%), and Lei et al. [27] (fair 50.7%, good 35.6%, bad 13.8%).

Additionally, the cohort in Ulmann et al. [39] showed better self-ratings (very good/good 80.5%, moderate 16.5%, bad/very bad 2.8%), while a sample in Hao et al. [34] reported good (58.0%), moderate (37.7%), and poor (4.3%). Some studies followed these distributions: Lovell et al. [44] reported excellent (21.4%), very good (32.1%), good (31.3%), fair (10.7%), and poor (2.7%); and Hao et al. [34] provided complementary self-ratings (good 58.0%, moderate 37.7%, poor 4.3%). However, in Meng et al. [45], poorer global health was frequent (good 14.3%, fair 49.1%, bad 36.6%), and lower self-perceived scores were observed in Gan et al. [46] (poor 38.75 ± 11.62, average 34.65 ± 10.25, good 33.49 ± 10.82, F = 5.517, *p* = 0.004).

Beyond global ratings, studies provided different dimensions of self-perceived health. Ulmann et al. [39] documented frequent physical complaints, such as back/low-back pain at least “a little” in 73.2%, general weakness/fatigue “a little” in 57.7%, sleep-onset/maintenance problems “a little” in 49.4%, and shoulder/neck/arm pain “a little” in 44.2%. They also found that 58.2% had received treatment for physical complaints. Mental-health issues were also common, with “little interest/pleasure” occurring several days in 57.7%, depressed mood/hopelessness several days in 39.0%, sleep problems several days in 48.8%, and fatigue/low energy several days in 56.2%. Rasmus et al. [51] found most participants rated physical health at the top of a 1–5 scale (predominantly 4–5), and Wang et al. [47] observed good (22.5%), fair (53.0%), and bad (24.4%) physical health.

##### Factors Related to Self-Perceived Health of Emergency Workers

These involved sociodemographic characteristics, mental health, and work-related factors. Regarding sociodemographic characteristics, Rasmus et al. [51] found that men reported a higher self-rated mental state than women (*p* = 0.020), while self-rated physical health did not differ by sex (*p* = 0.250).

Poorer self-perceived health also corresponded to worse burnout profiles. Yan et al. [43] and Chen et al. [28] showed graded increases in burnout measures across good, fair, and poor health (all *p* < 0.001). That study observed higher emotional exhaustion and depersonalization and lower personal accomplishment (good 17.60 ± 14.04/5.54 ± 6.40/27.70 ± 14.25; fair 26.85 ± 14.90/8.31 ± 7.53/26.53 ± 11.88; poor 36.55 ± 14.38/11.99 ± 9.14/26.05 ± 11.01; all *p* < 0.001). Similarly, in Lovell et al. [44], poorer self-rated health was significantly associated with lower personal accomplishment (*p* = 0.01) alongside recent low-mood frequencies (never 48.2%, sometimes 39.3%, frequently 7.1%, and all the time 2.7%).

Self-perceived health also aligned with occupational intentions: in Nikolić and Višnjić [40], self-rated work ability for physical health demands clustered at the top (“outstanding” 45.6%, “very good” 35.4%), with only 1.3% rating it “bad”. Work ability related to mental health demands was rated “outstanding” by 69.6% and “very good” by 21.5% (bad 1.3%). However, in Rasmus et al. [51], monthly hours worked correlated non-significantly with self-rated physical health (r = −0.121, *p* = 0.171). Workplace violence-related contexts were also intertwined with perceived health. In Meng et al. [45], both physical and verbal violence were associated with self-reported health status (*p* < 0.001 for each), alongside the distribution of global health (good 14.33%, fair 49.06%, bad 36.61%).

#### 3.3.2. Depression of Emergency Workers

Depression and depressive symptoms of emergency workers varied significantly, from 1.77 ± 0.35 (*p* = 0.004) [52] to 7.53 ± 4.98 [47,53]. A relevant proportion of emergency workers had depression in Yan et al. [42,43] and Chen et al. [28] (35.59% in both) and in Jiang et al. [25] (35.7%). In the same study, the severity of depressive symptoms was none (22.3%), mild (42.0%), moderate (19.4%), moderately-severe (10.5%), and severe (5.8%). A pooled meta-analysis of seven studies, conducted under a random-effects model using the DerSimonian-Laird method, estimated a global prevalence of depressive symptoms of 35.1% (95% CI 0.32–0.39) among emergency healthcare professionals, with substantial heterogeneity across studies (I^2^ = 99%). This finding confirms the high and consistent burden of depression observed in individual reports and highlights its strong biopsychosocial component within emergency care settings (Figure 2).

Depression was strongly associated with burnout dimensions, anxiety, sleep disturbances, and resilience-related outcomes. In Chen et al. [28], depressive-symptom prevalence rose stepwise across lower self-perceived health strata: good (17.23%), fair (35.94%), and poor (65.41%) (χ^2^ = 1781.32, *p* < 0.01). Lovell et al. [44] found that recent experiences of depressive symptoms were associated with increased emotional exhaustion (*p* = 0.004) and depersonalization (*p* = 0.049). Gan et al. [46] observed that depressive symptoms reported were significantly associated (*p* < 0.001) with nightmare distress (r = 0.732). Zhang et al. [54] also found that depression significantly correlated (*p* < 0.0001) with workplace violence (r = 0.456), and Jiang et al. [25] showed that self-efficacy was significantly lower (*p* > 0.001) in those with major depression (23.0 ± 6.4) compared to those without it (26.8 ± 5.9).

Depression was also associated with work-related outcomes. It was more prevalent in those who have suffered workplace violence (93.6%) than those who have not (76.0%) [25]; and anxiety and depression also correlated with workplace violence (r = 0.417) [54]. Wu et al. [48] observed that depression was significantly (*p* < 0.0001) more prevalent in those with occupational injuries (87.8%) than those without them (77.5%). Similar findings were shown by Yan et al. [42], who showed that depressive symptoms were significantly (*p* < 0.01) higher in those with turnover intention (70.95%), compared to those without it (38.04%).

On the contrary, Ulmann et al. [39] found that depressive symptoms were lowest in people with over 20 years of experience in emergency departments (V = 0.161, *p* < 0.05). Cascales-Martínez et al. [55] reported that depressive symptom scores were not significantly higher in physicians (1.23 ± 0.29) than nurses (1.13 ± 0.22) and nursing assistants (1.13 ± 0.16) (χ^2^ = 5.97, ε^2^ = 0.05).

#### 3.3.3. Stress of Emergency Workers

Work-related stress was a predominant and pervasive exposure among emergency healthcare professionals. A pooled meta-analysis of seven studies, conducted under a random-effects model, estimated a global prevalence of stress at 74.6% (95% CI 0.36–0.99), confirming its widespread presence across emergency settings. When stratified by severity, pooled proportions were 9.0% (95% CI 0.03–0.17) for low stress, 54.9% (95% CI 0.35–0.74) for moderate stress, and 58.7% (95% CI 0.34–0.81) for severe stress. Heterogeneity was extremely high across all categories (I^2^ > 94%, *p* < 0.001), reflecting contextual diversity and methodological variability among studies.

Publication bias was evaluated using Egger’s or Harbord’s tests, depending on data distribution, and no significant bias was detected (*p* > 0.05).

Collectively, these findings highlight the pervasive psychological strain and sustained exposure to occupational stressors among emergency personnel, underscoring the urgent need for organizational strategies that mitigate workload pressure, emotional fatigue, and role overload in these demanding clinical environments (Figure 2).

In Lei et al. [27], 69.24% reported high work stress, with only a small minority (5.68%) reporting low stress. This high prevalence is consistent with other findings, such as a mean work stress score of 3.05 ± 0.69 on a 1–4 scale [33], 2.9514 ± 0.4457 [38], and the scores obtained in Gan et al. [46]: None *n* = 16 (5.7%), 31.94 ± 8.91; Mild *n* = 16 (5.7%), 32.19 ± 10.36; Moderate *n* = 123 (43.9%), 34.65 ± 10.31; Severe *n* = 125 (44.6%) (38.58 ± 11.63; F = 4.388, *p* = 0.005). Stress recognition averaged 63.44 ± 10.79 in Ramanjaneyulu et al. [35].

In Ma et al. [33], higher stress was directly related to higher turnover intention (r = 0.189) and burnout (r = 0.453). It was also inversely associated with job satisfaction (r = −0.336) and perceived organizational support (r = −0.309). Labrague et al. [38] showed that psychological distress correlated to higher work-family conflict (r = 0.197, *p* < 0.05), lower job satisfaction (r = −0.126, *p* < 0.05), and higher toxic leadership (r = 0.143, *p* < 0.05).

Stress also concentrated around violence-related experiences. Perceived stress due to violence was low in 19.5%, moderate in 55.9%, and high in 22.6% of a cohort [49]. In Choi et al. [31], perceived stress from workplace violence averaged 7.03 ± 2.15 on a 0–10 scale and correlated strongly with immediate responses to violence (r = 0.62, *p* < 0.001) and with long-term strategies (r = 0.41, *p* < 0.001). Stress also showed an independent negative association to workplace violence (β = −0.221; 95% CI −0.341 to −0.101; t = −3.651; *p* < 0.001) [40].

Work stress management interventions were not frequent. Dastur et al. [23], after implementing a self-management and communication skills structured training, reported that occupational stress decreased from 114.39 ± 11.25 to 94.00 ± 12.70 (*p* = 0.117 pre-intervention and *p* = 0.034 post-intervention).

#### 3.3.4. Sleep Quality of Emergency Workers

Sleep quality was consistently fair to poor among emergency department personnel. A pooled meta-analysis of six studies, conducted under a random-effects model, estimated the prevalence of good sleep quality at 12.5% (95% CI 0.10–0.15), fair quality at 42.9% (95% CI 0.36–0.50), and poor quality at 40.1% (95% CI 0.34–0.46). Heterogeneity was extremely high across all categories (I^2^ ≈ 99%, *p* < 0.001), reflecting substantial contextual and methodological variability. Publication bias was assessed using Egger’s or Harbord’s tests, depending on study characteristics, and no significant bias was detected (*p* > 0.05). Overall, these findings confirm that the majority of emergency healthcare professionals experience suboptimal or disturbed sleep, with only a small minority reporting restorative sleep. This pattern underscores the persistent circadian disruption and cumulative fatigue associated with shift work and high job demands in emergency care (Figure 2).

Lei et al. [27] reported that sleep quality was good in 12.59%, general in 51.62%, and bad in 35.79% of participants, underscoring that nearly two-thirds rated their sleep as suboptimal. Similarly, Meng et al. [45] found good sleep in 10.39%, fair in 30.86%, and bad in 58.75%; and Wu et al. [48] reported a five-level distribution consisting of very good 2.5%, good 7.8%, fair 30.9%, bad 36.1%, and very bad 22.7% sleep quality. This was consistent with Yan et al. [42,43], who described sleep quality as good in 15.06%, fair in 48.20%, and poor in 36.74% of the sample.

Component-level differences paralleled these distributions. Ren et al. [52] showed significantly worse subjective sleep quality (1.52 ± 0.48 vs. 0.83 ± 0.22; t = 13.304, *p* < 0.001), longer sleep latency (1.62 ± 0.43 vs. 0.87 ± 0.21; t = 15.980, *p* < 0.001), shorter sleep duration (1.70 ± 0.55 vs. 1.21 ± 0.36; t = 7.695, *p* < 0.001), lower sleep efficiency (0.89 ± 0.22 vs. 0.65 ± 0.15; t = 9.322, *p* < 0.001), more sleep disturbances (1.11 ± 0.32 vs. 0.73 ± 0.19; t = 10.495, *p* < 0.001), and greater use of sleep medications (1.28 ± 0.35 vs. 0.70 ± 0.23; t = 14.298, *p* < 0.001) in emergency department personnel, compared to general department personnel.

The polysomnography carried out in the same study showed shorter total sleep duration (326.43 ± 51.36 vs. 405.47 ± 51.13 min; t = 11.514, *p* < 0.001), longer sleep latency (37.35 ± 10.45 vs. 21.25 ± 6.76 min; t = 13.346, *p* < 0.001), more awakening time (66.42 ± 20.05 vs. 48.30 ± 10.41 min; t = 8.195, *p* < 0.001), and lower sleep efficiency (72.36 ± 7.19% vs. 78.65 ± 6.84%; t = 6.674, *p* < 0.001). Night-time environmental noise correlated strongly with worse overall sleep (total score r = 0.515, *p* < 0.001) and its components: shorter sleep duration (r = −0.503, *p* < 0.001), longer sleep latency (r = 0.422, *p* < 0.001), greater awakening time (r = 0.261, *p* < 0.001), and lower sleep efficiency (r = −0.293, *p* < 0.001) [52].

Yan et al. [42] showed a relationship between sleep quality and turnover intention: 32.03% among those with good sleep, 45.57% with fair sleep, and 62.51% with poor sleep (*p* < 0.01). In Meng et al. [45], sleep quality was associated with both physical violence (*p* < 0.001) and verbal violence (*p* < 0.001). A stepwise rise in occupational injury prevalence was observed in Wu et al. [48]: 59.4% in those with very good, 68.2% good, 74.3% fair, 85.7% bad, and 90.0% very bad sleep (*p* < 0.0001).

#### 3.3.5. Resilience of Emergency Workers

Mean resilience scores varied across studies. A pooled meta-analysis using a random-effects model estimated the prevalence of low resilience at 16.1% (95% CI 0.10–0.29), moderate resilience at 33.9% (95% CI 0.10–0.63), and high resilience at 30.2% (95% CI 0.16–0.45). Heterogeneity was very high across all categories (I^2^ > 94%, *p* < 0.001), reflecting substantial contextual and methodological variability. Publication bias was assessed using Egger’s or Harbord’s tests depending on study distribution, with no significant bias detected in any case (*p* > 0.05).

These findings indicate that although a considerable proportion of emergency professionals display moderate-to-high resilience, notable variability persists across contexts. Overall, resilience emerges as a key psychological resource moderating stress and mitigating burnout risk in emergency healthcare (Figure 2).

Mean resilience scores also varied substantially, 56.81 ± 11.71 (range 0–100) in Choi et al. [31], 81.64 ± 15.26 in Kim et al. [30], 2.72 ± 0.40 (1–4) in Park and Song [56], 27.73 ± 7.51 (0–40) in Liao et al. [57], 81.4 ± 13.1 (26–98) in Lovell et al. [44], and 133.52 ± 7.22 in Sánchez-Zaballos and Mosteiro-Díaz [50]. In that study, overall resilience levels differed by professional category (physicians 134.72 ± 18.26; nurses 128.74 ± 15.76; nursing assistants 130.60 ± 21.11; χ^2^ = 8.84, *p* = 0.01).

Studies reporting categorical profiles described comparable distributions. Lovell et al. [44] reported resilience levels as very low (2.8%), low (7.5%), low-end (11.2%), moderate (19.6%), moderately-high (34.6%), and high (24.3%). Schablon et al. [49] described their sample as having low (28.9%), moderate (22.9%), and high (44.4%) resilience. The proportion of participants across resilience categories also varied by professional role (physicians: very low 7.0%, moderate 69.8%, high 23.3%; nurses: 15.6%/67.5%/16.9%; nursing assistants: 18.8%/45.0%/36.3%; χ^2^ = 18.27, *p* < 0.001) [50].

Sociodemographic and work-related correlates were mixed. Park and Song [56] observed no significant difference by sex (female: 2.71 ± 0.40 vs. male: 2.86 ± 0.40; t = −1.22, *p* = 0.224), whereas Sánchez-Zaballos and Mosteiro-Díaz [50] found distributional differences (female: very low 15.65%, moderate 58.78%, high 25.77%; male: 6.90%/79.31%/13.79%; χ^2^ = 8.60, *p* = 0.01).

Marital status was inconsistently related to resilience. Kim et al. [30] reported higher levels in married compared to single participants (87.90 ± 16.20 vs. 79.80 ± 14.40; t = −2.605, *p* = 0.010). Sánchez-Zaballos and Mosteiro-Díaz [50] also showed single participants had lower resilience than partnered ones (single: 124.38 ± 21.20 vs. partnered: 133.40 ± 16.27; F = 3.69, *p* = 0.01; Bonferroni *p* = 0.02). Conversely, Liao et al. [57] observed the opposite pattern (has spouse: 27.21 ± 7.92 vs. no spouse: 28.52 ± 6.80; t = −2.215, *p* < 0.01).

Regarding professional experience in relation to resilience, Kim et al. [30] reported a non-linear pattern across years of experience (<3: 81.90 ± 15.90; 3–5: 83.10 ± 13.20; 6–7: 79.50 ± 15.90; 8–10: 68.70 ± 11.70; >10: 90.60 ± 14.40; F = 4.772, *p* = 0.001). Work schedule was linked to resilience in Sánchez-Zaballos and Mosteiro-Díaz [50] (no night shifts 126.36 ± 21.58 vs. including night shifts 131.48 ± 17.37; Z = −2.10, *p* = 0.03), with a marked difference among nursing assistants (118.20 ± 24.94 vs. 135.50 ± 17.43; t = −3.08, *p* < 0.001).

Resilience also co-varied with psychosocial resources and performance. In Liao et al. [57], resilience correlated positively with perceived organizational support (r = 0.478) and positive coping (r = 0.427) and negatively with negative coping (r = −0.405; all *p* < 0.01). Kim et al. [30] linked higher resilience to better nursing performance (r = 0.610, *p* < 0.001), while the association with violence experience was non-significant (r = 0.152, *p* = 0.084). Lovell et al. [44] observed lower resilience among participants reporting recent depressive symptoms (*p* < 0.0001).

Violence-related patterns were also shown. Liao et al. [57] found resilience was lower among those encountering physical (26.16 ± 7.50) or combined physical and verbal violence (26.05 ± 7.96) compared with verbal-only exposure (28.12 ± 7.45; F = 3.692, *p* < 0.01). In Choi et al. [31], resilience showed a small, non-significant inverse correlation with staff responses to violence (r = −0.15, *p* = 0.08). Schablon et al. [49] likewise documented substantial recent exposure to aggression alongside resilience distributions skewed toward moderate-high levels (low 28.9%, moderate 22.9%, high 44.4%).

#### 3.3.6. Burnout of Emergency Workers

Burnout was assessed as a multi-dimensional construct encompassing emotional exhaustion, depersonalization, and personal accomplishment, as well as global burnout severity. A pooled meta-analysis using a random-effects model (DerSimonian-Laird method) estimated an overall prevalence of severe burnout of 10.7% (95% CI 0.04–0.20), with very high heterogeneity across studies (I^2^ = 98.6%, *p* < 0.001). No evidence of publication bias was detected (Harbord test, *p* = 0.28).

**Figure 3:**
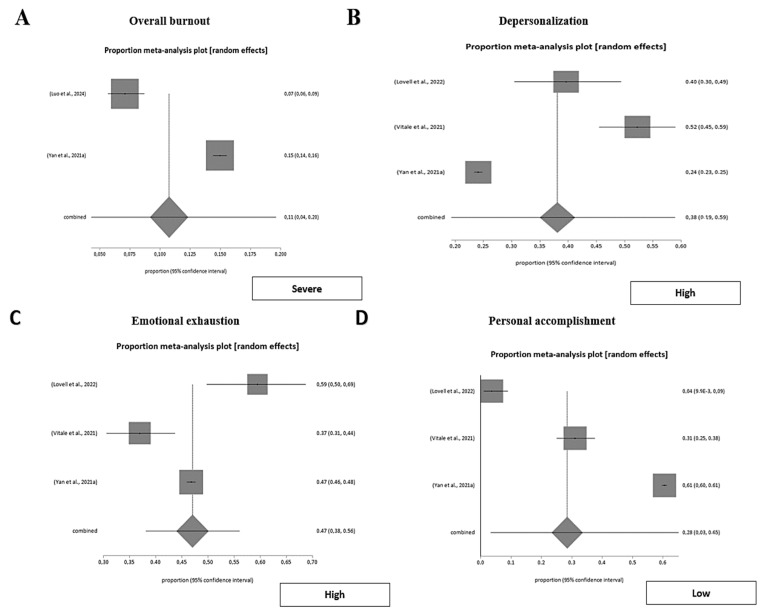

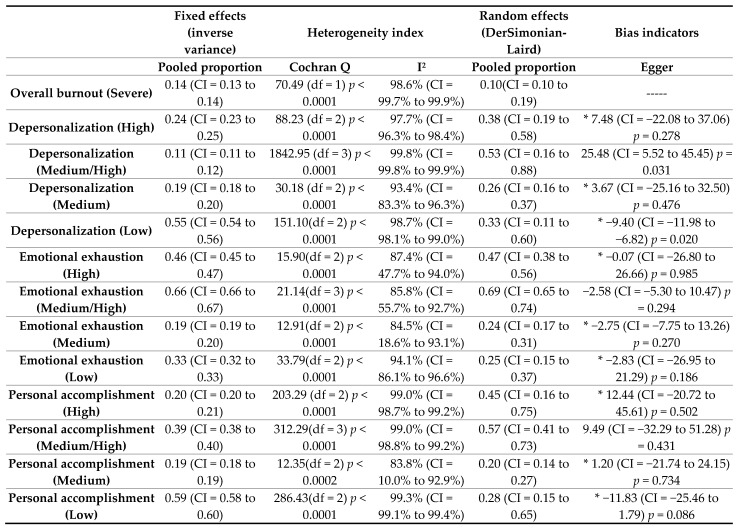
Prevalence of burnout dimensions among emergency healthcare workers. * = Harbord: bias. (**A**) Overall Burnout Forest Plot; (**B**) Depersonalization Forest Plot; (**C**) Emotional Exhaustion Forest Plot; (**D**) Personal Accomplishment Forest Plot [42,44,58,59].

These results confirm that while severe burnout affects roughly one in ten emergency healthcare professionals, considerable variability exists among studies, reflecting differences in measurement tools, sample characteristics, and work contexts. This underscores the importance of analyzing its core dimensions (emotional exhaustion, depersonalization, and personal accomplishment) to capture the full extent of occupational strain (Figure 3).

The latter was prevalent among emergency workers, with rates ranging from moderate to severe across multiple studies. Luo et al. [58] found that 80.65% of their sample met criteria for burnout, with 73.57% experiencing mild to moderate symptoms and 7.08% classified as severe.

Burnout levels were significantly associated with demographic and organizational variables. Younger staff (20–30 years: 2.37 ± 0.86) showed higher overall burnout scores compared to those over 50 years (1.72 ± 0.83), and less experienced professionals (<3 years 2.28 ± 0.76) had higher burnout than those with ≥20 years (1.80 ± 0.90) [58]. Night shift frequency contributed as well: those working ≥13 night shifts per month scored 2.39 ± 0.99 on overall burnout compared to 1.87 ± 0.83 among those without night shifts (F = 12.67, *p* < 0.01).

Workplace structure and environment were contributors to burnout. Munn et al. [60] reported that for every additional 100 emergency department visits, burnout scores increased by 0.46 (95% CI: 0.08 to 0.83, *p* = 0.04), while Schablon et al. [49] observed that 53.3% of emergency personnel felt emotionally exhausted due to workload. Burnout had a negative correlation with perceived organizational support (r = −0.510) [33].

##### Emotional Exhaustion of Emergency Workers

Emotional exhaustion was a prevalent burnout dimension across studies. The pooled proportion of emergency workers presenting moderate-to-high emotional exhaustion was 69.9% (95% CI 0.66–0.74), with substantial heterogeneity (I^2^ = 85.8%, *p* < 0.001). Conversely, low emotional exhaustion was observed in 25.8% (95% CI 0.15–0.38; I^2^ = 87.6%). Publication bias was evaluated using Egger’s and Harbord’s tests, depending on data distribution, and no significant bias was detected (*p* > 0.05). These findings confirm that nearly seven out of ten emergency healthcare professionals experience moderate-to-high emotional exhaustion, underscoring its central role in the burnout syndrome (Figure 3).

Luo et al. [58] reported that 68.46% of participants had moderate to severe exhaustion, and Lee et al. [26] found an average score of 36.77 ± 11.01. Lovell et al. [44] found that 62.3% had high emotional exhaustion, 26.3% moderate, and only 11.3% low. Vitale et al. [59] reported that 36.9% of participants experienced strong levels of emotional exhaustion, 29.3% moderate, and 33.8% weak. Cascales-Martínez et al. [55] observed higher scores in physicians (3.56 ± 1.42) compared to nurses (3.16 ± 1.12) and auxiliaries (2.57 ± 1.24; χ^2^ = 9.54, *p* < 0.05, ε^2^ = 0.08).

Emotional exhaustion was linked to different biopsychosocial and work-related outcomes of emergency workers. Vitale et al. [59] found that emotional exhaustion correlated to sex (*p* = 0.034). Lovell et al. [44] found higher exhaustion in those recently depressed (*p* = 0.004) and those using sleeping aids (*p* = 0.028). Lee et al. [26] reported a strong correlation (r = 0.71, *p* < 0.001) between emotional exhaustion and increased turnover intention. Luo et al. [58] observed that emotional exhaustion increased with night shift frequency (e.g., ≥13 shifts: 2.53 ± 1.33 vs. none: 1.79 ± 1.06, F = 12.23, *p* < 0.01) and with exposure to workplace violence (e.g., <2 weeks ago: 2.87 ± 1.35 vs. never: 1.99 ± 1.15, F = 12.70, *p* < 0.01).

##### Depersonalization of Emergency Workers

The meta-analytic pooled proportion for moderate-to-high depersonalization was 53.1% (95% CI 0.16–0.88), while low depersonalization reached 33.3% (95% CI 0.11–0.60). Both analyses showed very high inconsistency (I^2^ > 97%, *p* < 0.001), reflecting the strong heterogeneity between studies. Publication bias was evaluated using Egger’s and Harbord’s tests, depending on data distribution, and no significant bias was detected (*p* > 0.05). These findings confirm that more than half of emergency healthcare professionals exhibit moderate-to-high depersonalization, a key component of burnout in high-demand clinical settings (Figure 3).

Luo et al. [58] reported moderate to high levels in 53.28% of the sample, and Lee et al. [26] recorded an average score of 16.99 ± 6.17. Lovell et al. [44] found 39.6% with high depersonalization, 43.2% moderate, and only 17.1% low. Vitale et al. [58] reported that 52.3% presented strong symptoms, 19.8% moderate, and 27.9% weak. Cascales-Martínez et al. [55] reported higher depersonalization in physicians (2.69 ± 1.34) than nurses (2.33 ± 1.01) and auxiliaries (2.05 ± 1.21; χ^2^ = 6.99, ε^2^ = 0.06).

Depersonalization was associated with turnover intention (r = 0.36, *p* = 0.002) [26] and psychological distress. Lovell et al. [44] found higher depersonalization in those recently depressed (*p* = 0.049) or using sleeping aids (*p* = 0.034). Luo et al. [58] linked depersonalization to violence exposure: recently exposed staff scored 2.33 ± 1.45 vs. never exposed 1.46 ± 1.04 (F = 14.52, *p* < 0.01).

##### Personal Accomplishment of Emergency Workers

The pooled meta-analysis estimated moderate-to-high personal accomplishment in 57.8% (95% CI 0.41–0.73) of emergency professionals, whereas low accomplishment was observed in 28.5% (95% CI 0.15–0.65). Heterogeneity across studies was extreme (I^2^ ≈ 99%, *p* < 0.001), reflecting substantial methodological and contextual variability among samples. Publication bias was evaluated using Egger’s and Harbord’s tests, depending on data distribution, and no significant bias was detected (*p* > 0.05). These findings suggest that while many emergency healthcare workers maintain a sense of professional fulfilment, a substantial subgroup experiences low accomplishment associated with psychological strain (Figure 3).

Lovell et al. [44] found 80.4% of workers with high accomplishment, 15.9% moderate, and only 3.7% low. However, Luo et al. [58] reported that 27.62% had reduced accomplishment. Cascales-Martínez et al. [55] found no significant differences between professional groups (physicians: 5.07 ± 1.17; nurses: 5.25 ± 1.03; auxiliaries: 5.10 ± 1.43; χ^2^ = 10.30, *p* < 0.05, ε^2^ = 0.08). Vitale et al. [59] reported that 40.5% had strong levels (i.e., better outcomes), 28.4% moderate, and 31.1% weak.

Professional accomplishment was associated with well-being. Lee et al. [26] reported a negative correlation with turnover intention (r = −0.46, *p* < 0.001). Lovell et al. [44] linked reduced accomplishment to poor self-rated health (*p* = 0.01) and depressive symptoms (*p* = 0.019). Luo et al. [58] found that personal accomplishment decreased with age and shift load (e.g., ≥13 night shifts: 2.73 ± 1.47 vs. no shifts: 3.34 ± 1.39; F = 4.12, *p* < 0.01).

##### Interventions for Decreasing Burnout of Emergency Workers

Only one study addressed a coaching-based intervention to manage burnout: Hines-Stellisch et al. [24] reported that emotional exhaustion decreased from 26.1 to 20.7 (Cohen’s d = 0.79), depersonalization remained stable (10.2 to 10.7, d = −0.18), and personal accomplishment increased from 35.4 to 37.6 (d = −0.35).

**Figure 4:**
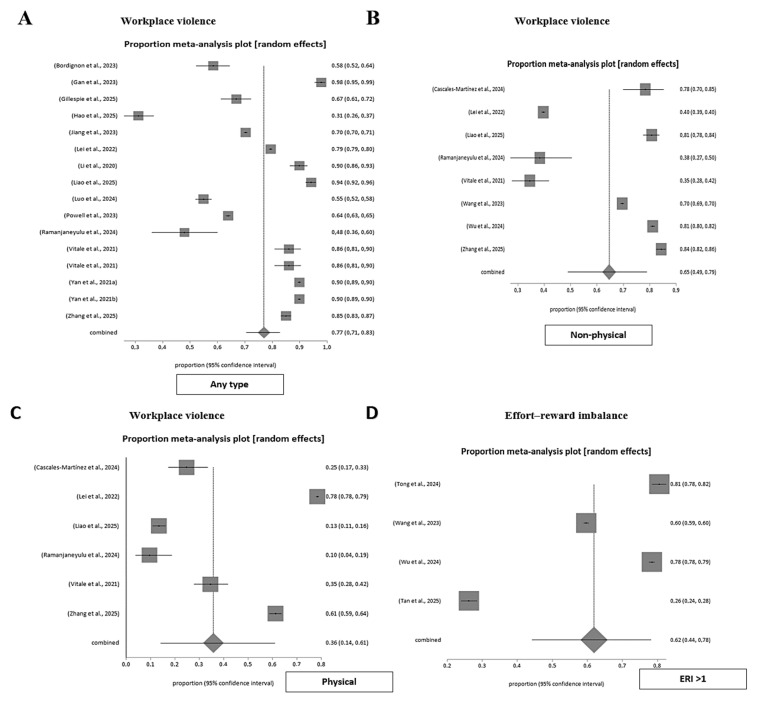

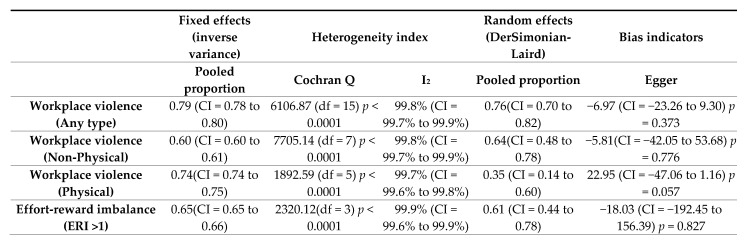
Prevalence of occupational stressors among emergency healthcare workers. (**A**) Workplace Violence (Any Type) Forest Plot; (**B**) Workplace Violence (Non-Physical) Forest Plot; (**C**) Workplace Violence (Physical) Forest Plot; (**D**) Effort-Reward Imbalace Forest Plot [27,34,35,42,43,46,47,48,53,54,55,57,58,59,60,61,62,63,64,65,66].

Across all dimensions, burnout indicators revealed that approximately seven out of ten emergency workers experience moderate-to-high emotional exhaustion, one out of two display significant depersonalization, and nearly six out of ten maintain moderate-to-high levels of personal accomplishment. Despite the substantial heterogeneity between studies, this consistent pattern reflects a pervasive psychological strain among emergency healthcare professionals, partly counterbalanced by the preservation of professional fulfilment and intrinsic motivation (Figure 4).

### 3.4. Work Health of Emergency Workers

Work health of emergency personnel was assessed through workplace violence, effort-reward imbalance, job satisfaction, work engagement, and turnover intention outcomes in included studies.

#### 3.4.1. Workplace Violence in Emergency Departments

Workplace violence is a prevalent and recurring issue across multiple studies. A pooled meta-analysis of sixteen studies estimated that 76.0% (95% CI 0.70–0.82) of emergency healthcare professionals had experienced some form of workplace violence, with very high heterogeneity (I^2^ = 99.8%). When stratified by type, non-physical violence had a pooled prevalence of 64.0% (95% CI 0.48–0.78; I^2^ = 99.9%), whereas physical violence reached 35.0% (95% CI 0.14–0.60; I^2^ = 99.7%). No evidence of small-study or publication bias was found (Egger’s test, *p* = 0.37). These results underscore that verbal and psychological aggression are the most frequent forms of workplace violence encountered in emergency settings (Figure 4).

Most participants (79.39%) from Lei et al. [27] reported having experienced workplace violence, which is similar to the 89.9% observed by Li et al. [63]. Other estimates for ever-exposure ranged from 31.1% [34] to 86.6% in emergency services [22]. Past-year exposure was reported by 70.3% [53], 85.0% [54], and 89.87% [28,42,43] of emergency staff.

Luo et al. [58] found that workplace violence witnessed at work was reported as never (27.1%), less than two weeks ago (10.5%), less than 6 months ago (15.2%), less than one year ago (18.3%), and one year or more ago (28.9%). Personally experienced workplace violence was reported as never (45.1%), less than two weeks ago (3.8%), less than six months ago (6.6%), less than one year ago (11.4%), and one year or more ago (33.1%). In a one-month window, Gillespie et al. [64] found that 97.1% had experienced workplace violence. Gan et al. [46] reported that emergency personnel experienced violence “sometimes” (46.1%) or “always” (40.7%) over recent periods.

Workplace violence is a multifaceted issue encompassing both non-physical and physical aggression. In a large national sample, Lei et al. [27] reported that 79.39% experienced some form of workplace violence, with 78.38% being non-physical and 39.65% physical. The most common form was verbal abuse, affecting 75.22% of participants, with high frequencies reported (once 16.80%, 2–3 times 19.63%, >3 times 38.80%). Threats followed at 51.51% (once 19.97%, 2–3 times 13.74%, >3 times 17.80%), physical assault at 37.40% (once 18.32%, 2–3 times 10.06%, >3 times 9.03%), and verbal sexual harassment at 24.81% (once 10.12%, 2–3 times 5.67%, >3 times 9.02%). In the preceding twelve months, Zhang et al. [54] reported the prevalence of emotional and physical violence at 84.4% and 61.3%, respectively.

Specific types of violence and their reported frequencies varied by time frame. Kim et al. [30] detailed verbal acts per week, with staff reporting cursing (*n* = 72, 55.4% once), talking down (*n* = 51, 39.2% once), screaming (*n* = 59, 45.4% once), and threatening (*n* = 57, 43.8% once). Over a three-month period, Liao et al. [57] found that verbal-only incidents were most common at 80.7%, with physical-only at 13.4% and a combination of both at 5.8%. The frequency of specific physical threats per month included taking a stance to hit (*n* = 55, 42.3% once) and kicking hospital items (*n* = 63, 48.5% once) [30].

Physical and sexual aggression were also documented with specific metrics. Lei et al. [27] quantified physical sexual assault at 12.19% overall. For physical violence per year, Kim et al. [30] reported that the majority had no incidents of throwing an object (*n* = 74, 56.9% none), striking or kicking (*n* = 70, 53.8% none), scratching (*n* = 79, 60.8% none), biting (*n* = 93, 71.5% none), or spitting (*n* = 80, 61.5% none). Park and Song [56] provided a breakdown of average violence frequency per month from patients (verbal 2.61 ± 1.32, psychological 1.57 ± 1.23, physical 0.51 ± 0.73, severe physical 0.07 ± 0.26, sexual harassment 0.36 ± 0.59) and caregivers (verbal 2.49 ± 1.32, psychological 1.37 ± 1.24, physical 0.23 ± 0.58, severe physical 0.05 ± 0.22, sexual harassment 0.15 ± 0.43).

##### Factors Related to Workplace Violence in Emergency Departments

These included perpetrators and biopsychosocial health-related outcomes such as burnout.

The perpetrators of violence were most often patients, but aggression from colleagues and managers was also documented. Nikolić and Višnjić [40] found that threats came most commonly from patients (45.6%) but also from colleagues (2.5%) and managers (1.3%). Munn et al. [67] found that patient/visitor violence predicted higher burnout (+5.74, 95% CI 2.09–9.40; *p* = 0.002), but peer violence showed a similar or larger association (+6.22, 95% CI 2.98–9.46; *p* < 0.001). Violence was frequently tied to specific patient/family dynamics. Ramanjaneyulu et al. [35] found that physical violence (9.59%) and threats (38.36%) were most often attributed to delays in treatment (82.86%), patient death (77.14%), a worsening condition (60.00%), and misperceptions of care (45.71%).

Violence exposure is also linked to poorer mental health. Luo et al. [58] found that staff exposed to workplace violence in the past two weeks had significantly higher overall burnout (2.71 ± 0.75) compared to those never exposed (2.12 ± 0.84, F = 10.42, *p* < 0.01). In the same study, emergency workers with the most recent exposure (less than two weeks) had the highest scores for emotional exhaustion (2.75 ± 1.33 for witnessed; 2.87 ± 1.35 for personally experienced) and depersonalization (2.16 ± 1.32 for witnessed; 2.33 ± 1.45 for personally experienced). Conversely, personal accomplishment scores were lowest for those with the longest duration of exposure (≥1 year) at 2.56 ± 1.45 for witnessed violence and 2.64 ± 1.42 for personally experienced. In Vitale et al. [59], emergency personnel reported lower personal accomplishment (*p* = 0.043) and higher depersonalization (*p* = 0.029) among those reporting aggression. Additionally, Zhang et al. [54] tied workplace violence to somatic symptom burden (r = 0.479) and dyssomnia (r = 0.313) (both *p* < 0.010).

#### 3.4.2. Job Satisfaction of Emergency Workers

**Figure 5:**
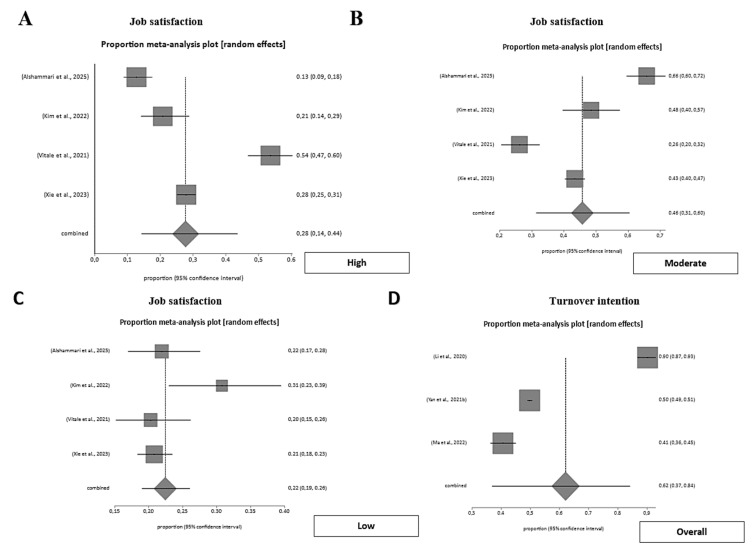

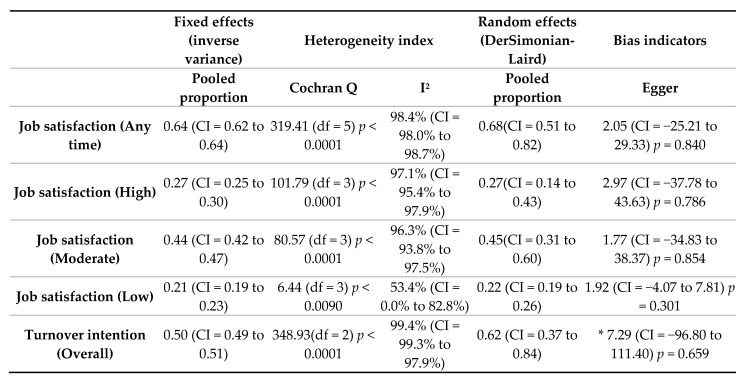
Organizational health and job outcomes among emergency healthcare workers. * = Harbord: bias [30,33,41,43,59,63,68].

Job satisfaction among emergency workers was highly variable across studies. A pooled meta-analysis including six studies estimated an overall job satisfaction prevalence of 68.1% (95% CI 0.52–0.82), with substantial heterogeneity (I^2^ = 98.4%). When stratified by level, low satisfaction was present in 22.4% (95% CI 0.19–0.26), moderate satisfaction in 45.7% (95% CI 0.31–0.60), and high satisfaction in 27.7% (95% CI 0.14–0.44), reflecting a predominance of moderate satisfaction across emergency settings. No significant publication bias was detected (Harbord test, *p* = 0.84). These findings indicate that while most emergency healthcare professionals report at least moderate job satisfaction, notable variability persists across roles and institutions (Figure 5).

Mean job satisfaction scores ranged from 2.48 ± 0.49 in Li et al. [61] to 61.60 ± 13.28 in Ramanjaneyulu et al. [35], with intermediate results such as 3.42 ± 0.62 in Labrague [38], 13.22 ± 3.34 in Wang et al. [47], and 4.39 ± 0.91 in Ma et al. [33]. The highest facet was satisfaction with co-workers (3.69 ± 0.50), whereas the lowest was extrinsic rewards (1.93 ± 0.68).

Categorical profiles showed that 38.2% were satisfied and 62.1% were dissatisfied in Senken et al. [64], and that 65.8% and 70.5% were satisfied in Sánchez Onrubia et al. [69]. Job satisfaction was bad (30.8%), moderate (48.5%), and good (20.7%) in Kim et al. [30]; weak (20.3%), moderate (26.1%), and strong (53.6%) [58]; and very satisfied (3.7%), satisfied (27.9%), normal (43.4%), dissatisfied (20.8%), and very dissatisfied (4.1%) [41].

There was a role-specific job satisfaction of 27.5% in nurses, 45.5% in physicians, 56.9% in learners, and 45.6% in ancillary staff [64]. It differed across physicians, nurses, and auxiliary nursing personnel (3.55 ± 0.84, 4.00 ± 0.68, 3.88 ± 0.50; χ^2^ = 10.37, *p* < 0.050, ε^2^ = 0.08) in Cascales-Martínez et al. [55], as did intrinsic satisfaction: 3.81 ± 0.46, 4.17 ± 0.53, 3.94 ± 0.26; χ^2^ = 12.98, *p* < 0.050, ε^2^ = 0.14), and extrinsic satisfaction (3.28 ± 0.75, 4.02 ± 0.49, 3.89 ± 0.40; χ^2^ = 30.21, *p* < 0.001, ε^2^ = 0.27).

##### Factors Related to Job Satisfaction of Emergency Workers

In Ma et al. [33], job satisfaction was inversely correlated to burnout (r = −0.528), and it showed associations with depersonalization (*p* = 0.004) and personal accomplishment (*p* = 0.002) [47]. Those with compassion fatigue showed shifted distributions compared to those without it (very satisfied 1.3% vs. 11.5%; satisfied 22.9% vs. 43.9%; normal 44.7% vs. 39.3%; dissatisfied 26.0% vs. 4.5%; very dissatisfied 5.2% vs. 0.8%; *p* < 0.001) [41].

Job satisfaction is positively related to perceived organizational support (r = 0.652) [33], organizational commitment (r = 0.440, *p* < 0.010) [60], and higher work ability (β = +0.456, 95% CI 0.031–0.882; t = 2.122; *p* = 0.036) [40]. However, it was related to higher work-family conflict (r = −0.300, *p* < 0.010), and to perceived toxic leadership (r = −0.327, *p* < 0.01) [38]. Those exposed to workplace violence reported lower satisfaction (83.76 ± 20.23) than the non-exposed (85.54 ± 17.64; *p* = 0.040) in Hancerlioglu et al. [22]; and job satisfaction correlated inversely to workplace violence in Li et al. [63] (r = −0.193, *p* < 0.010).

Factors distinguishing dissatisfied and satisfied staff included admission/transfer processes (9.9% vs. 4.2%, *p* < 0.010), boarding (18.2% vs. 2.8%, *p* < 0.010), equipment/stocking/tools (32.8% vs. 24.0%, *p* = 0.010), patient flow (40.3% vs. 32.6%, *p* = 0.040), teaching/learning (8.8% vs. 21.5%, *p* < 0.010), and especially team/coworker interaction (39.0% vs. 85.1%, *p* < 0.010) [70].

#### 3.4.3. Effort-Reward Imbalance in Emergency Workers

Effort-reward imbalance (ERI) was consistently observed among emergency healthcare professionals. A pooled meta-analysis including four large studies estimated that 61.9% (95% CI 0.44–0.78) of emergency workers presented an ERI greater than 1, indicating that perceived effort frequently outweighed received rewards. Heterogeneity was very high (I^2^ = 99.9%, *p* < 0.001), reflecting substantial contextual and methodological variability across studies. No evidence of publication bias was detected (Harbord test, *p* = 0.37). These findings underscore that ERI represents a major occupational stressor in emergency healthcare, associated with sustained imbalance between demands and perceived recognition (Figure 4).

Individual studies reported comparable findings. Tong et al. [65] found a mean ERI score of 0.93 ± 0.57, with 80.5% of participants showing imbalance (>1). Tan et al. [63] also reported a mean of 0.93 ± 0.57, though with 26.2% experiencing ERI > 1. Wang et al. [47] observed a slightly higher mean of 1.24 ± 0.59, with 59.7% reporting imbalance, and Wu et al. [48] found that 78.4% exceeded the ERI threshold.

ERI correlated strongly with both psychosocial and occupational factors. Tong et al. [65] reported significant positive correlations with overall workplace violence (r = 0.552), as well as somatic (r = 0.547) and mental symptoms (r = 0.533; all *p* < 0.01). Similarly, Tan et al. [63] found that effort-reward imbalance was positively associated with emotional exhaustion (r = 0.624), work-family conflict (r = 0.552), somatic symptoms (r = 0.554), and sleep disorders (r = 0.335; all *p* < 0.01).

Overall, these findings demonstrate that ERI represents a major occupational stressor in emergency healthcare settings, with high prevalence and strong links to both mental and physical strain.

#### 3.4.4. Work Engagement of Emergency Workers

Cascales-Martínez et al. [55] found that work engagement was higher among nurses and auxiliary nursing personnel compared to physicians across all dimensions of the Utrecht Work Engagement Scale: Vigor (Medical 4.40 ± 1.77; Nursing 5.14 ± 1.26; Auxiliary 5.58 ± 0.99; χ^2^ = 14.67, *p* < 0.01, ε^2^ = 0.12), Dedication (4.65 ± 1.27; 5.45 ± 1.17; 5.94 ± 0.79; *p* < 0.01, ε^2^ = 0.14), and Absorption (4.07 ± 1.44; 5.05 ± 1.39; 5.31 ± 0.92; *p* < 0.05, ε^2^ = 0.15).

Hu et al. [66] found that heavier overtime was associated with lower scores on all engagement sub-dimensions: Vigor (F = 2.669, *p* < 0.05), Dedication (F = 4.123, *p* < 0.05), and Absorption (F = 3.406, *p* < 0.05). Similarly, Munn et al. [60] found that job resources such as teamwork (+ 0.40), psychological safety (+ 0.41), nurse-manager support (+ 0.32), and sufficient resources (+ 0.34) were all robustly and positively linked to engagement (all *p* < 0.001).

Conversely, higher emergency department volume was associated with lower engagement (−0.02 per +100 visits, *p* = 0.05), and workplace violence, particularly from peers (−0.40, *p* = 0.02), had a negative impact. These findings are consistent with Hu et al. [66], who also reported that engagement tracked strongly with a more favorable organizational climate (Vitality r = 0.536, *p* < 0.001) and was inversely related to workplace psychological violence (Vitality r = −0.331, *p* < 0.001). Munn et al. [60] found that peer violence was linked to lower work engagement (−0.40, 95% CI −0.74 to −0.06; *p* = 0.02).

#### 3.4.5. Turnover Intention of Emergency Workers

Turnover intention was consistently high among emergency personnel. A pooled meta-analysis including three large-scale studies estimated an overall prevalence of turnover intention of 62.1% (95% CI 0.37–0.84), with very high heterogeneity (I^2^ = 99.4%). This finding highlights a critical workforce sustainability challenge, as more than half of emergency healthcare professionals express a desire or intent to leave their position (Figure 5).

Individual studies reported similar findings. Yan et al. [42] observed that 49.75% of participants had turnover intention, while Li et al. [61] reported markedly higher rates (90.2%), including 62.9% high and 27.3% very high turnover intention. Mean turnover intention scores varied widely across studies, ranging from 2.75 ± 0.58 to 17.91 ± 3.82 [26,61]. The multidimensional structure of turnover intention was reflected in subcomponents, such as “possibility of quitting the current job” (mean = 3.01 ± 0.60) and “motivation to look for another job” (mean = 2.53 ± 0.70) [61].

Sociodemographic patterns were mixed. Jiang et al. [53] reported slightly higher turnover intention among males (10.41 ± 3.38) than females (10.15 ± 2.91; t = 3.14, *p* = 0.0017), whereas Park and Song [56] observed the opposite trend (3.16 ± 0.88 vs. 2.48 ± 0.89; t = 2.39, *p* = 0.019). Ma et al. [33] found significant differences by marital status (married: 2.34 ± 0.58 vs. single: 2.49 ± 0.54; F = 7.39, *p* < 0.01).

Turnover intention was strongly related to biopsychosocial outcomes. The proportion of professionals expressing turnover intention increased with poorer self-perceived health (good = 33.59%, fair = 52.34%, poor = 69.75%; *p* < 0.01) and worse sleep quality (good = 32.03%, fair = 45.57%, poor = 62.51%; *p* < 0.01) [42]. Jiang et al. [53] found higher turnover intention among those with fair (10.10 ± 2.75) and poor health (11.41 ± 3.19) compared with good health (9.03 ± 2.68; F = 726.96, *p* < 0.0001).

Correlations with burnout were robust: Ma et al. [33] found r = 0.391, and Lee et al. [26] reported r = 0.43 (*p* < 0.001), particularly for emotional exhaustion (r = 0.71, *p* < 0.001) and depersonalization (r = 0.36, *p* = 0.002), with negative association to personal accomplishment (r = −0.46, *p* < 0.001). Turnover intention was higher among those screening positive for depression (70.95% vs. 38.04%; *p* < 0.01) [42].

Work-related factors also played a significant role. Fei et al. [29] demonstrated a direct link between turnover intention and work-family conflict (total effect b = 0.276, *p* < 0.01) and an indirect pathway via affective states (total indirect b = 0.098, *p* < 0.01). Job satisfaction correlated inversely with turnover intention (r = −0.483, *p* < 0.01 [61]; r = −0.486 [33]).

Exposure to workplace violence was a consistent predictor of turnover intention. Jiang et al. [53] reported significantly higher scores among those exposed (10.52 ± 3.00) than non-exposed (9.38 ± 2.71; t = −24.82, *p* < 0.0001), while Li et al. [61] found a positive correlation (r = 0.145, *p* < 0.01). Similar results were observed in Ma et al. [33] (F = 15.87, *p* < 0.01).

Turnover intention also varied by work experience and shift frequency. Jiang et al. [53] reported higher levels among those with ≤12 months of emergency experience (2.52 ± 0.53) compared with ≥37 months (3.27 ± 0.88; F = 4.31, *p* = 0.016). It increased progressively with more night shifts per month (≤5: 9.80 ± 2.78; 6–10: 10.16 ± 2.96; ≥11: 10.67 ± 3.12; F = 107.00, *p* < 0.0001).

Finally, a structured intervention program demonstrated efficacy in reducing turnover intention: Hines-Stellisch et al. [24] observed a marked reduction from 19.6 to 15.1 (Cohen’s d = 1.37), highlighting the potential of targeted support strategies to mitigate workforce attrition.

## 4. Discussion

Across outcomes, the meta-analytic signal depicts a workforce under sustained biopsychosocial pressure with uneven organizational buffers. Pooled estimates indicate fair self-perceived health ~44% (good ~31%; poor ~16%), severe burnout ~11%, with moderate-high emotional exhaustion ~70% and moderate-high depersonalization ~53%. Depressive symptoms ~35% and work-related stress ~75% (predominantly moderate-severe) co-occur with suboptimal sleep (good ~13%; fair ~43%; poor ~40%). Resilience clusters overall in the moderate-high range (~64%), yet workplace violence is pervasive (~77% overall; ~65% non-physical; ~36% physical). Downstream, job satisfaction ~68% (mostly moderate) contrasts with turnover intention ~62%.

Although heterogeneity was consistently very high (typically I^2^ ≈ 85–99%), small-study/publication bias was not detected on Egger’s or Harbord’s tests across the main pooled outcomes (all *p* > 0.05), supporting the directional robustness of the pattern while signaling substantial contextual variability that policy and service design must address.

The constellation violence → stress/exhaustion/depersonalization → lower satisfaction and higher turnover aligns with effort-reward imbalance (ERI > 1 in ~62%) and points to modifiable organizational levers.

### 4.1. Biopsychosocial Health Outcomes

Emergency workers frequently experience symptoms that affect their psychological, emotional, and physical well-being. Findings included high levels of emotional exhaustion and depersonalization, which reflect the presence of burnout as a widespread condition. These results are consistent with other studies that describe burnout as a progressive response to chronic emotional demands, with emotional fatigue emerging as its most visible component [8,71,72].

Depressive symptoms were also common. Their prevalence aligns with data from other healthcare populations, although emergency professionals appear to face a higher risk due to exposure to intense emotional situations, interpersonal conflict, and lack of recovery time [9,73]. The combination of depression with burnout suggests the existence of complex and reinforcing psychological demands in this setting.

Sleep disturbances were another frequent outcome. Difficulties with sleep initiation, continuity, and restorative quality affected a large portion of the workforce. These findings are in line with international studies that associate rotating shifts and night work with poor sleep hygiene and daytime dysfunction [69,74]. Sleep deficits were often reported alongside other symptoms such as fatigue, low mood, and reduced attention.

Stress appeared across several domains, including perceived pressure, emotional overload, and organizational dissatisfaction [46,49]. This supports the multifactorial nature of stress in emergency contexts, where professionals manage clinical uncertainty, time pressure, and high patient turnover [75].

These findings reinforce the meta-analytic signal that emergency personnel consistently operate under sustained biopsychosocial pressure, with uneven organizational buffers, regardless of country or professional group [76].

### 4.2. Work Health Outcomes

The review also identified a group of outcomes that directly affect the professional functioning and organizational engagement of emergency staff. These conditions influence their ability to remain committed, satisfied, and emotionally available at work.

Violence in the workplace stood out as one of the most recurrent and damaging experiences. Reports of verbal, psychological, and physical aggression were common, and exposure to such events correlated with higher emotional exhaustion, lower job satisfaction, and reduced sleep quality [56,64]. These patterns support the idea that emergency departments often operate in high-risk interpersonal environments [76].

Job satisfaction showed moderate levels but declined sharply under conditions of high stress, burnout, or poor organizational support [33,38]. Conversely, teams with strong leadership, collaboration, and a sense of justice tended to report higher satisfaction and commitment [77]. Work engagement followed a similar distribution. When professionals had adequate resources and psychological safety, they reported more energy and focus at work [67,78].

Turnover intention emerged as a particularly relevant concern. A considerable number of professionals expressed the desire to leave their current positions. These results are consistent with research linking emotional fatigue, organizational inequity, and insufficient institutional support with attrition risk [79].

### 4.3. Integration of Biopsychosocial and Occupational Dimensions

The overlap between personal health and work-related outcomes appeared repeatedly. Emotional exhaustion, depressive symptoms, and sleep difficulties coexisted with job dissatisfaction, decreased engagement, and higher turnover intention. These patterns suggest that professional well-being cannot be separated from workplace conditions [80,81].

Exposure to violence and interpersonal tension contributed to both psychological strain and professional disconnection. High demands without adequate support created a context in which health and occupational risk became interdependent. These findings support the need for integrated strategies that address both individual vulnerability and organizational structure [82].

### 4.4. Limitations and Strengths of the Review

This review was not without limitations. The evidence base was dominated by cross-sectional designs (94%), limiting causal inference and the ability to assess trajectories over time. Grey literature was not included, which may have introduced some publication bias. There was substantial heterogeneity arising from instrument and cut-point variability (e.g., different MBI versions; diverse sleep and stress scales), which likely inflated I^2^. The geographical skew (≈48% of studies from China) may constrain generalizability to other health systems. A notable gap identified in our review is the absence of studies conducted in British Commonwealth countries, which is relevant given that emergency care models, staffing structures, and system pressures in these healthcare systems may differ substantially [83]. Moreover, the pandemic/post-pandemic period (2020–2025) entailed atypical workload patterns that could have amplified several outcomes [84]. Not all endpoints were measured consistently across studies, hindering uniform comparisons.

Despite these constraints, results were directionally consistent across settings. We applied random-effects proportion meta-analyses with the Miller variant of the exact inverse Freeman–Tukey double-arcsine transformation to stabilize variances, and small-study/publication bias tests (Egger and Harbord) were non-significant for the main pooled outcomes, lending confidence to the overall signal. The review’s comprehensive scope, jointly covering biopsychosocial and occupational domains, enabled detection of robust, convergent patterns that are hard to discern in isolated studies [20].

Given the high between-study heterogeneity, there is a need to prioritize longitudinal [85] and interventional designs, measurement harmonization, and pre-specified subgroup/sensitivity analyses (by profession, shift/night-work load, violence exposure, region, and instrument) to refine precision and strengthen causal interpretation.

### 4.5. Nursing Clinical and Research Implications

These findings highlight the urgent need to improve working conditions and promote the psychological well-being of emergency healthcare professionals. Emotional exhaustion, depression, and workplace violence emerge as structural issues that require sustained organizational commitment rather than isolated interventions. Healthcare institutions should integrate monitoring of staff well-being and psychosocial risks into their routine quality and safety systems, ensuring that prevention and support mechanisms are accessible and visible [86].

Efforts to strengthen resilience and reduce occupational stress should focus on safer and more predictable schedules, recognition and reward structures that balance effort with value, and environments that foster psychological safety, teamwork, and peer support. Proactive detection and targeted intervention for burnout, depression, and sleep disturbance are also essential to maintaining workforce stability [87].

From a research perspective, longitudinal and mixed-method designs are needed to better understand how organizational conditions shape professional adaptation and health over time [85]. Future research should examine how restructuring emergency care models, such as integrated care approaches, multidisciplinary triage, or distributed leadership frameworks, may alleviate biopsychosocial strain. Collaborative intersectoral models, including crisis/street triage and mental-health crisis pathways, warrant evaluation for their potential to reduce emergency department overload [84]. It is also essential to explore organizational strategies that promote communication, team cohesion, and personalization. Additional research is needed to understand how job satisfaction, reward systems, and locus of control influence burnout trajectories and retention [88]. Finally, studies should analyze how chronic mental-health presentations (e.g., suicidality, substance misuse, personality disorders) contribute to workplace violence and emotional fatigue and how structural rather than isolated organizational strategies could mitigate these effects [89].

Protecting the biopsychosocial well-being of professionals is fundamental to sustaining high-quality, humane, and resilient emergency care systems.

## 5. Conclusions

Emergency healthcare professionals face high burdens across biopsychosocial and work-health domains: prominent burnout, depressive symptoms, elevated stress, and poor sleep, alongside widespread workplace violence, effort-reward imbalance, and substantial turnover intention. While heterogeneity across studies is large, the direction and magnitude of effects are remarkably consistent and not explained by publication bias, underscoring the need for system-level interventions.

Sustainable emergency services require protecting and resourcing the workforce. Embedding violence prevention, sleep-supportive rostering, ERI-aware reward structures, and psychologically safe leadership into routine operations should be prioritized. Future longitudinal and interventional research should clarify causal pathways and identify high-yield organizational levers to improve staff well-being, retention, and patient care quality.

## Data Availability

All data are available upon request to the corresponding authors.

## Supplementary Materials

The following supporting information can be downloaded at: https://www.mdpi.com/article/10.3390/nursrep15120430/s1, Table S1: Search Strategies Across Databases; Table S2: Summary of Findings.

## Public Involvement Statement

No public involvement in any aspect of this research.

## Guidelines and Standards Statement

This manuscript was drafted against the PRISMA 2020 Guidelines for Systematic Reviews research.

## Abbreviations

The following abbreviations are used in this manuscript:

APA	American Psychological Association.
CBI	Copenhagen Burnout Inventory.
CI	Confidence Interval.
ED	Emergency Department.
ERI	Effort-Reward Imbalance.
GHQ-28	General Health Questionnaire—28 items.
HABS-U/HABS-CS	Hospital Aggressive Behaviour Scale—Users/Coworkers and Superiors.
I^2^	Between-study heterogeneity index (I-squared).
JBI	Joanna Briggs Institute.
κ (Kappa)	Inter-rater agreement coefficient.
MBI/MBI-GS	Maslach Burnout Inventory/General Survey.
MeSH	Medical Subject Headings.
MSQ	Minnesota Satisfaction Questionnaire.
n/A	Not Applicable.
*n*	Sample size.
PHQ-9	Patient Health Questionnaire—9 items.
PIO	Person, Intervention, and Outcomes framework.
PRISMA	Preferred Reporting Items for Systematic Reviews and Meta-Analyses.
PROSPERO	International Prospective Register of Systematic Reviews.
QNWL	Quality of Nursing Work Life.
RS-13	Resilience Scale—13 items.
UWES/UWES-9	Utrecht Work Engagement Scale (9 items).
WAI	Work Ability Index.
WFCS/WFBRC-S	Work-Family Conflict Scale/Work-Family Behavioral Role Conflict Scale.
WHO	World Health Organization.
WPVB	Workplace Psychologically Violent Behaviors instrument.
χ^2^ (Chi-square)	Chi-square test statistic.
ε^2^ (Eta-squared)	Effect size (Eta-squared).
*p*	Statistical significance level.
M/SD/IQR	Mean/Standard Deviation/Interquartile Range.

## References

[1] World Health Organization (1948). Summary Report on Proceedings, Minutes and Final Acts of the International Health Conference Held in New York from 19 June to 22 July 1946.

[2] Huber M., Knottnerus J.A., Green L., van der Horst H., Jadad A.R., Kromhout D., Leonard B., Lorig K., Loureiro M.I., van der Meer J.W.M. (2011). How Should We Define Health?. BMJ.

[3] Kirsten W. (2024). The Evolution from Occupational Health to Healthy Workplaces. Am. J. Lifestyle Med..

[4] Flaubert J.L., Menestrel S.L., Williams D.R., Wakefield M.K., National Academies of Sciences, Engineering, and Medicine, National Academy of Medicine, Committee on the Future of Nursing 2020–2030 (2021). Supporting the Health and Professional Well-Being of Nurses. The Future of Nursing 2020–2030: Charting a Path to Achieve Health Equity.

[5] Timbol C.R., Baraniuk J.N. (2019). Chronic Fatigue Syndrome in the Emergency Department. Open Access Emerg. Med..

[6] Morse G., Salyers M.P., Rollins A.L., Monroe-DeVita M., Pfahler C. (2012). Burnout in Mental Health Services: A Review of the Problem and Its Remediation. Adm. Policy Ment. Health Ment. Health Serv. Res..

[7] Jackson D., Firtko A., Edenborough M. (2007). Personal Resilience as a Strategy for Surviving and Thriving in the Face of Workplace Adversity: A Literature Review. J. Adv. Nurs..

[8] Maslach C., Jackson S.E. (1981). The Measurement of Experienced Burnout. J. Organ. Behav..

[9] Pappa D., Koutelekos I., Evangelou E., Dousis E., Mangoulia P., Gerogianni G., Zartaloudi A., Toulia G., Kelesi M., Margari N. (2023). Investigation of Nurses’ Wellbeing towards Errors in Clinical Practice—The Role of Resilience. Medicina.

[10] Gómez-Urquiza J.L., De La Fuente-Solana E.I., Albendín-García L., Vargas-Pecino C., Ortega-Campos E.M., Cañadas-De la Fuente G.A. (2017). Prevalence of Burnout Syndrome in Emergency Nurses: A Meta-Analysis. Crit. Care Nurse.

[11] Gómez-Salgado J., Domínguez-Salas S., Romero-Martín M., Romero A., Coronado-Vázquez V., Ruiz-Frutos C. (2021). Work Engagement and Psychological Distress of Health Professionals during the COVID-19 Pandemic. J. Nurs. Manag..

[12] Higgins J.P.T., Thomas J., Chandler J., Cumpston M., Li T., Page M.J., Welch V.A. (2024). Cochrane Handbook for Systematic Reviews of Interventions.

[13] Page M.J., McKenzie J.E., Bossuyt P.M., Boutron I., Hoffmann T.C., Mulrow C.D., Shamseer L., Tetzlaff J.M., Akl E.A., Brennan S.E. (2021). The PRISMA 2020 Statement: An Updated Guideline for Reporting Systematic Reviews. BMJ.

[14] Shea B.J., Reeves B.C., Wells G., Thuku M., Hamel C., Moran J., Moher D., Tugwell P., Welch V., Kristjansson E. (2017). AMSTAR 2: A Critical Appraisal Tool for Systematic Reviews That Include Randomised or Non-Randomised Studies of Healthcare Interventions, or Both. BMJ.

[15] JBI 2025 JBI Critical Appraisal Tools. https://jbi.global/critical-appraisal-tools.

[16] Tawfik G.M., Dila K.A.S., Mohamed M.Y.F., Tam D.N.H., Kien N.D., Ahmed A.M., Huy N.T. (2019). A Step by Step Guide for Conducting a Systematic Review and Meta-Analysis with Simulation Data. Trop. Med. Health.

[17] Skelly A.C., Dettori J.R., Brodt E.D. (2012). Assessing Bias: The Importance of Considering Confounding. Evid. Based Spine Care J..

[18] McHugh M.L. (2012). Interrater Reliability: The Kappa Statistic. Biochem. Med..

[19] Zhai C., Guyatt G. (2024). Fixed-Effect and Random-Effects Models in Meta-Analysis. Chin. Med. J..

[20] Schwarzer G., Rücker G., Evangelou E., Veroniki A.A. (2022). Meta-Analysis of Proportions. Meta-Research: Methods and Protocols.

[21] von Hippel P.T. (2015). The Heterogeneity Statistic I2 Can Be Biased in Small Meta-Analyses. BMC Med. Res. Methodol..

[22] Hancerlioglu S., Konakci G., Ozturk S., Kiyan G.S. (2020). Does Workplace Violence Reduce Job Satisfaction Levels of Emergency Service Workers?. Int. J. Caring Sci..

[23] Dastur S., Zandi M., Karimian M. (2024). The Effect of Virtual Self-Management Training in Communication Skills on The Level of Violence and Occupational Stress of Emergency Technicians at Shahid Beheshti University of Medical Sciences in 2021. J. Health Saf. Work..

[24] Hines-Stellisch K., Gawlik K.S., Teall A.M., Tucker S. (2024). Implementation of Coaching to Address Burnout in Emergency Clinicians. J. Emerg. Nurs..

[25] Jiang N., Chen H., Yin X., Wang J., Wu Y., Tian M., Zhang J., Chen Z., Wu J., Lv C. (2024). Comparison of Depressive Symptoms among Emergency Physicians and the General Population in China: A Cross-Sectional Study Based on National Data. Hum. Resour. Health.

[26] Lee M.M.D., Gensimore M.M., Maduro R.S., Morgan M.K., Zimbro K.S. (2021). The Impact of Burnout on Emergency Nurses’ Intent to Leave: A Cross-Sectional Survey. J. Emerg. Nurs..

[27] Lei Z., Yan S., Jiang H., Feng J., Han S., Herath C., Shen X., Min R., Lv C., Gan Y. (2022). Prevalence and Risk Factors of Workplace Violence against Emergency Department Nurses in China. Int. J. Public Health.

[28] Chen Y., Shen X., Feng J., Lei Z., Zhang W., Song X., Lv C. (2022). Prevalence and Predictors of Depression among Emergency Physicians: A National Cross-Sectional Study. BMC Psychiatry.

[29] Fei Y., Jiang N., Zhao H., Zhang F., Fu W., Yin X. (2023). How Work-Family Conflict Influences Emergency Department Nurses’ Turnover Intention: The Mediating Role of Positive and Negative Affect. Int. Emerg. Nurs..

[30] Kim S., Gu M., Sok S. (2022). Relationships between Violence Experience, Resilience, and the Nursing Performance of Emergency Room Nurses in South Korea. Int. J. Environ. Res. Public Health.

[31] Choi S.-Y., Kim H., Park K.-H. (2022). Experience of Violence and Factors Influencing Response to Violence Among Emergency Nurses in South Korea: Perspectives on Stress-Coping Theory. J. Emerg. Nurs..

[32] Huang H., Li F., Jiang Y. (2024). Connor Davidson Resilience Scores, Perceived Organizational Support and Workplace Violence among Emergency Nurses. Int. Emerg. Nurs..

[33] Ma Y., Chen F., Xing D., Meng Q., Zhang Y. (2022). Study on the Associated Factors of Turnover Intention among Emergency Nurses in China and the Relationship between Major Factors. Int. Emerg. Nurs..

[34] Hao X., Dai Y., Jia S., Liu S., Zhao C., Liu X. (2025). Latent Profile Analysis of Mental Workload among Emergency Department Nurses: A Cross-Sectional Study. BMC Nurs..

[35] Ramanjaneyulu E., Kumar B.S., Rao B.V., Kumar M.V.K. (2024). Safety Attitudes and Workplace Violence Among Emergency Room Doctors: A Cross-Sectional Study. Int. J. Med. Public Health.

[36] Atta M.H.R., Elsayed S.M., El-Gazar H.E., Abdelhafez N.G.E., Zoromba M.A. (2025). Role of Violence Exposure on Altruistic Behavior and Grit among Emergency Nurses in Rural Hospitals. Int. Nurs. Rev..

[37] Kousha S., Shahrami A., Forouzanfar M.M., Sanaie N., Atashzadeh-Shoorideh F., Skerrett V. (2022). Effectiveness of Educational Intervention and Cognitive Rehearsal on Perceived Incivility among Emergency Nurses: A Randomized Controlled Trial. BMC Nurs..

[38] Labrague L.J. (2024). Linking Toxic Leadership with Work Satisfaction and Psychological Distress in Emergency Nurses: The Mediating Role of Work-Family Conflict. J. Emerg. Nurs..

[39] Ulmann R., Zeller A., Hirt J. (2025). Self-rated health of emergency nurses-a cross-sectional study. Notf. Rettungsmedizin.

[40] Nikolić D., Višnjić A. (2020). Mobbing and Violence at Work as Hidden Stressors and Work Ability Among Emergency Medical Doctors in Serbia. Medicina.

[41] Xie W., Liu M., Okoli C.T.C., Zeng L., Huang S., Ye X., Liu F., Wang J. (2023). Construction and Evaluation of a Predictive Model for Compassion Fatigue among Emergency Department Nurses: A Cross-Sectional Study. Int. J. Nurs. Stud..

[42] Yan S., Shen X., Wang R., Luo Z., Han X., Gan Y., Lv C. (2021). The Prevalence of Turnover Intention and Influencing Factors among Emergency Physicians: A National Observation. Hum. Resour. Health.

[43] Yan S., Shen X., Wang R., Luo Z., Han X., Gan Y., Lv C. (2021). Challenges Faced by Emergency Physicians in China: An Observation from the Perspective of Burnout. Front. Psychiatry.

[44] Lovell L.-M.P., Atherley A.E.N., Watson H.R., King R.D. (2022). An Exploration of Burnout and Resilience among Emergency Physicians at Three Teaching Hospitals in the English-Speaking Caribbean: A Cross-Sectional Survey. Lancet Reg. Health–Am..

[45] Meng Y., Wang J., Jiang N., Gong Y., Ye F., Li J., Zhou P., Yin X. (2023). Occurrence and Correlated Factors of Physical and Verbal Violence among Emergency Physicians in China. J. Glob. Health.

[46] Gan Q.-W., Yu R., Lian Z.-R., Yuan Y.-L., Li Y.-P., Zheng L.-L. (2023). Relationship between Nightmare Distress and Depressive Symptoms in Chinese Emergency Department Nurses: A Cross-Sectional Study. World J. Psychiatry.

[47] Wang J., Mu K., Gong Y., Wu J., Chen Z., Jiang N., Zhang G., Lv C., Yin X. (2023). Occurrence of Self-perceived Medical Errors and Its Related Influencing Factors among Emergency Department Nurses. J. Clin. Nurs..

[48] Wu J., Wang J., Li Q., Gong Y., Luo J., Yin X. (2024). Prevalence of Occupational Injury and Its Associated Factors among Emergency Department Physicians in China: A Large Sample, Cross-Sectional Study. Prev. Med..

[49] Schablon A., Kersten J.F., Nienhaus A., Kottkamp H.W., Schnieder W., Ullrich G., Schäfer K., Ritzenhöfer L., Peters C., Wirth T. (2022). Risk of Burnout among Emergency Department Staff as a Result of Violence and Aggression from Patients and Their Relatives. Int. J. Environ. Res. Public Health.

[50] Sánchez-Zaballos M., Mosteiro-Díaz M.P. (2021). Resilience Among Professional Health Workers in Emergency Services. J. Emerg. Nurs..

[51] Rasmus P., Marcinkowska W., Cieleban N., Lipert A. (2020). Workload and coping with stress and the health status of emergency medical staff in the context of work-life balance. Med. Pract..

[52] Ren D., Liu Y., Xu L. (2025). Effects of Long-Term Noise Exposure on Mental Health and Sleep Quality of Emergency Medical Staff and Coping Strategies. Noise Health.

[53] Jiang N., Zhou X., Gong Y., Tian M., Wu Y., Zhang J., Chen Z., Wang J., Wu J., Yin X. (2023). Factors Related to Turnover Intention among Emergency Department Nurses in China: A Nationwide Cross-sectional Study. Nurs. Crit. Care.

[54] Zhang H., Zhou J., Zhong L., Zhu L., Chen X. (2025). Relationship between Workplace Violence and Occupational Health in Emergency Nurses: The Mediating Role of Dyssomnia. Nurs. Crit. Care.

[55] Cascales-Martínez A., López-Ros P., Pina D., Cánovas-Pallares J.M., López López R., Puente-López E., Piserra Bolaños C. (2024). Differences in Workplace Violence and Health Variables among Professionals in a Hospital Emergency Department: A Descriptive-Comparative Study. PLoS ONE.

[56] Park J.E., Song M.R. (2023). Effects of Emergency Nurses’ Experiences of Violence, Resilience, and Nursing Work Environment on Turnover Intention: A Cross-Sectional Survey. J. Emerg. Nurs..

[57] Liao L., Wu Q., Su Y., Li R., Wang L. (2025). Coping Styles Mediated the Association Between Perceived Organizational Support and Resilience in Emergency Nurses Exposed to Workplace Violence: A Cross-Sectional Study. Nurs. Health Sci..

[58] Luo L., Li J., Wu F., Peng X., Zhou F. (2024). Investigation of occupational burnout status and influencing factors among emergency department healthcare workers using the MBI-GS Scale. Zhong Nan Da Xue Xue Bao Yi Xue Ban.

[59] Vitale E., Lupo R., Calabro A., Cornacchia M., Conte L., Marchisio D., Caldararo C., Carvello M., Carriero M.C. (2021). Mapping Potential Risk Factors in Developing Burnout Syndrome between Physicians and Registered Nurses Suffering from an Aggression in Italian Emergency Departments. J. Psychopathol..

[60] Bordignon M., Marziale M.H.P., Sutherland M.A., Monteiro I. (2023). Factors Related to Work Ability among Nursing Professionals from Urgent and Emergency Care Units: A Cross-Sectional Study. Work.

[61] Powell J.R., Cash R.E., Kurth J.D., Gage C.B., Mercer C.B., Panchal A.R. (2023). National Examination of Occupational Hazards in Emergency Medical Services. Occup. Environ. Med..

[62] Tang M., Liu L., Cai J., Yang Y. (2024). Effect of Noise in the Emergency Department on Occupational Burnout and Resignation Intention of Medical Staff. Noise Health.

[63] Li N., Zhang L., Xiao G., Chen Z.J., Lu Q. (2020). Effects of Organizational Commitment, Job Satisfaction and Workplace Violence on Turnover Intention of Emergency Nurses: A Cross-sectional Study. Int. J. Nurs. Pract..

[64] Gillespie G.L., Cooper S.S., Bresler S.A., Tamsukhin S. (2025). Emergency Department Workers’ Perceived Support and Emotional Impact After Workplace Violence. J. Forensic Nurs..

[65] Tong L., Zhu L., Zhang H., Zhong L., Diao D., Chen X., Zhang J. (2024). Effort-Reward Imbalance and Health Outcomes in Emergency Nurses: The Mediating Role of Work-Family Conflict and Intrinsic Effort. Front. Public Health.

[66] Tan Y., Zhou J., Zhang H., Lan L., Chen X., Yu X., Zhong L., Zhu L., Gao Y. (2025). Effects of Effort-Reward Imbalance on Emergency Nurses’ Health: A Mediating and Moderating Role of Emotional Exhaustion and Work-Family Conflict. Front. Public Health.

[67] Munn L.T., O’Connell N., Huffman C., McDonald S., Gibbs M., Miller C., Danhauer S.C., Reed M., Mason L., Foley K.L. (2025). Job-Related Factors Associated with Burnout and Work Engagement in Emergency Nurses: Evidence to Inform Systems-Focused Interventions. J. Emerg. Nurs..

[68] Alshammari M., Aljarash A., Alharbi J., Alrashdi A., Alshammari K., Alshammeri R., Alanezi S., Qaladi O.A. (2025). Quality of Work Life and Related Factors among Nurses in Emergency and Critical Care Departments in Saudi Arabia in 2023: A Cross-Sectional Study. Nurs. Midwifery Stud..

[69] Sánchez Onrubia I.M., Resta Sánchez E.J., Cabañero Contreras T., Perona Moratalla A.B., Molina Alarcón M. (2025). Bienestar, Burnout y Sueño Del Personal de Enfermería de Urgencias En Turnos de 12 Horas. Enfermería Clínica.

[70] Senken B., Welch J., Sarmiento E., Weinstein E., Cushman E., Kelker H. (2024). Factors Influencing Emergency Medicine Worker Shift Satisfaction: A Rapid Assessment of Wellness in the Emergency Department. J. Am. Coll. Emerg. Physicians Open.

[71] Isa K.Q., Ibrahim M.A., Abdul-Manan H.-H., Mohd-Salleh Z.-A.H., Abdul-Mumin K.H., Rahman H.A. (2019). Strategies Used to Cope with Stress by Emergency and Critical Care Nurses. Br. J. Nurs..

[72] Yuwanich N., Sandmark H., Akhavan S. (2016). Emergency Department Nurses’ Experiences of Occupational Stress: A Qualitative Study from a Public Hospital in Bangkok, Thailand. Work.

[73] Westphal M., Bingisser M.-B., Feng T., Wall M., Blakley E., Bingisser R., Kleim B. (2015). Protective Benefits of Mindfulness in Emergency Room Personnel. J. Affect. Disord..

[74] James S.M., Honn K.A., Gaddameedhi S., Van Dongen H.P.A. (2017). Shift Work: Disrupted Circadian Rhythms and Sleep—Implications for Health and Well-Being. Curr. Sleep Med. Rep..

[75] González-Pascual M., Pérez-Ferreiro M., Rodríguez de Castro S., Cerro-González M.D.C., Recio-Vivas A.M. (2025). Occupational Stress in Healthcare Professionals in Spain: A Multicenter Study. Hisp. Health Care Int..

[76] Naidoo R., Schoeman R. (2023). Burnout in Emergency Department Staff: The Prevalence and Barriers to Intervention. S. Afr. J. Psychiatry.

[77] Colquitt J.A., Conlon D.E., Wesson M.J., Porter C.O., Ng K.Y. (2001). Justice at the Millennium: A Meta-Analytic Review of 25 Years of Organizational Justice Research. J. Appl. Psychol..

[78] Hu H., Gong H., Ma D., Wu X. (2022). Association between Workplace Psychological Violence and Work Engagement among Emergency Nurses: The Mediating Effect of Organizational Climate. PLoS ONE.

[79] Middleton K. (2023). Why Working Expectations Need to Change to Protect Doctors and the Quality of Patient Care: A Perspective from Down-Under. J. Pediatr. Rehabit. Med..

[80] Hall L.H., Johnson J., Watt I., Tsipa A., O’Connor D.B. (2016). Healthcare Staff Wellbeing, Burnout, and Patient Safety: A Systematic Review. PLoS ONE.

[81] Montgomery A., Panagopoulou E., Esmail A., Richards T., Maslach C. (2019). Burnout in Healthcare: The Case for Organisational Change. BMJ.

[82] Rudkjoebing L.A., Bungum A.B., Flachs E.M., Eller N.H., Borritz M., Aust B., Rugulies R., Rod N.H., Biering K., Bonde J.P. (2020). Work-Related Exposure to Violence or Threats and Risk of Mental Disorders and Symptoms: A Systematic Review and Meta-Analysis. Scand. J. Work Environ. Health.

[83] Anandaciva S. (2023). How Does the NHS Compare to the Health Care Systems of Other Countries?.

[84] Pourmand A., Caggiula A., Barnett J., Ghassemi M., Shesser R. (2023). Rethinking Traditional Emergency Department Care Models in a Post-Coronavirus Disease-2019 World. J. Emerg. Nurs..

[85] Savitz D.A., Wellenius G.A. (2023). Can Cross-Sectional Studies Contribute to Causal Inference? It Depends. Am. J. Epidemiol..

[86] Çakır O., Akkoç İ., Arun K., Dığrak E., Uluırmak Ünlüeroğlugil H.S. (2025). The Complex Dynamics of Violence and Burnout in Healthcare: A Closer Look at Physicians and Nurses. Int. Nurs. Rev..

[87] Pollock A., Campbell P., Cheyne J., Cowie J., Davis B., McCallum J., McGill K., Elders A., Hagen S., McClurg D. (2020). Interventions to Support the Resilience and Mental Health of Frontline Health and Social Care Professionals during and after a Disease Outbreak, Epidemic or Pandemic: A Mixed Methods Systematic Review. Cochrane Database Syst. Rev..

[88] Firmansyah M.E., Isnanto S.H. (2025). The Influence of Job Satisfaction and Reward Systems on Turnover Intention. Dinasti Int. J. Manag. Sci..

[89] Ministry of Health (2022). Mental Health Strategy of the National Health System 2022–2026.

