# Supplementary material for: Biopsychosocial and Occupational Health of Emergency Healthcare Professionals: A Systematic Review and Meta-Analysis
Source: Nurs Rep. 2025 Dec 4;15(12):430. doi: 10.3390/nursrep15120430 (PMC12735800; doi:10.3390/nursrep15120430)
Supplement: Supplementary file 1 [file nursrep-15-00430-s001.zip › R1 Supplementary Material 1.pdf]

Supplementary Material Table S1

Search Strategies Across Databases

Search Strategy	Databases
("Occupational health" OR "Workplace health" OR "Job stress" OR "Workplace safety" OR "Work environment" OR "Occupational risks" OR "Occupational diseases" OR "Health workforce") AND ("Traumatology" OR "Trauma care" OR "Emergency departments" OR "Emergency services" OR "Urgent care" OR "Emergency Medical Services" OR "Emer-gency Nursing" OR "Emergency Service, Hospital") AND ("Physical health" OR "Mental health" OR "Psychological stress" OR "Work-related stress" OR "Burnout" OR "Musculo-skeletal disorders" OR "Occupational injuries" OR "Fatigue" OR "Resilience" OR "labor abandonment" OR Absenteeism OR "Change of service" OR "Intention to leave" OR "Personnel turnover") AND ("Interventions" OR "Workplace interventions" OR "Well-being programs" OR "Stress management" OR "Preventive strategies" OR "Occu-pational safety interventions") AND (Nurs* OR "Trauma nursing" OR "Emergency nurs*") NOT ("surg*" OR "child*" OR "protocol*" OR "review*")	PubMed, Embase, Web of Science, CINAHL, APA PsycInfo, APA PsycArticles
("Occupational health" OR "Workplace health" OR "Job stress" OR "Workplace safety" OR "Work environment" OR "Occupational risks" OR "Occupational diseases" OR "Health workforce") AND ("Traumatology" OR "Trauma care" OR "Emergency departments" OR "Emergency services" OR "Urgent care" OR "Emergency Medical Services" OR "Emer-gency Nursing" OR "Emergency Service, Hospital") AND ("Physical health" OR "Mental health" OR "Psychological stress" OR "Work-related stress" OR "Burnout" OR "Musculo-skeletal disorders" OR "Occupational injuries" OR "Fatigue" OR "Resilience" OR "labor abandonment" OR Absenteeism OR "Change of service" OR "Intention to leave" OR "Personnel turnover") AND ("Interventions" OR "Workplace interventions" OR "Well-being programs" OR "Stress management" OR "Preventive strategies" OR "Occu-pational safety interventions") AND (Nurs* OR "Trauma nursing" OR "Emergency nurs*") AND NOT ("surg*" OR "child*" OR "protocol*" OR "review*")	Scopus

Note. Authors◎own elaboration.
