# Supplementary material for: Biopsychosocial and Occupational Health of Emergency Healthcare Professionals: A Systematic Review and Meta-Analysis
Source: Nurs Rep. 2025 Dec 4;15(12):430. doi: 10.3390/nursrep15120430 (PMC12735800; doi:10.3390/nursrep15120430)
Supplement: Supplementary file 1 [file nursrep-15-00430-s001.zip › R1 Supplementary Material 2.pdf]

Supplementary Material Table S2

Summary of Findings

Main author, year, region, and cite	Design, intervention, main variables, sample, JBI score	Aim	Evaluation instruments	Main findings and conclusions
Alshammari et al., [68]<br><br>2025<br><br>Saudi Arabia	Cross-sectional, descriptive study<br><br>N/A<br><br>Age, gender, educational level, years of experience, work area, quality of nursing work life, satisfaction levels<br><br>n=251<br><br>6/8	To assess the quality of nursing work life and its associated factors among clinical nurses working in intensive care units and emergency departments	Brook's Quality of Nursing Work Life (QNWL) questionnaire	*Sample characteristics*<br>Age: mean 32.3 ± 5.7 years<br>Gender: n=176 females (70.1%), n=75 males (29.9%)<br>Years of experience: mean 9.2 ± 6.1 years<br><br>*QNWL*<br>Total QNWL score: mean 172.1 ± 27.4 (moderate level)<br>Work life/home life subscale: mean 42.7 ± 7.9<br>Work design subscale: mean 45.6 ± 8.2<br>Work context subscale: mean 53.8 ± 9.1<br>Work world subscale: mean 29.9 ± 6.7<br><br>*Distribution of satisfaction levels (frequencies by categories)*<br>Overall QNWL: n=164 (65.3%) moderate, n=55 (21.9%) low, n=32 (12.7%) high<br>Home/work life: n=178 (70.9%) moderate, n=48 (19.1%) low, n=25 (10.0%) high<br>Work design: n=185 (73.7%) moderate, n=36 (14.3%) low, n=30 (12.0%) high<br>Work environment/context: n=139 (55.4%) moderate, n=64 (25.5%) low, n=48 (19.1%) high<br>Work world: n=170 (67.7%) high, n=53 (21.1%) moderate, n=28 (11.2%) low<br><br>*Comparisons*<br>Age: significant predictor of total QNWL (p = 0.021)<br>Work area (intensive care unit vs emergency department): not significant (p = 0.271).<br>Gender: not significant (p = 0.116).<br>Years of experience: not significant (p = 0.208).<br>Family responsibilities (caring for children/parents): not significant (p = 0.392).
Atta et al.,[36]<br><br>2025<br><br>Saudi Arabia	Cross-sectional, descriptive study<br><br>N/A	To explore the effect of violence exposure on altruistic behavior and grit among	Workplace place violence questionnaire<br>Short Grit Scale	*Sample characteristics*<br>Age: mean 23.07 ± 6.3 years<br>Years of experience: mean 3.18 ± 3.2 years<br>Gender: n=72 males (23.1%), n=240 females (76.9%)<br>Marital status: n=259 single (83.0%), n=53 married (17.0%)

Age, years of experience, gender, marital status, job title, hospital unit, work shift, forms of violence, impact of violence, reporting of violence, mitigation strategies, violence risk factors, altruism, grit<br><br>n=312<br><br>8/8	emergency nurses in rural hospitals	Altruistic behaviors scale	Work shift: n=192 morning (61.5%), n=31 afternoon (9.9%), n=41 night (13.1%), n=48 unspecified (15.4%)<br><br>*Workplace violence exposure*<br>Gender<br>Forms of violence: Male 2.0 ± 1.1, Female 2.0 ± 0.9; t=0.472, p=0.637<br>Impact of violent incidences: Male 1.8 ± 0.6, Female 2.1 ± 0.6; t=3.208, p=0.002<br>Reporting of incidence: Male 2.3 ± 0.5, Female 2.3 ± 0.4; t=0.009, p=0.993<br>Mitigation strategies: Male 2.4 ± 0.5, Female 2.6 ± 0.5; t=3.271, p=0.001<br>Risk factors: Male 2.3 ± 0.5, Female 2.3 ± 0.5; t=0.491, p=0.625<br><br>Marital status<br>Forms of violence: Single 1.9 ± 0.9, Married 2.4 ± 1.1; t=3.372, p=0.001<br>Impact of violent incidences: Single 2.0 ± 0.6, Married 2.0 ± 0.6; t=0.183, p=0.856<br>Reporting of incidence: Single 2.2 ± 0.4, Married 2.4 ± 0.5; t=3.039, p=0.003<br>Mitigation strategies: Single 2.6 ± 0.5, Married 2.4 ± 0.5; t=2.102, p=0.039<br>Risk factors: Single 2.3 ± 0.5, Married 2.4 ± 0.4; t=1.379, p=0.169<br><br>Work shift<br>Forms of violence: Morning 2.0 ± 0.9, Afternoon 1.9 ± 0.9, Night 1.8 ± 1.0, Not specified 2.2 ± 1.1; F=1.400, p=0.243<br>Impact of violent incidences: Morning 2.0 ± 0.6, Afternoon 1.9 ± 0.7, Night 2.0 ± 0.6, Not specified 2.0 ± 0.7; F=0.285, p=0.837<br>Reporting of incidence: Morning 2.3 ± 0.4, Afternoon 2.1 ± 0.5, Night 2.3 ± 0.6, Not specified 2.3 ± 0.4; F=1.280, p=0.281<br>Mitigation strategies: Morning 2.6 ± 0.5, Afternoon 2.6 ± 0.5, Night 2.4 ± 0.5, Not specified 2.5 ± 0.5; F=1.857, p=0.137<br>Risk factors: Morning 2.2 ± 0.5, Afternoon 2.3 ± 0.5, Night 2.4 ± 0.5, Not specified 2.3 ± 0.4; F=1.355, p=0.257<br><br>*Altruism and grit*<br>Gender<br>Altruism: Male 2.6 ± 0.8, Female 2.6 ± 0.8; t=0.149, p=0.874<br>Grit: Male 3.0 ± 0.9, Female 2.8 ± 0.7; t=1.681, p=0.096<br><br>Marital status<br>Altruism: Single 2.6 ± 0.8, Married 2.5 ± 0.8; t=1.100, p=0.257

				Grit: Single 2.8 ± 0.7, Married 3.0 ± 0.9; t=1.810, p=0.071<br><br>Work shift<br>Altruism: Morning 2.6 ± 0.7, Afternoon 2.5 ± 0.9, Night 2.6 ± 0.8, Not specified 2.3 ± 0.8; F=2.143, p=0.055<br>Grit: Morning 2.8 ± 0.6, Afternoon 2.6 ± 0.7, Night 3.0 ± 0.7, Not specified 2.7 ± 1.1; F=2.561, p=0.055<br><br>*Correlations*<br>Age and years of experience: r = 0.431, p<0.01<br>Years of experience and altruism: r = −0.122, p<0.05<br>Altruism and grit: r = 0.477, p<0.01<br>Years of experience and grit: r = 0.183, p<0.01<br>Forms of violence and age: r = 0.188, p<0.01<br>Forms of violence and years of experience: r = 0.271, p<0.01<br>Impact of violent incidences and grit: r = −0.273, p<0.01<br>Reporting of incidence and altruism: r = −0.134, p<0.05<br>Reporting of incidence and grit: r = −0.196, p<0.01<br>Mitigation strategies and altruism: r = −0.258, p<0.01<br>Mitigation strategies and grit: r = −0.357, p<0.01<br>Risk factors and altruism: r = −0.204, p<0.01<br>Risk factors and grit: r = −0.257, p<0.01
Bordignon et al.,[60]<br><br>2023<br><br>Brazil	Cross-sectional, descriptive study<br><br>N/A<br><br>Professional type, age, workplace violence role, workplace violence type, Work Ability Index<br><br>n=267<br><br>7/8	To identify levels and predictors of work ability among Brazilian nursing professionals.	Work Ability Index (WAI)<br><br>Custom-tailored violence at work questionnaire	*Sample characteristics*<br>Age (n=262): <30 n=43 (16.4%); 30–39 n=102 (38.9%); 40–49 n=76 (29.0%); 50–59 n=34 (13.0%); ≥60 n=7 (2.7%). Mean age 39.2 ± 9.9 years (range 21–68).<br>Workplace violence (past year)<br>Victim (n=257): One type n=110 (42.8%); Verbal abuse + physical n=30 (11.7%); Verbal abuse + sexual harassment n=7 (2.7%); Sexual harassment + physical n=1 (0.4%); Three types n=8 (3.1%).<br>Witness (n=249): One type n=78 (31.3%); Two or more types n=103 (41.4%)<br><br>*Work Ability Index*<br>Mean 40.4 ± 5.5 (range 22–49).<br>WAI categories (n=265): Excellent n=87 (32.8%); Good n=124 (46.8%); Moderate n=47 (17.7%); Poor n=7 (2.6%). (Good+Excellent = n=211, 79.6%).

Cascales-Martínez et al.,[55]<br><br>2024<br><br>Spain	Cross-sectional, descriptive study<br><br>N/A<br><br>Age, gender, civil status, professional group, speciality, seniority in post, years of experience, work shift, sick leaves in the last twelve monthos<br><br>n=120<br><br>6/8	To analyze the differences between different professionals in exposure to violence in the workplace and burnout, job satisfaction, engagement, and general health.	Aggressive behavior scale for health care workers users (HABS-U)<br><br>Aggressive behavior scale hospital: Coworkers and superiors (HABS-CS)<br><br>Goldberg General Health Questionnaire version 28 items (GHQ-28).<br><br>Maslach Burnout Inventory-General Survey (MBI-GS).<br><br>Utrecht Work Engagement Scale (UWES-9).<br><br>Minnesota Satisfaction Questionnaire (MSQ)	*Sample characteristics*<br>Age: 41.8 ± 10.8 years.<br>Gender: Female: n=74 (61.7%); Male: n=46 (38.3%).<br>Marital status: Married/partner n=59 (49.1%); Single n=53 (44.2%); Divorced/widowed n=8 (6.7%).<br>Job seniority (years): 0–2 n=26 (21.7%); 3–5 n=24 (20.0%); 6–10 n=19 (15.8%); 11–15 n=38 (31.7%); Missing n=13 (10.8%).<br>Years of experience: 0–10 n=44 (36.6%); 11–20 n=46 (38.3%); 21–30 n=11 (9.2%); >30 n=6 (5.0%); Missing n=13 (10.9%).<br>Work shift: 12 h n=68 (56.7%); 7 h n=18 (15.0%); Others n=27 (22.5%); Missing n=7 (5.8%).<br>Sick leave in the last twelve months: Yes n=20 (20.6%); No n=70 (72.2%); Missing n=30 (7.2%)<br><br>*Exposure to workplace violence in the last year (by professional group)*<br>User non-physical violence: Medical 90.09% (n=51/57); Nursing 96.42% (n=27/28); Aux 100% (n=16/16)<br>User physical violence: Medical 25.45% (n=15/57); Nursing 25.00% (n=7/28); Aux 50.00% (n=8/16)<br>Lateral (coworker) personal violence: Medical 47.27% (n=27/57); Nursing 46.42% (n=13/28); Aux 68.75% (n=11/16)<br>Lateral relational violence: Medical 14.54% (n=8/57); Nursing 14.20% (n=4/28); Aux 31.25% (n=5/16)<br>Lateral workplace violence: Medical 16.36% (n=9/57); Nursing 17.80% (n=5/28); Aux 31.25% (n=5/16)<br><br>*Health and violence -related outcomes by professional group*<br>(A=Medical; B=Nursing; C=Auxiliary nursing)<br><br>User violence<br>Physical: A 1.27 ± 0.54; B 1.30 ± 0.45; C 1.67 ± 0.93; χ^2=6.99, ε^2=0.06<br>Non-physical: A 3.08 ± 1.27; B 3.36 ± 1.19; C 2.96 ± 1.45; χ^2=13.36, p<.01, ε^2=0.11<br><br>Lateral (coworker) violence<br>Personal: A 1.50 ± 0.62; B 1.48 ± 0.65; C 1.90 ± 0.85; χ^2=6.01, ε^2=0.05, post-hoc: ns.<br>Relational: A 1.14 ± 0.24; B 1.15 ± 0.38; C 1.46 ± 0.74; χ^2=5.60, ε^2=0.04, ns.<br>Workplace: A 1.17 ± 0.42; B 1.19 ± 0.38; C 1.49 ± 0.93; ; χ^2=6.07, ε^2=0.05, ns

				General health (GHQ-28 subscales)<br>Depression: A 1.23 ± 0.29; B 1.13 ± 0.22; C 1.13 ± 0.16; χ^2=5.97, ε^2=0.05, ns.<br>Anxiety/insomnia: A 2.11 ± 0.83; B 2.01 ± 0.67; C 2.01 ± 0.70; χ^2=0.49, ε^2=0.004, ns.<br>Somatic symptoms: A 2.04 ± 0.73; B 1.96 ± 0.52; C 2.00 ± 0.66; χ^2=1.00, ε^2=0.008, ns.<br><br>Burnout (MBI-GS)<br>Emotional exhaustion: A 3.56 ± 1.42; B 3.16 ± 1.12; C 2.57 ± 1.24; χ^2=9.54, p<.05, ε^2=0.08.<br>Professional efficacy: A 5.07 ± 1.17; B 5.25 ± 1.03; C 5.10 ± 1.43; χ^2=10.30, p<.05, ε^2=0.08<br>Cynicism: A 2.69 ± 1.34; B 2.33 ± 1.01; C 2.05 ± 1.21; χ^2=6.99, ε^2=0.06, ns.<br><br>Engagement (UWES-9)<br>Vigor: A 4.40 ± 1.77; B 5.14 ± 1.26; C 5.58 ± 0.99; χ^2=14.67, p<.01, ε^2=0.12<br>Dedication: A 4.65 ± 1.27; B 5.45 ± 1.17; C 5.94 ± 0.79; χ^2=17.25, p<.01, ε^2=0.14<br>Absorption: A 4.07 ± 1.44; B 5.05 ± 1.39; C 5.31 ± 0.92; χ^2=16.81, p<.05, ε^2=0.15<br><br>Job satisfaction (MSQ-short)<br>Intrinsic: A 3.81 ± 0.46; B 4.17 ± 0.53; C 3.94 ± 0.26; χ^2=12.98, p<.05, ε^2=0.14<br>Extrinsic: A 3.28 ± 0.75; B 4.02 ± 0.49; C 3.89 ± 0.40; χ^2=30.21, p<.001, ε^2=0.27<br>General: A 3.55 ± 0.84; B 4.00 ± 0.68; C 3.88 ± 0.50; χ^2=10.37, p<.05, ε^2=0.08.
Chen et al., [28]<br><br>2022<br><br>China	Cross-sectional, descriptive study<br><br>N/A<br><br>Gender, age, marital status, work tenure, hospital level, work shift, workplace violence in the last twelve months, self-perceived health, sleep quality, Workplace Violence Scale, Center for	To explore the prevalence and influencing factors of depression among emergency physicians	Workplace Violence Scale (WVS)<br><br>Center for Epidemiological Studies Depression scale (CES-D)	*Sample characteristics*<br>Age: 37.66 ± 8.06 years.<br>Workplace violence in past 12 months: n=13,699 (89.87%).<br>Shift work: n=13,288 (87.17%).<br><br>*General depression scores and depression scores within groups*<br>Depression (CES-D ≥ 20): n=5,425 (35.59%).<br><br>Gender: Male n=10,650 (69.87%), depressed n=3,854 (36.19%); Female n=4,593 (30.13%), depressed n=1,571 (34.20%); χ^2=5.50, p=0.02.<br><br>Age groups: ≤31 n=4,089 (26.83%), depressed n=1,467 (35.88%); >31–≤37 n=4,117 (27.01%), 1,674 (40.66%); >37–≤43 n=3,291 (21.59%), 1,236 (37.56%); >43 n=3,746 (24.57%), 1,048 (27.98%); χ^2=146.60, p<0.01.<br><br>Marital status: Married/widowed/divorced n=12,691 (83.26%), 4,494 (35.41%); Unmarried n=2,552 (16.74%), 931 (36.48%); χ^2=1.06, p=0.30.

	Epidemiological Studies Depression scale<br><br>n=15243<br><br>8/8			Work tenure (years): ≤3 y n=4,921 (32.28%), 1,660 (33.73%); >3–≤6 n=3,114 (20.44%), 1,173 (37.67%); >6–≤11 n=3,424 (22.46%), 1,351 (39.46%); >11 n=3,784 (24.82%), 1,241 (32.80%); χ^2=48.49, p<0.01.<br><br>Hospital level: Three-grade n=10,152 (66.60%), 3,661 (36.06%); Two-grade n=4,841 (31.76%), 1,708 (35.28%); Other n=250 (1.64%), 56 (22.40%); χ^2=20.16, p<0.01.<br><br>Shift work: Yes n=13,288 (87.17%), 5,031 (37.86%); No n=1,955 (12.83%), 394 (20.15%); χ^2=233.12, p<0.01<br><br>Workplace violence in the last twelve months: Yes n=13,699 (89.87%), 5,267 (38.45%); No n=1,544 (10.13%), 158 (10.23%); χ^2=481.88, p<0.01.<br><br>Self-perceived health: Good n=4,707 (30.88%), 811 (17.23%); Fair n=7,729 (50.71%), 2,778 (35.94%); Poor n=2,807 (18.41%), 1,836 (65.41%); χ^2=1,781.32, p<0.01.<br><br>Sleep quality: Good n=2,295 (15.06%), 250 (10.89%); Fair n=7,347 (48.20%), 2,058 (28.01%); Poor n=5,601 (36.74%), 3,117 (55.65%); χ^2=1,777.99, p<0.01.
Choi et al., [31]<br><br>2022<br><br>South Korea	Cross-sectional, descriptive study<br><br>N/A<br><br>Sex, age, marital status, months of experience, seniority in post, presence of workplace violence protocols, experience of violence, perceived stress, coping actions, resilience, response to violence	To measure the experience of violence, perceived stress, coping actions after violence, resilience and responses to violence	Experience of Violence<br><br>A custom-tailored perceived stress question<br><br>Assault Response Questionnaire<br><br>Connor-Davidson Resilience Scale	*Sample characteristics*<br>Sex: Female n=125 (95.4%); Male n=6 (4.6%).<br>Age (years, categories): ≤25 n=24 (18.3%); 26–30 n=55 (42.0%); 31–35 n=32 (24.4%); ≥36 n=20 (15.3%).<br>Marital status: Single n=101 (77.1%); Married n=30 (22.9%).<br>Months of experience: ≤12 n=22 (16.8%); 13–60 n=46 (35.1%); 60–120 n=35 (26.7%); >120 n=28 (21.4%).<br>Seniority in post (months): ≤12 n=33 (25.2%); 13–60 n=67 (51.1%); 60–120 n=27 (20.6%); >120 n=4 (3.1%).<br>Workplace violence policy present at hospital: Yes n=83 (63.4%); No n=48 (36.6%).<br><br>*Violence-related outcomes*<br>Experience of violence (total): 18.76 ± 9.31 (0–48).<br>Verbal 7.05 ± 3.06 (0–12); Physical threats 7.83 ± 3.76 (0–15); Physical violence 3.88 ± 4.50 (0–21).<br>Perceived stress from workplace violence (0–10): 7.03 ± 2.15.<br>Coping actions after violence (0–27): 15.56 ± 3.67.<br>Resilience (0–100): 56.81 ± 11.71.

	n=131<br><br>8/8			Response to violence (26–130): 80.80 ± 19.00; subscales—Biophysiological 26.65 ± 9.30, Emotional 47.56 ± 9.60, Social 6.60 ± 2.85<br><br>*Correlations with response to violence*<br>Experience of violence: r=0.43, p<.001.<br>Perceived stress: r=0.62, p<.001.<br>Coping actions after violence: r=0.23, p=0.009.<br>Resilience: r=−0.15, p=0.08 (ns).
Dastur et al., [23]<br><br>2024<br><br>Iran	Longitudinal, quasi-experimental study with two groups<br><br>6 weeks of virtual self-management training in communication skills (WhatsApp; weekly modules covering communication skills, anger control, violence assessment, stress control). Follow-up at 2 months post-intervention.<br><br>Occupational stress, workplace violence<br><br>n=54 (n=28 intervention; n=26 control)<br><br>7/9	To investigate the impact of a virtual self-management training in communication skills on occupational stress and the degree of aggression among emergency technicians.	Hospital Occupational Stress Scale (HSS-35)<br><br>Workplace Violence in the Health Sector Questionnaire	*Sample characteristics*<br>Not reported<br><br>*Occupational stress levels by categorical levels*<br>Before intervention<br>Intervention (n=28): Low n=0 (0%); Moderate n=7 (25.0%); Severe n=21 (75.0%).<br>Control (n=26): Low n=0 (0%); Moderate n=8 (30.8%); Severe n=18 (69.2%).<br>Between-group p=0.636 (ns).<br><br>After intervention<br>Intervention (n=28): Low n=1 (3.6%); Moderate n=21 (75.0%); Severe n=6 (21.4%).<br>Control (n=26): Low n=6 (23.1%); Moderate n=4 (15.4%); Severe n=16 (61.5%).<br>Between-group p=0.001.<br>Within-group: Intervention p=0.001; Control p=0.077<br><br>*Occupational stress' mean scores*<br>Pre: Intervention 114.39 ± 11.25 vs Control 110.08 ± 11.92; between-group p=0.117 (ns).<br>Post: Intervention 94.00 ± 12.70 vs Control 98.81 ± 20.81; between-group p=0.034.<br>Within-group: Intervention p=0.001; Control p=0.077.<br><br>*Change in stress score (before and after) by subgroup*<br>Sex<br>Intervention: Male −24.55 ± 18.76 vs Female −17.71 ± 16.01; p=0.285.<br>Control: Male −2.22 ± 17.52 vs Female −16.06 ± 20.26; p=0.066.<br><br>Marital status<br>Intervention: Single −31.00 ± 27.73 vs Married −19.12 ± 15.84; p=0.572.<br>Control: Single −11.86 ± 24.73 vs Married −11.05 ± 18.97; p=0.866.

				Shift work<br>Intervention: Fixed −28.00 ± 14.78 vs Rotating −18.74 ± 17.46; p=0.215.<br>Control: Fixed −27.50 ± 21.92 vs Rotating −9.92 ± 19.89; p=0.185.
Fei et al., [29]<br><br>2023<br><br>China	Cross-sectional, descriptive study<br><br>N/A<br><br>Gender, age, marital status, work-family conflict, positive affect, negative affect, turnover intention<br><br>n=17582<br><br>8/8	To investigate the effects of work-family conflict on emergency nurses' turnover intention and the role of affect between work-family conflict and turnover intention	Work Family Conflict Scale (WFCS)<br><br>Positive and Negative Affect Scale (PANAS)<br><br>Turnover Intention scale	*Sample characteristics*<br>Gender (n=17,582): Male n=1,803 (10.3%); Female n=15,779 (89.8%).<br>Age groups (n=17,582): <25 y n=3,493 (19.9%); 25–34 y n=10,540 (60.0%); >34 y n=3,549 (20.2%).<br>Marital status: Unmarried n=6,623 (37.7%); Married n=10,868 (61.9%).<br><br>*Scale descriptives*<br>Work-family conflict: 17.55 ± 4.01.<br>Positive affect: 16.47 ± 3.56.<br>Negative affect: 16.26 ± 3.74.<br>Turnover intention: 10.18 ± 2.96.<br><br>*Correlations (Spearman; p<0.05 for marked values):*<br>Work-family conflict and Positive affect r = −0.300*<br>Work-family conflict and Negative affect r = 0.421*<br>Work-family conflict and turnover intention r = 0.350*<br>Positive affect and Negative affect r = −0.349*<br>Positive affect and turnover intention r = −0.384*<br>Negative affect and turnover intention r = 0.282*
Gan et al., [46]<br><br>2023<br><br>China	Cross-sectional, descriptive study<br><br>N/A<br><br>Sex, age, marital status, years of experience, night shifts per month, work pressure, workplace violence frequency, self-reported health,	To understand the relationship between nightmare distress and depressive symptoms among emergency department nurses in China	Chinese Version of the Nightmare Distress Questionnaire (NDQ-CV)<br><br>Center for Epidemiological Studies Depression Scale (CES-D)	*Sample characteristics*<br>Sex: Male n=54 (19.3%), 42.39 ± 10.83; Female n=226 (80.7%), 34.61 ± 10.59;t=4.828, p<0.001.<br>Age (years): ≤25 n=55 (19.6%), 37.55 ± 12.52; 26–35 n=124 (44.3%), 35.63 ± 11.56; 36–45 n=88 (31.4%), 35.78 ± 9.70; >45 n=13 (4.6%), 36.85 ± 8.36;F=0.430, p=0.732.<br>Marital status: Unmarried n=92 (32.9%), 37.13 ± 12.27; Married n=177 (63.2%), 35.42 ± 10.48; Divorced/Widowed n=11 (3.9%), 38.73 ± 8.87;F=1.048, p=0.352.<br>Years of experience: <1 n=10 (3.6%), 33.70 ± 11.94; 1–5 n=98 (35.0%), 38.92 ± 12.13; 6–10 n=61 (21.8%), 35.41 ± 11.38; >10 n=111 (39.6%), 34.23 ± 9.30;F=3.507, p=0.016.<br>Night shifts per month: 0–5 n=69 (24.6%), 36.62 ± 9.48; 6–10 n=130 (46.4%), 37.13 ± 11.55; >10 n=81 (28.9%), 34.04 ± 11.33;F=2.069, p=0.128.

	nightmare distress, depression<br><br>n=280<br><br>8/8			Work pressure: None n=16 (5.7%), 31.94 ± 8.91; Mild n=16 (5.7%), 32.19 ± 10.36; Moderate n=123 (43.9%), 34.65 ± 10.31; Severe n=125 (44.6%), 38.58 ± 11.63;F=4.388, p=0.005.<br>Workplace violence frequency: Never n=6 (2.1%), 34.33 ± 11.99; Rarely n=31 (11.1%), 35.06 ± 11.97; Sometimes n=129 (46.1%), 36.03 ± 11.55; Always n=114 (40.7%), 36.58 ± 10.27;F=0.213, p=0.887.<br>Self-reported health: Poor n=110 (39.3%), 38.75 ± 11.62; Average n=133 (47.5%), 34.65 ± 10.25; Good n=37 (13.2%), 33.49 ± 10.82;F=5.517, p=0.004.<br><br>*Correlations among NDQ-CV and CES-D (all p<0.001)*<br>NDQ-CV total and CES-D total: r = 0.732.<br>NDQ-CV total and Depressed affect: r = 0.727.<br>NDQ-CV total and Somatic symptoms: r = 0.737.<br>NDQ-CV total and Interpersonal difficulties: r = 0.647.<br>NDQ-CV total and Positive affect: r = −0.406.
Gillespie et al., [64]<br><br>2025<br><br>United States of America	Cross-sectional, descriptive study<br><br>N/A<br><br>Race, gender, profession, shift, perceived violence and support, emotional impact of violence<br><br>n=206<br><br>6/8	To examine emergency departments workers' perceived level of workplace violence support and emotional affects that workplace violence causes them	Survey of Violence Experienced by Staff<br><br>Custom-tailored Likert-type scale for the need for support after workplace violence and their perceived satisfaction of support after workplace violence	*Sample characteristics*<br>Female n=156 (75.7%); Registered nurse n=122 (59.8%); Primary shift: Day n=76 (43.4%), Night n=64 (36.6%<br>Experienced workplace violence in the past 30 days n=198 (97.1%).<br><br>*Perceived availability and need for support after workplace violence*<br>“Support available to me when needed”: Disagree n=23 (11.2%), Neutral n=52 (25.5%), Agree n=129 (63.2%).<br>“Staff verbally abused need support”: Disagree n=12 (5.9%), Neutral n=27 (13.2%), Agree n=166 (81.0%).<br>“Staff threatened need support”: Disagree n=4 (2.0%), Neutral n=19 (9.2%), Agree n=183 (88.8%).<br>“Staff physically assaulted need support”: Disagree n=3 (1.5%), Neutral n=7 (3.4%), Agree n=196 (95.1%).<br>“Verbal abuse, threats, and physical assault require different supports”: Disagree n=30 (14.8%), Neutral n=52 (25.6%), Agree n=121 (59.6%).<br>“It is difficult to access appropriate supports when necessary”: Disagree n=85 (42.5%), Neutral n=77 (38.5%), Agree n=38 (19.0%).<br><br>*Emotional impact of workplace violence (0-100; higher score being worse)*<br>Verbal abuse vs threats/assaults (independent t test): Verbal 33.5 ± 29.1 (n=89) vs Threats/assaults 34.1 ± 31.0 (n=117); t(204)=−0.135, p=0.893 (ns).

				Among those with all three workplace violence types (n=46): Kendall's W=0.034, p=0.212 (no concordance of emotional-impact ranks across verbal, threat, assault)
Hancerlioglu et al., [22]<br><br>2020<br><br>Turkey	Cross-sectional, descriptive study<br><br>N/A<br><br>Age, gender, marital status, years of experience, duty, workplace violence exposure; impact of feelings motivation, satisfaction<br><br>n=136<br><br>3/8	To evaluate the exposure to workplace violence of emergency service workers and its effects on job satisfaction.	Custom-tailored workplace violence structured self-administered questionnaire<br><br>Job Satisfaction Scale	*Sample characteristics*<br>Age: 30.94 ± 6.77 years.<br>Gender: Male n=72 (52.9%), Female n=64 (47.1%).<br>Marital status: Single n=83 (61.0%), Married n=53 (39.0%).<br>Years of experience in the emergency department: 3.91 ± 4.01.<br><br>*Workplace violence exposure in the emergency department*<br>Ever exposed to workplace violence: Yes n=115 (86.6%), No n=21 (13.4%).<br>Type of workplace violence experienced: Verbal n=115 (84.6%); Psychological n=64 (47.1%); Physical n=50 (36.8%).<br><br>*Impact of workplace violence*<br>Feelings (despair, humiliation, anger, frustration, insecurity, fear): n=77 (56.6%).<br>My motivation/performance decreased: n=76 (55.9%).<br>Saw it as part of my work: n=32 (23.5%).<br>Received psychological support: n=27 (19.9%).<br>Demanded transposition: n=19 (14.0%).<br>Perception of measures/sanctions: Insufficient n=119 (87.5%), Sufficient n=17 (12.5%)<br>Job satisfaction by workplace violence exposure: Exposed n=115: 83.76 ± 20.23 vs Not exposed n=21: 85.54 ± 17.64; p = 0.04
Hao et al., [34]<br><br>2025<br><br>China	Cross-sectional, descriptive study<br><br>N/A<br><br>Gender, age, marital status, years of experience, shifts, sleep status, self-rated health, workplace violence, work-life balance, support style, coping style	To identify different potential profiles of mental workload among emergency department nurses and analyze the influencing factors	NASA-task load index<br><br>Perceived social support scale<br><br>Simplified coping skill questionnaire<br><br>Eysenck personality questionnaire-revised, short scale for Chinese (EPQ-RSC)	*Sample characteristics*<br>Gender: Female n=228 (74.8%); Male n=77 (25.2%).<br>Age: ≤30 n=141 (46.2%); 31–40 n=136 (44.6%); >40 n=28 (9.2%).<br>Marital status: Unmarried n=110 (36.1%); Married n=191 (62.6%); Other n=4 (1.3%).<br>Years of experience: ≤5 n=100 (32.8%); 6–10 n=80 (26.2%); >10 n=125 (41.0%).<br>Monthly night shifts: None n=38 (12.5%); 1–2 n=19 (6.2%); 3–5 n=44 (14.4%); >5 n=204 (66.9%).<br>Sleep status: Normal n=141 (46.2%); Difficulty falling asleep n=73 (23.9%); Early awakening n=16 (5.2%); Intermittent n=75 (24.6%).<br>Self-rated health: Good n=177 (58.0%); Moderate n=115 (37.7%); Poor n=13 (4.3%).<br>Workplace violence (ever): Yes n=95 (31.1%); No n=210 (68.9%).<br>Work-life balance: Yes n=266 (87.2%); No n=39 (12.8%).<br>Support style: Low n=16 (5.2%); Moderate n=124 (40.7%); High n=165 (54.1%).<br>Coping style: Positive n=99 (32.5%); Negative n=209 (68.5%).

	n=305<br><br>6/8			*Latent profile analysis*<br>Classes (n, %):<br>Class 1: "Moderate mental workload": n = 163 (53.4%).<br>Class 2: "High load perception–low self-evaluation": n = 103 (33.8%).<br>Class 3: "Low load perception–high self-evaluation": n = 39 (12.8%).<br><br>*Univariate comparisons*<br>Significant differences ($p < 0.05$): Age ($\chi^2 = 3.111$, p = 0.046); Marital status ($\chi^2 = 3.874$, p = 0.022); Education ($\chi^2 = 6.237$, p = 0.002); Monthly income ($\chi^2 = 6.810$, p = 0.001); Night-shift frequency ($\chi^2 = 6.648$, p = 0.001); Support style ($\chi^2 = 4.207$, p = 0.016); Coping style ($\chi^2 = 14.107$, $p < 0.001$); Personality ($\chi^2 = 41.787$, $p < 0.001$).
Hines-Stellisch et al., [24]<br><br>2024<br><br>United States of America	Longitudinal, quasi-experimental study with one group<br><br>Individual wellness coaching (unscripted, modular), weekly 30–60 min sessions, 6–8 weeks; average 6.1 hours over 7.5 sessions.<br>Curriculum: burnout background, resilience, growth, cognitive distortions, self-care<br><br>Sex, race, years in role, role, emotional exhaustion, depersonalization,	To determine the effectiveness of a wellness coaching program for reducing burnout and turnover in emergency nurses and advanced practice providers	Maslach Burnout Inventory<br><br>Turnover Intention Scale<br><br>Custom-tailored program evaluation questions	*Sample characteristics*<br>Sex: Female n=8 (80.0%), Male n=2 (20.0%).<br>Years in role: 0–2 n=1 (10.0%); 2–5 n=2 (20.0%); 5–10 n=4 (40.0%); >10 n=3 (30.0%).<br>Role: nurse n=8 (80.0%); advance practice professional n=2 (20.0%); physician n=0 (0%).<br><br>*Pre-post primary outcomes*<br>Emotional Exhaustion: 26.1 to 20.7 (SD not reported); Cohen's d = 0.79 (large, reduction).<br>Depersonalization: 10.2 to 10.7 (SD not reported); Cohen's d = −0.18 (= no effect).<br>Personal Achievement (higher = better): 35.4 to 37.6 (SD not reported); Cohen's d = −0.35 (negative sign reflects improvement because PA is reverse-scored in burnout; small).<br>Turnover intention: 19.6 to 15.1 (SD not reported); Cohen's d = 1.37 (very large reduction).<br>P-values for pre–post change were not reported<br><br>*Program evaluation outcomes*<br>"This helped my burnout": Strongly agree n=6 (60.0%), Somewhat agree n=3 (30.0%), Neither n=1 (10.0%).<br>Would consider coaching again: Yes n=9 (90.0%), No n=1 (10.0%).<br>Sessions tailored to needs: Yes n=9 (90.0%), No n=1 (10.0%).<br>Active learning opportunities: Yes n=10 (100%), No n=0 (0%).<br>How much talking did you do? "A lot" n=6 (60.0%); "A great deal" n=4 (40.0%).

	personal achievement, turnover intention, program evaluation<br><br>n=10<br><br>5/9			
Hu et al., [78]<br><br>2022<br><br>China	Cross-sectional, descriptive study<br><br>N/A<br><br>Age, gender, marital status, years of experience in emergency departments, overtime working, organizational climate, workplace psychological violence, work engagement<br><br>n=243<br><br>6/8	To investigate the current situation of work engagement of nurses in emergency department, and to find out the impact of psychological violence on work engagement and its impact path.	Workplace Psychologically Violent Behaviors instrument (WPVB)<br><br>Nurse's Organizational Climate Scale (NOCS)<br><br>Utrecht Work Engagement Scale (UWES)	*Sample characteristics*<br>Age: 20-30 (n=104; 43.2%); 31-40 (n=100; 41.2%); 41-50 (n=33; 13.6%); >50 (n=5; 2.1%)<br>Gender: female (n=218; 89.7%); male (n=25; 10.3%)<br>Marital status: unmarried (n=63; 25.9%); married/divorced (n=180; 74.1%)<br>Years of experience in emergency departments: <5 /(n=50; 20.6%); 5-15 (127; 52.3%); 15-25 (n=52; 21.4%); >25 (n=14; 5.8%)<br>Overtime working: never (n=8; 3.3%); occasionally (n=90; 37.0%); sometimes (n=59; 24.3%); often (n=75; 39.9%); always (n=11; 4.5%)<br>Organizational climate (NOCS total): 108.6 ± 20.4. Subscales: Resource assurance 29.1 ± 6.0; Team behavior 22.0 ± 5.2; Management support 27.0 ± 5.0; Quality management 12.3 ± 2.1; Human resource management 12.2 ± 2.2; Evidence-based support 6.0 ± 1.3.<br>Workplace psychological violence (WPVB total): 63.3 ± 27.4. Subscales: Isolation at work 23.3 ± 10.4; Attacks on professional status 18.7 ± 9.1; Personality attack 16.8 ± 7.7; Direct negative behavior 4.8 ± 2.5.<br>Work engagement (UWES total): 65.8 ± 18.0. Vitality 27.5 ± 7.7; Dedication 16.8 ± 5.3; Focus 21.5 ± 6.3. High engagement: n=142 (58.4%)<br><br>*Group comparisons of outcomes*<br>Gender and Psychological violence: Female 61.7 ± 26.0 (n=218) vs Male 77.3 ± 35.1 (n=25); t=2.731, p<0.05.<br><br>*Overtime frequency and Organizational climate & engagement:*<br>Organizational climate: Never 120.3 ± 14.5 (n=8); Occasionally 112.6 ± 18.3 (n=90); Sometimes 110.0 ± 20.7 (n=59); Often 104.3 ± 20.2 (n=75); Always 89.6 ± 24.5 (n=11); F=5.159, p=0.001.<br>Vitality: F=2.669, p<0.05 (e.g., Always 21.7 ± 7.0 vs Never 29.9 ± 10.8).<br>Dedication: F=4.123, p<0.05 (Always 11.3 ± 5.0 vs Never 18.1 ± 6.8).<br>Focus: F=3.406, p<0.05 (Always 15.7 ± 7.4 vs Never 22.4 ± 8.3).

				Correlations<br>Psychological violence and Organizational climate: r = −0.448, p<0.001.<br>Psychological violence and Work engagement: Vitality r = −0.331, Dedication r = −0.256, Focus r = −0.192 (all p<0.001).<br>Organizational climate and Work engagement: Vitality r = 0.536, Dedication r = 0.489, Focus r = 0.476 (all p<0.001)
Huang et al., [32]<br><br>2024<br><br>China	Cross-sectional, descriptive study<br><br>N/A<br><br>Gender, age, marital status, years of experience, resilience, organizational support, fear of future workplace violence<br><br>n=466<br><br>5/8	To investigate the relationships among resilience scores, perceived organizational support, and workplace violence and to explore the mediating role of perceived organizational support in the relationship between resilience scores and workplace violence	Connor-Davidson Resilience Questionnaire (CD-RISC)<br><br>Perceived Organizational Support (POS) Questionnaire<br><br>Fear of future violence at work (FFVW) scale	*Sample characteristics*<br>Gender: Male n=27 (5.8%); Female n=439 (94.2%).<br>Age: ≤25 n=122 (26.2%); 26–30 n=120 (25.8%); 31–35 n=145 (31.1%); 36–40 n=48 (10.3%); >40 n=31 (6.7%).<br>Marital status: Married n=272 (58.4%); Unmarried n=194 (41.6%).<br>Years of experience: <3 n=83 (17.8%); 3–5 n=77 (16.5%); 6–10 n=133 (28.5%); 11–20 n=145 (31.1%); >20 n=28 (6.0%).<br><br>*Questionnaires' mean scores*<br>CD-RISC-10: 25.76 ± 8.41.<br>POS: 33.57 ± 11.85.<br>FFVW: 59.07 ± 16.18.<br><br>*Group differences*<br>CD-RISC-10<br>Gender: Male 20.70 ± 9.47 vs Female 26.08 ± 8.25; t = −3.256, p = 0.001.<br>Age: ≤25 23.48 ± 8.73; 26–30 25.99 ± 8.04; 31–35 25.66 ± 8.39; 36–40 30.29 ± 6.88; >40 27.35 ± 7.91; F = 6.306, p < 0.001.<br>Marital: Married 24.68 ± 8.70 vs Unmarried 27.28 ± 7.75; t = −3.396, p = 0.001.<br>Years: <3 23.19 ± 9.46; 3–5 24.90 ± 8.10; 6–10 25.35 ± 8.33; 11–20 27.66 ± 7.62; >20 27.96 ± 8.02; F = 4.687, p = 0.001.<br><br>POS<br>Gender: t = −0.668, p = 0.510 (Male 31.81 ± 14.23; Female 33.68 ± 11.70).<br>Age: F = 1.156, p = 0.329 (ns).<br>Marital: t = −1.420, p = 0.156 (ns).<br>Years of experience: F = 2.600, p = 0.036 (significant; means: <3 31.30 ± 12.61; 3–5 33.65 ± 11.77; 6–10 32.24 ± 12.02; 11–20 35.83 ± 11.11; >20 34.75 ± 11.33).<br><br>FFVW

				Gender: Male 54.74 ± 19.89 vs Female 59.33 ± 15.92; t = −1.177, p = 0.249 (ns).<br>Age: ≤25 58.99 ± 15.79; 26–30 61.49 ± 15.33; 31–35 59.74 ± 16.44; 36–40 55.33 ± 16.56; >40 52.65 ± 17.43; F = 2.632, p = 0.034.<br>Marital: Married 60.61 ± 15.98 vs Unmarried 56.91 ± 16.26; t = 2.448, p = 0.015.<br>Years of experience: F = 1.454, p = 0.215 (ns).
Jiang et al.,[25]<br><br>2024<br><br>China	Cross-sectional, descriptive study<br><br>N/A<br><br>Age, gender, marital status, depression and depressive symptoms, workplace violence, shifts, effort-reward imbalance, self-efficacy<br><br>n=111 577 (n=10 457 emergency physicians and n=101 120 general population)<br><br>8/8	To compare the risk of depressive symptoms between emergency physicians and the general population in China	Patient Health Questionnaire-9 (PHQ-9)<br><br>Effort-Reward Imbalance Scale<br><br>General Self-Efficacy Scale	*Sample characteristics*<br>Physicians<br>Age: ≤ 30 n=2600 (24.9%); 31-40 n=4977 (47.6%); ≥ 41 n=2880 (27.5%).<br>Gender: Male n=7632 (73.0%); Female n=2825 (27.0%).<br>Marital status: Unmarried/other n=1629 (15.6%); Married n=8828 (84.4%).<br>Major depression: No n=6723 (64.3%); Yes n=3734 (35.7%).<br>Severity of depressive symptoms: None n=2330 (22.3%); Mild n=4393 (42.0%); Moderate n=2027 (19.4%); Moderately severe n=1097 (10.5%); Severe n=610 (5.8%).<br><br>*Within-physicians comparisons by depression status*<br>Workplace violence (ever): Major depression: Yes n=3,493 (93.6%); No n=241 (6.5%). Without major depression: Yes n=5,111 (76.0%); No n=1,612 (24.0%); χ^2=505.586, p<0.001.<br><br>Night shifts (per month): ≤5: 464 (12.4%) vs 1,569 (23.3%); 6–10: 2,017 (54.0%) vs 3,616 (53.8%); ≥11: 1,253 (33.6%) vs 1,538 (22.9%); χ^2=249.630, p<0.001 (order: major depression vs without).<br><br>Effort–reward imbalance: Yes n=3,477 (93.1%) vs 4,720 (70.2%); No n=257 (6.9%) vs 2,003 (29.8%); χ^2=743.793, p<0.001.<br><br>Self-efficacy: Major depression 23.0 ± 6.4 vs without 26.8 ± 5.9; p<0.001.
Jiang et al.,[53]<br><br>2023<br><br>China	Cross-sectional, descriptive study<br><br>N/A<br><br>Age, gender, job seniority, shift, workplace violence, self-rated	To examine relationships between turnover intention and demographic characteristics, job-related factors, depressive symptoms, and	Custom-tailored self-rated health status question<br><br>Patient Health Questionnaire-9 (PHQ-9)	*Sample characteristics*<br>Age = 29.9 ± 6.8 years.<br>Gender (n=17,582): Male n=1,803 (10.3%), Female n=15,779 (89.8%).<br>Job seniority: <1 year n=2,540 (14.5%); 1–10 years n=12,356 (70.3%); ≥11 years n=2,686 (15.3%).<br>Night shifts per month: ≤5 n=5,417 (30.8%); 6–10 n=7,719 (43.9%); ≥11 n=4,446 (25.3%).<br>Workplace violence in the past year: Yes n=12,353 (70.3%), No n=5,229 (29.7%).<br>Self-rated health: Good n=3,960 (22.5%); Fair n=9,325 (53.0%); Poor n=4,297 (24.4%).<br>Turnover intention: 10.18 ± 2.96

	health, turnover intention, depression, organizational commitment<br><br>n= 17 582<br><br>7/8	organizational commitment among emergency department nurses	Organizational Commitment Questionnaire<br><br>Turnover Intention Scale	Depressive symptoms: 7.53 ± 4.98<br>Organizational commitment: 20.10 ± 4.05<br><br>*Sample characteristics related to turnover intention*<br>Gender (n=17,582): Male n=1,803 (10.3%); 10.41 ± 3.38 vs Female n=15,779 (89.8%); 10.15 ± 2.91; t=3.14, p=0.0017.<br><br>Self-rated health: Good n=3,960 (22.5%); 9.03 ± 2.68; Fair n=9,325 (53.0%); 10.10 ± 2.75; Poor n=4,297 (24.4%); 11.41 ± 3.19; F=726.96, p<0.0001.<br><br>Job seniority: <1 y n=2,540 (14.5%); 9.83 ± 2.86; 1–10 y n=12,356 (70.3%); 10.29 ± 2.99; ≥11 y n=2,686 (15.3%); 9.99 ± 2.90; F=32.61, p<0.0001.<br><br>Night shifts/month: ≤5 n=5,417 (30.8%); 9.80 ± 2.78; 6–10 n=7,719 (43.9%); 10.16 ± 2.96; ≥11 n=4,446 (25.3%); 10.67 ± 3.12; F=107.00, p<0.0001.<br><br>Workplace violence in past year: Yes n=12,353 (70.3%); 10.52 ± 3.00 vs No n=5,229 (29.7%); 9.38 ± 2.71; t=−24.82, p<0.0001.<br><br>*Correlations*<br>Depressive symptoms and turnover intention: r = 0.42, p<0.0001.<br>Organizational commitment and turnover intention: r = −0.68, p<0.0001.<br>Depressive symptoms and organizational commitment: r = −0.45, p<0.0001.
Kim et al., [30]<br><br>2022<br><br>South Korea	Cross-sectional, descriptive study<br><br>N/A<br><br>Gender, age, marital status, years of experience, years of experience in emergency departments, job satisfaction, types and frequency of	To examine the relationship between violence experience from patients or their caregivers, resilience, and nursing performance among emergency room nurses in Korea	Violence experience tool<br><br>Korean version of the Connor Davision Resilience Scale (KCDRISC)<br><br>Nursing performance tool	*Sample characteristics*<br>Gender: Male n=13 (10.0%); Female n=117 (90.0%).<br>Age: <30 n=40 (30.8%); 30–34 n=59 (45.4%); ≥35 n=31 (23.8%).<br>Marital status: Married n=30 (23.1%); Single n=100 (76.9%).<br>Years of experience: <3 n=26 (20.0%); 3–5 n=48 (36.9%); 6–7 n=25 (19.2%); 8–10 n=14 (10.8%); >10 n=17 (13.1%).<br>Years of experience in emergency departments: <3 n=89 (68.5%); 3–5 n=25 (19.2%); >5 n=16 (12.3%).<br>Job satisfaction: Bad n=40 (30.8%); Moderate n=63 (48.5%); Good n=27 (20.7%).<br><br>*Types and frequency of violence*<br>Verbal (per week): “Cursing” n=72 (55.4%) 1 time; “Talking down” n=51 (39.2%) 1 time; “Screaming” n=59 (45.4%) 1 time; “Threatening” n=57 (43.8%) 1 time.

violence, resilience, performance

n= 130

5/8

Physical threats (per month): “Taking a stance to hit” n=55 (42.3%) 1 time; “Posture to throw things” n=50 (38.5%) 1 time; “Kicking hospital items” n=63 (48.5%) 1 time; “Wandering around with anger” n=47 (36.2%) 1 time; “Making a fierce look” n=40 (30.8%) 1 time and n=40 (30.8%) 2 times.

Physical violence (per year): “Throwing an object at me” n=74 (56.9%) none; “Striking or kicking me” n=70 (53.8%) none; “Pushing me” n=41 (31.5%) 2 times; “Catching a part of my body” n=43 (33.1%) 1 time; “Scratching” n=79 (60.8%) none; “Biting” n=93 (71.5%) none; “Spitting at me” n=80 (61.5%) none.

Scales’ outcomes
Violence experience total: 24.26 ± 12.79; Verbal 8.15 ± 3.98; Physical threat 8.98 ± 4.93; Physical violence 7.13 ± 5.85.
Resilience (K-CD-RISC): 81.64 ± 15.26.
Nursing performance: 62.08 ± 10.26.

Group differences
Violence experience
Age: <30 21.33 ± 10.22, 30–34 20.83 ± 10.19, ≥35 14.23 ± 8.73; F=5.633, p=0.005; post-hoc: younger groups > ≥35.
Years of experience: <3 21.00 ± 13.42; 3–5 20.83 ± 9.26; 6–7 21.08 ± 8.12; 8–10 13.07 ± 10.62; >10 15.71 ± 7.43; F=2.580, p=0.041.
Years of experience in emergency departments: <3 21.34 ± 10.70; 3–5 15.68 ± 7.68; >5 14.50 ± 7.91; F=5.439, p=0.005.
Job satisfaction: Bad 23.13 ± 8.64; Moderate 18.62 ± 10.98; Good 15.74 ± 9.07; F=4.839, p=0.009 (Bad > Good).

Resilience
Marital: Married 87.90 ± 16.20 vs Single 79.80 ± 14.40; t=–2.605, p=0.010.
Years of experience: <3 81.90 ± 15.90; 3–5 83.10 ± 13.20; 6–7 79.50 ± 15.90; 8–10 68.70 ± 11.70; >10 90.60 ± 14.40; F=4.772, p=0.001.
Job satisfaction: Bad 77.10 ± 11.10; Moderate 82.20 ± 15.00; Good 87.00 ± 19.20; F=3.693, p=0.028.

Nursing performance
Gender: Male 54.06 ± 11.22 vs Female 62.90 ± 9.86; t=–3.061, p=0.003.

				Age: <30 65.45 ± 9.52; 30–34 59.50 ± 9.69; ≥35 62.73 ± 11.22; F=4.212, p=0.017 (post-hoc: <30 > 30–34).<br>Marital: Married 67.49 ± 9.18 vs Single 60.52 ± 10.03; t=−3.409, p=0.001.<br>Years of experience: <3 61.20 ± 10.88; 3–5 62.39 ± 9.86; 6–7 60.69 ± 9.86; 8–10 57.29 ± 9.52; >10 68.51 ± 9.35; F=2.696, p=0.034 (8–10 < >10).<br>Years of experience in emergency departments: <3 60.52 ± 10.37; 3–5 64.26 ± 7.99; >5 67.15 ± 10.71; F=3.656, p=0.029 (<3 < >5).<br>Job satisfaction: Bad 58.48 ± 10.37; Moderate 62.39 ± 9.18; Good 66.81 ± 10.71; F=5.864, p=0.004 (Bad < Good).<br><br>*Correlations*<br>Resilience and Nursing performance: r = 0.610, p<0.001.<br>Violence experience and Resilience: r = 0.152, p=0.084 (ns).<br>Violence experience and Nursing performance: r = 0.107, p=0.226 (ns).
Kousha et al.,[37]<br><br>2022<br><br>Iran	Randomized controlled trial<br><br>Intervention: Five 2-hour sessions over 3 weeks on different workdays/shifts; content included definitions of incivility, 10 common incivilities, appropriate response methods, and role-plays; delivered by an emergency nurse and a mental health nurse<br><br>Control: Written information about	To investigate the effectiveness of educational intervention and cognitive rehearsal on perceived incivility among emergency nurses	Incivility Scale (measured at baseline and 1-month post-intervention)	*Sample characteristics*<br>Sex: not reported in counts/percentages; narrative states “most of the participants in this study were women”<br>Age: Intervention 31.6 ± 5.5; Control 29.1 ± 4.4. t = 2.20; p = .31 (ns).<br>Years of experience: Intervention 79.0 ± 68.4; Control 59.5 ± 46.2; t = 1.50; p = 0.14 (ns)<br>Months of experience in emergency departments: Intervention 63.2 ± 60.5; Control 45.6 ± 41.5; t = 1.52; p = 0.13 (ns).<br>Work hours/week: Intervention 53.8 ± 13.6; Control 55.8 ± 12.1; t = 0.62; p = 0.54 (ns).<br><br>*Primary outcomes*<br>General incivility:<br>Intervention: 2.4 ± 0.8 to 2.4 ± 0.7<br>Control: 2.3 ± 0.8 to 2.2 ± 0.8<br>Group comparison p = 0.01.<br><br>Nurses’ incivility towards other nurses:<br>Intervention: 2.2 ± 1.0 to 2.3 ± 0.8<br>Control: 2.4 ± 0.9 to 2.2 ± 0.7<br>Group comparison p = 0.06 (ns).<br><br>Supervisor incivility:<br>Intervention: 2.0 ± 0.8 to 2.3 ± 0.7<br>Control: 2.3 ± 0.7 to 2.2 ± 0.8

	what incivility is and how to deal with it<br><br>Age, years of experience, months of experience in emergency departments, work hours/week, overall incivility, agents' incivility<br><br>n=80 (n=40 in each of the two hospitals)<br><br>5/13			Group comparison p = 0.01.<br><br>Physician incivility:<br>Intervention: 2.6 ± 0.8 to 2.4 ± 0.9<br>Control: 2.9 ± 0.9 to 2.6 ± 0.8<br>Group comparison p = 0.12 (ns).<br><br>Patient/visitor incivility:<br>Intervention: 2.9 ± 0.9 to 2.7 ± 0.9<br>Control: 2.7 ± 0.9 to 2.5 ± 0.9<br>Group comparison p = 0.17 (ns).<br><br>Overall incivility (total):<br>Intervention: 2.4 ± 0.7 to 2.4 ± 0.7<br>Control: 2.5 ± 0.7 to 2.4 ± 0.7<br>Group comparison p = 0.01.
Labrague, [38]<br><br>2024<br><br>United States of America	Cross-sectional, descriptive study<br><br>N/A<br><br>Age, gender, marital status, years of experience, job seniority, work satisfaction, psychological distress, work-family conflict, toxic leadership<br><br>n= 283<br><br>6/8	To examine the mediating effects of work-family conflict on the relationship between toxic leadership behaviors and psychological distress and work satisfaction among emergency nurses	Toxic Leadership Behaviors of Nurse Managers Scale (ToxBH-NM)<br><br>Work-Family Conflict Scale (WFC)<br><br>Job Stress Scale (JSS)<br><br>Job Satisfaction Index (JSI)	*Sample characteristics*<br>Age: 29.79 ± 6.04 years.<br>Gender: Male n=131 (46.3%); Female n=152 (53.7%).<br>Marital status: Married n=141 (49.8%); Unmarried n=142 (50.2%).<br>Years of experience: 6.96 ± 5.63.<br>Job seniority (years): 3.53 ± 3.96.<br><br>*Descriptive outcomes*<br>Work satisfaction (JSI): 3.4223 ± 0.6286.<br>Psychological distress (JSS): 2.9514 ± 0.4457.<br>Work-family conflict: 3.2163 ± 0.6419.<br>Toxic leadership (ToxBH-NM): 1.4387 ± 0.5486.<br><br>*Correlations*<br>Work satisfaction and Psychological distress: r = −0.126, $p<0.05$.<br>Work satisfaction and WFC: r = −0.300, $p<0.01$.<br>Psychological distress and WFC: r = 0.197, $p<0.05$.<br>Toxic leadership and Work satisfaction: r = −0.327, $p<0.01$.<br>Toxic leadership and Psychological distress: r = 0.143, $p<0.05$.

				Toxic leadership and WFC: r = 0.149, p<0.05.
Lee et al., [26]<br><br>2021<br><br>United States of America	Cross-sectional, descriptive study<br><br>N/A<br><br>Age, sex, years of experience, shift, emotional exhaustion, depersonalization, personal accomplishment, burnout, turnover intention<br><br>n=77<br><br>8/8	To examine the relationship among orientation, burnout (emotional exhaustion, depersonalization, and low sense of personal accomplishment), and intent to leave	Turnover Intention Scale<br><br>Maslach Burnout Inventory<br><br>Custom-tailored emotional exhaustion questionnaire<br><br>Custom-tailored depersonalization questionnaire<br><br>Custom-tailored personal accomplishment questionnaire	*Sample characteristics*<br>Age groups: 20–30 n=15 (19.5%); 31–40 n=25 (32.5%); 41–50 n=19 (24.7%); 51–60 n=14 (18.2%); 61–70 n=4 (5.2%).<br>Sex: Male n=11 (14.3%); Female n=65 (85.7%).<br>Years of experience: <5 n=29 (37.7%); 5–10 n=18 (23.4%); 11–15 n=7 (9.1%); 16–20 n=11 (14.3%); 21–25 n=4 (5.2%); >25 n=8 (10.4%).<br>Shift: Day 7a–7p n=36 (46.8%); Night 7p–7a n=16 (20.8%); Mid 9a–9p n=3 (3.9%); Mid 11a–11p n=11 (14.3%); Mid 3p–3a n=2 (2.6%); Day 7a–3p n=6 (7.8%); Night 11p–7a n=3 (3.9%).<br><br>*Descriptive outcomes*<br>Emotional exhaustion: 36.77 ± 11.01.<br>Depersonalization: 16.99 ± 6.17.<br>Personal accomplishment: 44.56 ± 7.45.<br>Burnout: 98.25 ± 15.20.<br>Turnover intention: 17.91 ± 3.82.<br><br>*Correlations*<br>Age and Depersonalization: r = −0.32, p = 0.01<br>Experience (≥5 years) and Turnover intention: r = 0.23, p = 0.046<br>Burnout (total) and Turnover intention: r = 0.43, p < 0.001.<br>Emotional exhaustion and Turnover intention: r = 0.71, p < 0.001.<br>Depersonalization and Turnover intention: r = 0.36, p = 0.002.<br>Personal accomplishment and Turnover intention: r = −0.46, p < 0.001.
Lei et al., [27]<br><br>2022<br><br>China	Cross-sectional, descriptive study<br><br>N/A<br><br>Age, gender, marital status, divorced, work tenure, shift work, work stress, self-perceived health, sleep quality	To find out the prevalence, characteristics, and predictors of workplace violence (WPV) against Chinese emergency department nurses	Workplace Violence Scale	*Sample characteristics*<br>Age = 30.83 ± 6.42 years (range 18–65).<br>Gender: Male n=2,133 (10.59%); Female n=18,003 (89.41%).<br>Marital status: Unmarried n=6,318 (31.38%); Married n=13,328 (66.19%); Divorced n=461 (2.29%); Widowed n=29 (0.14%).<br>Work tenure: <10 y n=15,138 (75.18%); ≥10 y n=4,998 (24.82%).<br>Shift work: Yes n=17,727 (88.04%); No n=2,409 (11.96%).<br>Work stress: Low n=1,143 (5.68%); Medium n=5,051 (25.08%); High n=13,942 (69.24%).<br>Self-perceived health: Good n=7,165 (35.58%), General n=10,201 (50.66%), Bad n=2,770 (13.76%)

	n=20136<br><br>7/8			Sleep quality: Good n=2,536 (12.59%), General n=10,394 (51.62%), Bad n=7,206 (35.79%).<br><br>*Prevalence of workplace violence*<br>Any workplace violence: n=15,985 (79.39%).<br>Nonphysical violence (verbal abuse/threat/verbal sexual harassment): n=15,782 (78.38%).<br>Physical violence (physical assault/physical sexual assault): n=7,984 (39.65%)<br>Verbal abuse: n=15,147 (75.22%) — once 3,383 (16.80%), 2–3 times 3,952 (19.63%), >3 times 7,812 (38.80%).<br>Threat: n=10,372 (51.51%) — once 4,021 (19.97%), 2–3 2,766 (13.74%), >3 3,585 (17.80%).<br>Physical assault: n=7,531 (37.40%) — once 3,688 (18.32%), 2–3 2,025 (10.06%), >3 1,818 (9.03%).<br>Verbal sexual harassment: n=4,995 (24.81%) — once 2,037 (10.12%), 2–3 1,142 (5.67%), >3 1,816 (9.02%).<br>Physical sexual assault: n=2,455 (12.19%) — once 1,274 (6.33%), 2–3 546 (2.71%), >3 635 (3.15%)
Li et al., [63]<br><br>2020<br><br>China	Cross-sectional, descriptive study<br><br>N/A<br><br>Gender, age, marital status, turnover intention, job satisfaction, organizational commitment, workplace violence<br><br>n=385<br><br>7/8	To describe turnover intention of emergency nurses and clarify the effects of organizational commitment, job satisfaction and workplace violence on turnover intention	Turnover Intention Tool<br><br>McCloskey/Muller Satisfaction Scale<br><br>Organizational Commitment Scale for Chinese population	*Sample characteristics*<br>Gender: Female n=363 (94.3%), Male n=22 (5.7%).<br>Age: 29.49 ± 5.99 years.<br>Marital status: Unmarried n=166 (43.1%), Married n=219 (56.9%).<br>Job seniority: Hospital 9.04 years (median, IQR 6.06); emergency departments 7.60 years (median, IQR 5.08).<br><br>*Descriptive outcomes*<br>Turnover intention (overall): 2.75 ± 0.58.<br>Turnover intention levels: High n=242 (62.9%), Very high n=105 (27.3%), Low n=30 (7.8%), Very low n=8 (2.1%). (High+Very high = n=347 (90.2%)).<br>Turnover intention subscales:<br>Possibility of quitting the current job 3.01 ± 0.60<br>Motivation to look for another job 2.53 ± 0.70<br><br>Job satisfaction: 2.48 ± 0.49. Highest subscale: co-workers 3.69 ± 0.50; lowest: extrinsic rewards 1.93 ± 0.68.

				Organizational commitment: 2.21 ± 0.40. Highest dimension: normative 2.44 ± 0.53; lowest: opportunity 1.96 ± 0.56.<br><br>Workplace violence in the past twelve months: High frequency 1.8% (n=7); Moderate 49.9% (n=192); Low 38.2% (n=147); None 10.1% (n=39).<br>Any workplace violence: 89.9% (n=346).<br><br>*Correlations*<br>Organizational commitment and workplace violence: r = 0.129* (p < 0.05).<br>Organizational commitment and job satisfaction: r = 0.440 ** (p < 0.01).<br>Organizational commitment and turnover intention: r = −0.491 ** (p < 0.01).<br>Workplace violence and job satisfaction: r = −0.193 ** (p < 0.01).<br>Workplace violence and turnover intention: r = 0.145 ** (p < 0.01).<br>Job satisfaction and turnover intention: r = −0.483 ** (p < 0.01).
Liao et al., [57]<br><br>2025<br><br>China	Cross-sectional, descriptive study<br><br>N/A<br><br>Age, gender, years of experience, years of experience in emergency departments, workplace violence, resilience, positive and negative coping, perceived organizational support.<br><br>n=670<br><br>8/8	To examine the potential mediation effects of coping styles between perceived organizational support and resilience in emergency nurses exposed to workplace violence	Nurses' Perceived Organizational Support (POS) Scale<br><br>Trait Coping Style Questionnaire (TCSQ)<br><br>Connor–Davidson Resilience Scale-10(CD-RISC-10	*Sample characteristics*<br>Age = 30.94 ± 6.13 years.<br>Gender: Female n=563 (84.0%); Male n=107 (16.0%)<br>Years of experience: 8.94 ± 6.54<br>Years of experience in emergency departments: 6.70 ± 5.57.<br>Workplace violence type in the last 3 months: Verbal abuse only n=541 (80.7%); Physical attacks only n=90 (13.4%); Both n=39 (5.8%)<br><br>*Descriptive outcomes*<br>Resilience (CD-RISC-10): 27.73 ± 7.51 (0–40).<br>Positive coping: 34.07 ± 5.79 (10–50).<br>Negative coping: 27.13 ± 6.03 (10–50).<br>POS: 57.73 ± 10.61 (15–75).<br><br>*Resilience outcomes per group*<br>By marital status: Has spouse 27.21 ± 7.92 (n=403) vs No spouse 28.52 ± 6.80 (n=267); t = −2.215, p < 0.01.<br>By WPV type: Verbal only 28.12 ± 7.45 (n=541); Physical only 26.16 ± 7.50 (n=90); Both 26.05 ± 7.96 (n=39); F = 3.692, p < 0.01.<br><br>*Correlations (p < 0.01 for all)*<br>POS and Resilience: r = 0.478.<br>Positive coping and Resilience: r = 0.427.

				Negative coping and Resilience: r = −0.405.<br>POS and Positive coping: r = 0.367<br>POS and Negative coping: r = −0.379<br>Positive and Negative coping: r = −0.264.
Lovell et al.,[44]<br><br>2022<br><br>Barbados, The Bahamas, Trinidad & Tobago	Cross-sectional, descriptive study<br><br>N/A<br><br>Gender, age, marital status, working hours/week, depressive episodes, self-rated health, burnout, resilience<br><br>n=111<br><br>5/8	To explore burnout and resilience amongst physicians working in Caribbean emergency departments	Maslach Burnout Inventory (MBI)<br><br>Resilience Scale-14 (RS-14)	*Sample characteristics*<br>Gender: Male n=49 (44%), Female n=62 (56%).<br>Age groups: 20–29 n=19 (18%), 30–39 n=53 (50%), 40–49 n=27 (25%), 50+ n=8 (6%).<br>Marital status: Married n=55 (49%), Divorced n=4 (4%), Never married/single n=52 (47%).<br>Working hours/week: <40 h n=30 (27.0%), >40 h n=77 (69.4%).<br><br>Felt depressed in past 2 weeks: Never n=54 (48.2%), Sometimes n=44 (39.3%), Frequently n=8 (7.1%), All the time n=3 (2.7%).<br>Self-rated health: Excellent n=24 (21.4%), Very good n=36 (32.1%), Good n=35 (31.3%), Fair n=12 (10.7%), Poor n=3 (2.7%).<br><br>*Descriptive outcomes*<br>MBI subscale means (±SD): emotional exhaustion 31.1 ± 11.9 (range 10–58 of 63); depersonalization 12.5 ± 6.5 (5–31 of 35); personal accomplishment 44.3 ± 7.4 (21–55 of 56).<br>Emotional exhaustion: low n=12 (11.3%), medium n=28 (26.3%), high n=66 (62.3%).<br>Depersonalization: low n=19 (17.1%), medium n=48 (43.2%), high n=44 (39.6%)<br>Personal accomplishment: low n=4 (3.7%), medium n=16 (15.9%), high n=86 (80.4%) (n=106).<br><br>Resilience (RS-14): 81.4 ± 13.1 (range 26–98 of 98).<br>Resilience by category: very low n=3 (2.8%), low n=8 (7.5%), low-end n=12 (11.2%), moderate n=21 (19.6%), moderately-high n=37 (34.6%), high n=26 (24.3%)<br><br>*Group comparisons*<br>Burnout by weekly working hours<br>Emotional exhaustion: 29.3 ± 12.3 vs 32.2 ± 12.0, p=0.29.<br>Depersonalization: 10.5 ± 6.6 vs 13.1 ± 6.6, p=0.046.<br>Personal accomplishment: 44.4 ± 7.8 vs 44.4 ± 7.3, p=0.99<br>There were not significant differences among burnout subscales scores and gender, age or marital status

				Associations<br>Lower resilience in those recently depressed (p<0.0001)<br>Higher emotional exhaustion in those recently depressed (p=0.004), and who used sleeping-aids(p=0.028).<br>Higher depersonalization in those recently depressed (p=0.049) and who used sleeping-aids (p=0.034).<br>Lower personal accomplishment with poorer self-rated health (p=0.01)
Luo et al., [58]<br><br>2024<br><br>China	Cross-sectional, descriptive study<br><br>N/A<br><br>Age, gender, shift, workplace violence witnessed, workplace violence experienced, burnout<br><br>n=1173 (Physicians n=314, 26.8%; Nurses n=859, 73.2%)<br><br>7/8	To understand the status of occupational burnout among emergency department healthcare workers, analyze its influencing factors, and provide references for preventing burnout	Maslach Burnout Inventory (MBI)	*Sample characteristics*<br>Age: 34.15 ± 8.55 years.<br>Age groups: 20–30 n=401 (34.2%), 31–40 n=508 (43.3%), 41–50 n=210 (17.9%), >50 n=54 (4.6%).<br>Gender: Male n=316 (26.9%), Female n=857 (73.1%).<br>Marital status: Married n=858 (73.1%), Unmarried n=277 (23.6%), Divorced/other n=38 (3.2%).<br>Night shifts per month: 0 n=225 (19.2%), 1–3 n=134 (11.4%), 4–6 n=266 (22.7%), 7–9 n=183 (15.6%), 10–12 n=266 (22.7%), ≥13 n=99 (8.4%).<br>Workplace violence (WPV) witnessed at work: Never n=318 (27.1%), <2 weeks n=123 (10.5%), <6 months n=178 (15.2%), <1 year n=215 (18.3%), ≥1 year n=339 (28.9%).<br>WPV personally experienced: Never n=529 (45.1%), <2 weeks n=44 (3.8%), <6 months n=78 (6.6%), <1 year n=134 (11.4%), ≥1 year n=388 (33.1%).<br><br>*Descriptive outcomes*<br>MBI overall: 2.23 ± 0.87<br>Emotional exhaustion: 2.33 ± 0.31,<br>Depersonalization: 1.88 ± 0.28<br>Personal accomplishment: 3.20 ± 0.39<br><br>*Burnout prevalence and levels*<br>Any burnout (composite ≥1.5): n=946 (80.65%).<br>Mild–moderate: 73.57% n=863.<br>Severe: 7.08% n=83.<br>Subscales (moderate–severe levels): emotional exhaustion 68.46% n=803; depersonalization 53.28% n=625; personal accomplishment 27.62% n=324.<br><br>*Group comparisons*<br>Burnout in physicians vs nurses<br>MBI: 2.14 ± 0.86 vs 2.27 ± 0.88, F=4.52, p=0.03.

Emotional exhaustion: 2.18 ± 1.14 vs 2.22 ± 1.21, F=0.25, p=0.62.
Depersonalization: 1.61 ± 1.04 vs 1.69 ± 1.16, F=1.12, p=0.29.
Personal accomplishment: 2.64 ± 1.42 vs 2.91 ± 1.43, F=8.66, p<0.01.

Burnout by years of experience (<3 / 4–9 / 10–19 / ≥20 years)
MBI: 2.28 ± 0.76 / 2.39 ± 0.94 / 2.23 ± 0.84 / 1.80 ± 0.90, F=12.82, p<0.01.
Emotional exhaustion: 2.03 ± 1.07 / 2.41 ± 1.30 / 2.31 ± 1.23 / 1.78 ± 1.12, F=8.92, p<0.01.
Depersonalization: 1.56 ± 1.03 / 1.83 ± 1.28 / 1.69 ± 1.13 / 1.36 ± 1.01, F=5.08, p<0.01.
Personal accomplishment: 3.34 ± 1.39 / 3.00 ± 1.39 / 2.67 ± 1.36 / 2.28 ± 1.51, F=14.86, p<0.01.

Burnout by age (20–30 / 31–40 / 41–50 / >50 years):
MBI: 2.37 ± 0.86 / 2.30 ± 0.84 / 1.96 ± 0.89 / 1.72 ± 0.83, F=17.78, p<0.01.
Emotional exhaustion: 2.23 ± 1.21 / 2.33 ± 1.19 / 1.99 ± 1.15 / 1.78 ± 1.13, F=6.44, p<0.01.
Depersonalization: 1.73 ± 1.18 / 1.72 ± 1.14 / 1.50 ± 1.06 / 1.28 ± 0.89, F=4.33, p=0.01.
Personal accomplishment: 3.19 ± 1.47 / 2.83 ± 1.32 / 2.38 ± 1.43 / 2.06 ± 1.38, F=21.25, p<0.01.

Burnout by marital status (Married / Unmarried / Divorced/other):
MBI: 2.16 ± 0.87 / 2.48 ± 0.83 / 2.22 ± 0.85, F=9.91, p<0.01.
Emotional exhaustion: 2.17 ± 1.16 / 2.32 ± 1.28 / 2.35 ± 1.21, F=1.95, p=0.14.
Depersonalization: 1.61 ± 1.10 / 1.82 ± 1.20 / 1.74 ± 1.17, F=3.77, p=0.02.
Personal accomplishment: 2.68 ± 1.41 / 3.35 ± 1.37 / 2.55 ± 1.49, F=24.78, p<0.01.

Burnout by night shifts per month (0 / 1–3 / 4–6 / 7–9 / 10–12 / ≥13):
MBI: 1.87 ± 0.83 / 2.14 ± 0.84 / 2.25 ± 0.84 / 2.34 ± 0.83 / 2.44 ± 0.84 / 2.39 ± 0.99, F=12.67, p<0.01.
Emotional exhaustion: 1.79 ± 1.06 / 2.07 ± 1.31 / 2.14 ± 1.18 / 2.31 ± 1.20 / 2.53 ± 1.23 / 2.53 ± 1.33, F=12.23, p<0.01.
Depersonalization: 1.39 ± 0.98 / 1.53 ± 1.08 / 1.66 ± 1.12 / 1.74 ± 1.09 / 1.83 ± 1.19 / 1.88 ± 1.33, F=5.20, p<0.01.
Personal accomplishment: 2.48 ± 1.47 / 2.86 ± 1.43 / 2.99 ± 1.47 / 2.96 ± 1.41 / 2.93 ± 1.31 / 2.73 ± 1.47, F=4.12, p<0.01.

Burnout by WPV witnessed (Never / <2 weeks / <6 months / <1 year / ≥1 year):
MBI: 2.06 ± 0.84 / 2.57 ± 0.88 / 2.34 ± 0.78 / 2.38 ± 0.85 / 2.13 ± 0.91, F=11.29, p<0.01.

				Emotional exhaustion: 1.81 ± 1.15 / 2.75 ± 1.33 / 2.39 ± 1.05 / 2.37 ± 1.14 / 2.20 ± 1.18, F=18.02, p<0.01.<br>Depersonalization: 1.35 ± 1.05 / 2.16 ± 1.32 / 1.81 ± 0.98 / 1.80 ± 1.13 / 1.61 ± 1.12, F=14.37, p<0.01.<br>Personal accomplishment: 3.11 ± 1.53 / 2.74 ± 1.40 / 2.80 ± 1.22 / 2.97 ± 1.33 / 2.56 ± 1.45, F=6.77, p<0.01.<br><br>Burnout by WPV personally experienced (Never / <2 weeks / <6 months / <1 year / ≥1 year):<br>MBI: 2.12 ± 0.84 / 2.71 ± 0.75 / 2.53 ± 0.89 / 2.46 ± 0.81 / 2.20 ± 0.90, F=10.42, p<0.01.<br>Emotional exhaustion: 1.99 ± 1.15 / 2.87 ± 1.35 / 2.56 ± 1.02 / 2.53 ± 1.17 / 2.26 ± 1.21, F=12.70, p<0.01.<br>Depersonalization: 1.46 ± 1.04 / 2.33 ± 1.45 / 2.15 ± 1.17 / 1.95 ± 1.05 / 1.67 ± 1.15, F=14.52, p<0.01.<br>Personal accomplishment: 2.97 ± 1.49 / 2.86 ± 1.22 / 2.86 ± 1.26 / 2.89 ± 1.30 / 2.64 ± 1.42, F=3.03, p=0.02.
Ma et al., [33]<br><br>2022<br><br>China	Cross-sectional, descriptive study<br><br>N/A<br><br>Gender, age, marital status, years of experience in emergency departments, weekly work hours, monthly night shifts, workplace violence in the last year, work stress, perceived organizational support, burnout, job satisfaction, turnover intention	To investigate the turnover intention of emergency nurses in China, its associated factors,, and the relationship between work stress, job burnout, perceived organization support, job satisfaction and turnover intention	Work Stress Scale<br><br>Survey of Perceived Organizational Support (POS)<br><br>Maslach Burnout Inventory (MBI)<br><br>Job Satisfaction Scale<br><br>Turnover Intention Scale (TI)	*Sample characteristics*<br>Gender (N=522): Male n=33 (6.32%); Female n=489 (93.68%).<br>Age: <30 n=257 (49.23%); 30–39 n=201 (38.51%); >39 n=64 (12.26%).<br>Marital status: Married n=375 (71.84%); Single n=147 (28.16%).<br>Years of experience in emergency departments: <5 n=202 (38.70%); 5–10 n=232 (44.44%); >10 n=88 (16.86%).<br>Weekly work hours: ≤40 n=258 (49.43%); 41–60 n=185 (35.44%); >60 n=79 (15.13%).<br>Monthly night shifts: ≤5 n=179 (34.29%); 6–10 n=191 (36.59%); >10 n=152 (29.12%).<br>Workplace violence in past year (WPV): Yes n=320 (61.30%); No n=202 (38.70%).<br><br>*Descriptive outcomes*<br>Work stress: 3.05 ± 0.69 (1–4).<br>POS: 4.25 ± 1.01 (1–7).<br>MBI (overall): 1.98 ± 0.96 (0–6).<br>Job satisfaction: 4.39 ± 0.91 (1–7).<br>TI (overall): 2.38 ± 0.57 (1–4).<br>Possibility of resigning: 2.14 ± 0.74.<br>Motivation to seek other jobs: 2.17 ± 0.79.<br>Possibility of getting an external job: 2.83 ± 0.55.<br>High turnover intention (overall mean > 2.5): 40.61% n=212/522

	n=522<br><br>8/8			*Group differences in turnover intention*<br>Gender: Male 2.46 ± 0.68 vs Female 2.38 ± 0.66; F = 0.75, p not significant (exact p not reported).<br>Age: <30 2.47 ± 0.55; 30–39 2.39 ± 0.55; >39 2.00 ± 0.56; F = 18.83, p < 0.01.<br>Marital status: Married 2.34 ± 0.58 vs Single 2.49 ± 0.54; F = 7.39, p < 0.01.<br>Years of experience in emergency departments: <5 2.46 ± 0.55; 5–10 2.38 ± 0.58; >10 2.21 ± 0.57; F = 5.64, p < 0.01.<br>Exposure to WPV in the past year: Yes 2.46 ± 0.56 vs No 2.26 ± 0.57; F = 15.87, p < 0.01.<br><br>*Correlations*<br>TI with Work stress r = 0.189; POS r = −0.353; Job burnout r = 0.391; Job satisfaction r = −0.486.<br>Inter-variable: Work stress and POS r = −0.309; Work stress and Job burnout r = 0.453; Work stress and Job satisfaction r = −0.336; POS and Job burnout r = −0.510; POS and Job satisfaction r = 0.652; Job burnout and Job satisfaction r = −0.528.
Meng et al., [45]<br><br>2023<br><br>China	Cross-sectional, descriptive study<br><br>N/A<br><br>Gender, age, marital status, years of experience in emergency departments, shift, patients served per day, perceived emergency personnel shortage, self-reported health, self-reported sleep quality<br><br>n=10457<br><br>8/8	To investigate the occurrence and correlated factors of physical and verbal violence among emergency physicians in China.	Custom-tailored workplace violence-related questions<br><br>Positive and Negative Affect Scale (PANAS)<br><br>General Self-Efficacy Scale (GSES)	*Sample characteristics*<br>Gender (N=10,457): Male n=7,632 (72.98%), Female n=2,825 (27.01%).<br>Age: <30 n=1,925 (18.41%), 30–39 n=5,191 (49.64%), 40–49 n=2,668 (25.52%), ≥50 n=673 (6.43%).<br>Marital status: Unmarried n=1,395 (13.34%), Married n=8,828 (84.42%), Divorced/separated/widowed n=234 (2.24%).<br>Years of experience in emergency departments: ≤1 n=1,448 (13.85%); 1–5 n=3,965 (37.92%); >5 n=5,044 (48.23%).<br>Night shifts/month: ≤5 n=2,033 (19.44%); 6–10 n=5,633 (53.87%); >10 n=2,791 (26.69%).<br>Patients served/day: ≤15 n=5,245 (50.16%); 16–30 n=2,837 (27.13%); >30 n=2,375 (22.71%).<br>Perceived emergency personnel shortage: Yes n=7,667 (73.32%), No n=2,790 (26.68%).<br>Self-reported health: Good n=1,499 (14.33%), Fair n=5,130 (49.06%), Bad n=3,828 (36.61%).<br>Self-reported sleep quality: Good n=1,087 (10.39%), Fair n=3,227 (30.86%), Bad n=6,143 (58.75%).<br><br>*Descriptive outcomes*<br>Positive affect: 15.20 ± 3.99.<br>Negative affect: 16.76 ± 3.96.<br>Self-efficacy: 25.45 ± 6.36<br>Physical violence: n=2,889 (27.63%).

				Verbal violence: n=8,555 (81.81%).<br><br>*Statistical associations*<br>Physical violence: associated with gender p<0.001, age p<0.001, education p<0.001, marital p=0.016, sleep quality p<0.001, health status p<0.001, professional title p=0.004, years of service p<0.001, night shifts p<0.001, patients/day p<0.001, perceived shortage p<0.001, positive affect p<0.001, negative affect p<0.001, self-efficacy p<0.001.<br><br>Verbal violence: associated with gender p<0.001, age p<0.001, education p<0.001, marital p<0.001, sleep quality p<0.001, health status p<0.001, professional title p<0.001, years of service p<0.001, night shifts p<0.001, patients/day p<0.001, perceived shortage p<0.001, positive affect p<0.001, negative affect p<0.001, self-efficacy p<0.001
Munn et al., [67]<br><br>2025<br><br>United States of America	Cross-sectional, descriptive study<br><br>N/A<br><br>Age, sex, years of experience, job seniority in emergency departments, burnout, work engagement, emergency severity, emergency overcrowding, workplace violence<br><br>n=337<br><br>8/8	To determine job-related factors affecting the well-being of emergency nurses.	Maslach Burnout Inventory (MBI)<br><br>Utrecht Work Engagement Scale (UWES-9)<br><br>Emergency Severity Index<br><br>National Emergency Department Overcrowding Score<br><br>Custom-tailored workplace violence-related questions	*Sample characteristics*<br>Age (years): 34.9 ± 9.5.<br>Sex (N=337): Female n=289 (85.8%), Male n=37 (11.0%), Prefer not to answer n=11 (3.3%).<br>Years of experience: 9.0 ± 8.2.<br>Job seniority in emergency departments: 4.9 ± 5.97.<br><br>*Associations with burnout*<br>Job demands:<br>Patient acuity (emergency departments-level): −15.4 (−25.72, −5.07), p=.02.<br>Emergency department volume per +100 visits: +0.46 (0.08, 0.83), p=.04.<br>Emergency departments overcrowding: +0.03 (−0.03, 0.09), p=.32.<br>Workplace violence: patient/visitor only +5.74 (2.09, 9.40), p=.002; peer only +6.22 (2.98, 9.46), p<.001.<br>Overtime hours: +0.02 (0.01, 0.03), p=.02.<br>Hours/week: 0.00 (−0.15, 0.15), p=.97.<br><br>Job resources:<br>Nurses staffing: +4.91 (1.73, 8.10), p=0.01.<br>Total clinical staffing: +3.00 (1.20, 4.81), p=0.01.<br>Teamwork: −4.00 (−5.03, −2.97), p<0.001.<br>Psychological safety: −4.07 (−5.07, −3.08), p<0.001.<br>Nurse manager support: −2.88 (−3.94, −1.81), p<0.001.

				Sufficient resources: −3.67 (−4.67, −2.67), p<0.001.<br><br>*Associations with work engagement*<br>Job demands:<br>Patient acuity: +0.37 (−0.45, 1.19), p=0.38.<br>Emergency department volume per +100 visits: −0.02 (−0.04, 0.00), p=0.05.<br>Emergency department overcrowding: 0.00 (−0.01, 0.00), p=0.24.<br>Workplace violence: patient/visitor only −0.30 (−0.68, 0.08), p=.12; peer only −0.40 (−0.74, −0.06), p=0.02.<br>Overtime hours: 0.00 (0.00, 0.00), p=0.19.<br>Hours/week: +0.02 (0.01, 0.04), p=0.003.<br><br>Job resources:<br>Nurses staffing: −0.11 (−0.31, 0.10), p=0.33.<br>Total clinical staffing: −0.05 (−0.17, 0.08), p=0.48.<br>Teamwork: +0.40 (0.29, 0.51), p<0.001.<br>Psychological safety: +0.41 (0.31, 0.52), p<0.001.<br>Nurse manager support: +0.32 (0.21, 0.44), p<0.001.<br>Sufficient resources: +0.34 (0.24, 0.45), p<0.001.
Nikolić & Višnjić, [40]<br><br>2020<br><br>Serbia	Cross-sectional, descriptive study<br><br>N/A<br><br>Gender, age, marital status, shift, weekly working hours, work ability, exposure to offensive behaviors<br><br>n=79<br><br>6/8	To examine the presence of mobbing and violence at work, as well as their influence on work ability of emergency medical doctors	Copenhagen Psychosocial Questionnaire (COPSOQ)<br><br>Work Ability Index Questionnaire (WAI)	*Sample characteristics*<br>Gender: Female n=50 (63.3%); Male n=29 (36.7%).<br>Age groups: <30 n=7 (8.9%), 30–39 n=18 (22.8%), 40–49 n=26 (32.9%), 50–59 n=26 (32.9%), ≥60 n=2 (2.5%).<br>Marital status: Married n=60 (75.9%), Live together n=4 (5.1%), Live with parents n=8 (10.1%), Divorced n=3 (3.8%), Single n=4 (5.1%).<br>Shift pattern: Variable hours with night work n=66 (83.5%); primarily 06:00–18:00 n=9 (11.4%); variable without night work n=3 (3.8%); primarily 22:00–06:00 n=1 (1.3%).<br>Weekly working hours: 40.29 ± 3.58<br><br>*Descriptive outcomes*<br>Work Ability scores:<br>Overall WAI: 38.30 ± 1.40.<br>WAI physical demands categories: bad n=1 (1.3%), good n=14 (17.7%), very good n=28 (35.4%), outstanding n=36 (45.6%).<br>WAI mental demands categories: bad n=1 (1.3%), good n=6 (7.6%), very good n=17 (21.5%), outstanding n=55 (69.6%).

Exposure to offensive behaviors:
Sexual harassment: Only a few times n=9 (11.4%), once/month n=1 (1.3%), none n=69 (87.3%). Perpetrator: colleague n=3 (3.8%), subordinate n=4 (5.1%), patients/clients n=4 (5.1%).
Threats of violence: every day n=3 (3.8%), weekly n=4 (5.1%), monthly n=4 (5.1%), few times/year n=27 (34.2%), none n=41 (51.9%). Source: patients/clients n=36 (45.6%), colleague n=2 (2.5%), manager n=1 (1.3%).
Physical violence: weekly n=1 (1.3%), few times/year n=12 (15.2%), none n=66 (83.5%). Source: patients/clients n=13 (16.5%), colleague n=1 (1.3%).
Abuse (mobbing): every day n=1 (1.3%), weekly n=2 (2.5%), monthly n=1 (1.3%), few times/year n=20 (25.3%), none n=55 (69.6%).
Source of violence: patients/clients n=15 (19.0%), colleague n=4 (5.1%), manager n=4 (5.1%), subordinate n=3 (3.8%).

Group comparisons about WAI
Age:
<30 years: $\beta = +1.081$ (95% CI 0.185–1.977), $p = 0.018$.
50–59 years: $\beta = -0.789$ (95% CI −1.281 to −0.296), $p = 0.002$.

Shift pattern:
Primarily 06:00–18:00 (vs others): $\beta = +0.613$ (0.172–1.054), $p = 0.007$.
Variable hours without night work: $\beta = -2.376$ (−3.870 to −0.881), $p = 0.002$.
Variable hours with night work: $\beta = -0.420$ (−0.868 to 0.028), $p = 0.066$ (ns).

Psychosocial work factors (COPSOQ II) predicting WAI
Emotional demands: $\beta = -0.224$ (−0.328 to −0.121), $t = -4.279$, $p < 0.001$.
Commitment to workplace: $\beta = +0.310$ (0.160–0.460), $t = 4.082$, $p < 0.001$.
Predictability: $\beta = +0.147$ (0.006–0.288), $t = 2.055$, $p = 0.042$.
Rewards (recognition): $\beta = +0.163$ (0.026–0.301), $t = 2.354$, $p = 0.020$.
Role clarity: $\beta = +0.248$ (0.079–0.416), $t = 2.910$, $p = 0.004$.
Job satisfaction: $\beta = +0.456$ (0.031–0.882), $t = 2.122$, $p = 0.036$.
Work–family conflict: $\beta = -0.202$ (−0.322 to −0.083), $t = -3.341$, $p = 0.001$.
Self-rated health: $\beta = +0.704$ (0.513–0.894), $t = 7.310$, $p < 0.001$
Burnout: $\beta = -0.283$ (−0.390 to −0.175), $t = -5.190$, $p < 0.001$.
Stress: $\beta = -0.221$ (−0.341 to −0.101), $t = -3.651$, $p < 0.001$.

				(Other COPSOQ dimensions were not significant at α=0.05: quantitative demands p=0.426; work pace p=0.126; influence p=0.069; possibilities for development p=0.178; meaning of work p=0.853; quality of leadership p=0.065; social support from supervisor p=0.361; trust regarding management p=0.064; justice and respect p=0.751.)
Park & Song, [56]<br><br>2023<br><br>South Korea	Cross-sectional, descriptive study<br><br>N/A<br><br>Sex, age, marital status, experience in emergency departments, resilience, turnover intention, nursing work environment, violence frequency, risk of violence<br><br>n=100<br><br>6/8	To analyze the effects of emergency nurses' experiences of violence, resilience, and nursing work environment on turnover intentions.	Experiences of Violence<br><br>Custom-tailored resilience scale<br><br>Practice Environment Scale of the Nursing Work Index<br><br>Turnover Intentions Scale	*Sample characteristics*<br>Sex: Female n=89 (89%); Male n=11 (11%).<br>Age: 27.7 years (SD not reported).<br>Age groups: <30 n=73 (73%); ≥30 n=27 (27%).<br>Marital status: Married n=17 (17%); Single n=83 (83%).<br>Months of experience in emergency departments: 42.6 months (SD not reported).<br>Months of experience in emergency departments (groups): ≤12 n=16 (16%); 13–36 n=42 (42%); ≥37 n=42 (42%).<br><br>*Descriptive outcomes*<br>Resilience: 2.72 ± 0.40 (1–4).<br>Turnover intention: 3.08 ± 0.90 (1–5).<br>Nursing Work Environment: Overall mean = 2.32 (SD not reported). Subscales (total averages): staffing/resource adequacy 1.84 ± 0.57, collegial nurse–physician relations 2.59 ± 0.61, nurse manager ability/leadership/support 2.54 ± 0.56, foundations for quality 2.46 ± 0.42, nurse participation in hospital affairs 2.18 ± 0.52.<br><br>Violence frequency (previous month): Patients: verbal 2.61 ± 1.32, psychological 1.57 ± 1.23, physical 0.51 ± 0.73, severe physical 0.07 ± 0.26, sexual harassment 0.36 ± 0.59, Total 1.03 ± 0.61. Caregivers: verbal 2.49 ± 1.32, psychological 1.37 ± 1.24, physical 0.23 ± 0.58, severe physical 0.05 ± 0.22, sexual harassment 0.15 ± 0.43, Total 0.86 ± 0.56.<br><br>Degree of risk of violence: Patients: verbal 3.37 ± 0.97, psychological 3.06 ± 1.26, physical 2.82 ± 1.68, severe physical 2.49 ± 1.73, sexual harassment 2.03 ± 1.16, Total 2.75 ± 1.04. Caregivers: verbal 3.35 ± 0.99, psychological 2.93 ± 1.26, physical 2.51 ± 1.69, severe physical 2.43 ± 1.74, sexual harassment 1.77 ± 1.03, Total 2.60 ± 1.05.<br><br>*Group comparisons*<br>Resilience by sex: 2.71 ± 0.40 vs 2.86 ± 0.40; t = −1.22, p = 0.224 (ns).<br>Violence frequency (patients) by sex: Female 1.04 ± 0.63 vs Male 0.93 ± 0.51; t = 0.58, p = 0.560 (ns).

				Violence frequency (caregivers) by sex: 0.85 ± 0.57 vs 0.91 ± 0.53; t = −0.32, p = 0.751 (ns).<br>Turnover intention by sex: Female 3.16 ± 0.88 (n=89) vs Male 2.48 ± 0.89 (n=11); t = 2.39, p = 0.019.<br>Turnover intention by age: <30 3.13 ± 0.88 (n=73) vs ≥30 2.96 ± 0.96 (n=27); t = 0.81, p = 0.419 (ns)<br>Turnover intention by experience in emergency departments: ≤12 mo 2.52 ± 0.53 (n=16); 13–36 mo 3.11 ± 0.96 (n=42); ≥37 mo 3.27 ± 0.88 (n=42); F = 4.31, p = 0.016.<br>Nursing Work Environment subscale "nurse participation in hospital affairs" by sex: Female 2.15 ± 0.51 vs Male 2.48 ± 0.57; t = −2.05, p = 0.043.
Powell et al., [61]<br><br>2023<br><br>United States of America	Cross-sectional, descriptive study<br><br>N/A<br><br>Age, sex, years of experience in emergency departments, prevalence of work hazards<br><br>n=13218<br><br>8/8	To evaluate the frequency of occupational hazards and associations with mitigation strategies	Survey about emergency medical services-related characteristics, experiences about occupational injuries, exposures, and violence in the prior 12 months, and mitigation strategies	*Sample characteristics*<br>Age: Median 36 (IQR 29–47)<br>Age groups: <30 n=3,591 (27%), 30–39 n=4,100 (31%), 40–49 n=2,972 (22%), ≥50 n=2,555 (19%). (Mean ± SD not reported.)<br>Sex: Female n=3,682 (28%), Male n=9,508 (72%), Missing n=28.<br>Years of experience in emergency departments: <3 years n=4,211 (32%), 3–7 n=4,132 (31%), ≥8 n=4,851 (38%).<br><br>*Prevalence of work hazards*<br>Any occupational injury: n=3,608 (27%).<br>Back injury n=2,220 (17%), Needlestick n=227 (2%), Other n=1,831 (14%) (among those with injury; multiple types possible).<br><br>Any occupational exposure: n=4,994 (38%).<br>Blood n=3,649 (28%), Smoke n=2,535 (19%), Hazardous chemicals n=715 (5%) (among those with exposure; multiple possible).<br><br>Any occupational violence: n=8,444 (64%).<br>Cursing n=8,228 (62%), Punching n=3,687 (28%), Spitting n=3,290 (25%), Biting n=1,504 (8%), Struck with object n=1,706 (8%), Stabbing n=229 (2%), Shooting n=70 (1%) (multiple possible).
Ramanjaneyulu et al.,[35]<br><br>2024<br><br>India	Cross-sectional, descriptive study<br><br>N/A	To assess the prevalence of physical violence among doctors working in emergency room	Custom-tailored questionnaire based on the Safety Attitudes Questionnaire (SAQ)	*Sample characteristics*<br>Gender: Male n=42 (57.53%), Female n=31 (42.47%).<br>Age groups: <30 n=18 (24.66%), 30–45 n=41 (56.16%), >45 n=14 (19.18%).<br>Workplace violence exposure:<br>Physical violence n=7 (9.59%); Threat of physical violence n=28 (38.36%). Reasons among exposed (multiple responses; exposed n=35): delay in treatment n=29

	Gender, age, workplace violence exposure, teamwork climate, safety climate, job satisfaction, stress, perceptions of management, working conditions, safety attitudes<br><br>n=73<br><br>5/8	and their safety attitudes		(82.86%), patient death n=27 (77.14%), worsening condition n=21 (60.00%), wrong perception of treatment n=16 (45.71%).<br><br>*Descriptive outcomes*<br>Teamwork Climate: 59.29 ± 18.99.<br>Safety Climate: 64.29 ± 13.88.<br>Job Satisfaction: 61.60 ± 13.28.<br>Stress Recognition: 63.44 ± 10.79.<br>Perceptions of Management: 43.75 ± 5.72.<br>Working Conditions: 56.88 ± 9.72.<br>Overall safety attitudes score: 58.21 ± 7.44.<br>Specific safety attitudes items:<br>"Easy to ask questions" 4.01 ± 0.92<br>"Encouraged to report safety concerns" 4.03 ± 0.92;<br>"Difficult to speak up if perceive a problem" 2.27 ± 1.10;<br>"Difficult to discuss errors" 2.70 ± 1.11;<br>"Adequate staffing levels" 2.53 ± 1.36.<br><br>*Group comparisons*<br>Gender: Male 63.12 ± 13.33 (n=42) vs Female 52.21 ± 6.01 (n=31); p = 0.0001.<br>Age group: <30 53.57 ± 8.42 (n=18); 30–45 57.39 ± 6.22 (n=41); >45 63.38 ± 14.04 (n=14); p = 0.009.<br>Exposure to physical violence / threat (any): Yes 51.29 ± 5.88 (n=35) vs No 65.44 ± 14.79 (n=38); p < 0.0001.
Rasmus et al., [51]<br><br>2020<br><br>Poland	Cross-sectional, descriptive study<br><br>N/A<br><br>Sex, age, marital status, years of experience, self-rated physical health, self-rated mental state, family support satisfaction,	To assess the relationship between the number of hours of work and the state of health as well as coping with the stress among emergency medical staff, as factors conditioning the maintenance of work-life balance	Mini-COPE Inventory for Measuring Coping with Stress	*Sample characteristics*<br>Sex: Male n=67 (51.9%), Female n=62 (48.1%).<br>Age (years): 21–30 n=25 (19%), 31–40 n=44 (34%), 41–50 n=40 (31%), 51–60 n=20 (16%).<br>Marital status: Single n=37 (29%), Married n=77 (60%), Widowed n=3 (2%), Separated n=2 (1%), Divorced n=10 (8%).<br>Years of experience: <1 n=6 (5%), 1–2 n=7 (5%), 3–5 n=15 (11%), 6–10 n=22 (18%), >10 n=79 (61%).<br><br>*Descriptive outcomes*<br>Self-rated physical health (1–5 scale): Both sexes predominantly rated 4–5; sex difference: p = 0.256404 (ns).<br>Self-rated mental state (1–5 scale): Higher in men than women; sex difference: p = 0.026640.

	monthly hours worked<br><br>n=129<br><br>5/8			Family support satisfaction: Yes n=121 (93.8%), No n=8 (6.2%); sex difference: p = 0.282521 (ns).<br><br>*Correlations*<br>Monthly hours worked and Age: r = −0.156, p = 0.077.<br>Monthly hours worked and Self-rated physical health: r = −0.121, p = 0.171.
Ren et al., [52]<br><br>2025<br><br>China	Cross-sectional, descriptive study<br><br>N/A<br><br>Age, gender, marital status, years of experience, weekly working hours, environmental noise levels, mental health issues symptoms<br><br>n=126<br><br>6/8	To explore the effects of long-term noise exposure on the mental health and sleep quality of medical staff in an emergency department and noise-coping strategies.	Ambient noise was monitored using 2270-S sound level meters<br><br>Symptom Checklist-90 Revised (SCL-90-R)<br><br>Pittsburgh Sleep Quality Index (PSQI)<br><br>Polysomnography monitoring	*Sample characteristics*<br>Doctors: General inpatient (n=40) vs emergency department (n=49):<br>Age: <31 n=8 (20.0%) vs n=10 (20.41%); 31–40 n=18 (45.0%) vs n=15 (30.61%); >40 n=14 (35.0%) vs n=24 (48.98%); χ^2=2.239, p=0.326.<br>Gender: Male n=29 (72.5%) vs n=30 (61.22%); Female n=11 (27.5%) vs n=19 (38.78%); χ^2=1.253, p=0.263.<br>Marital status: Married n=22 (55.0%) vs n=25 (51.02%); Unmarried n=13 (32.5%) vs n=20 (40.82%); Divorced n=5 (12.5%) vs n=4 (8.16%); χ^2=0.886, p=0.642.<br>Years of experience: ≤10 years n=18 (45.0%) vs n=29 (59.18%); >10 years n=22 (55.0%) vs n=20 (40.82%); χ^2=1.778, p=0.182.<br>Weekly working time: <41 h n=12 (30.0%) vs n=12 (24.49%); 41–60 h n=20 (50.0%) vs n=22 (44.90%); >60 h n=8 (20.0%) vs n=15 (30.61%); χ^2=1.329, p=0.514.<br><br>Nurses: General inpatient (n=60) vs emergency department (n=77):<br>Age: <31 n=28 (46.67%) vs n=32 (41.56%); 31–40 n=22 (36.67%) vs n=31 (40.26%); >40 n=10 (16.67%) vs n=14 (18.18%); χ^2=0.358, p=0.836.<br>Gender: Male n=15 (25.0%) vs n=30 (38.96%); Female n=45 (75.0%) vs n=47 (61.04%); χ^2=2.980, p=0.084.<br>Marital status: Married n=27 (45.0%) vs n=40 (51.95%); Unmarried n=29 (48.33%) vs n=31 (40.26%); Divorced n=4 (6.67%) vs n=6 (7.79%); χ^2=0.893, p=0.640.<br>Years of experience: ≤10 years n=38 (63.33%) vs n=52 (67.53%); >10 years n=22 (36.67%) vs n=25 (32.47%); χ^2=0.264, p=0.607.<br>Weekly working time: <41 h n=14 (23.33%) vs n=20 (25.97%); 41–60 h n=31 (51.67%) vs n=35 (45.45%); >60 h n=15 (25.00%) vs n=22 (28.57%); χ^2=0.524, p=0.769.<br><br>*Descriptive outcomes*<br>Environmental noise levels:<br>Daytime: emergency department 72.42 ± 3.89 vs General 65.26 ± 3.57; t=14.249, p<0.001.<br>Night-time: emergency department 59.33 ± 4.87 vs General 46.34 ± 4.02; t=21.487, p<0.001.

SCL-90-R: Emergency department vs General
Somatization: 1.82 ± 0.43 vs 1.48 ± 0.49; $t=5.549$, $p<0.001$.
Obsessive–compulsive: 1.98 ± 0.50 vs 1.72 ± 0.40; $t=4.234$, $p<0.001$.
Interpersonal sensitivity: 1.70 ± 0.35 vs 1.73 ± 0.43; $t=0.579$, $p=0.564$ (ns).
Depression: 1.77 ± 0.35 vs 1.62 ± 0.43; $t=2.891$, $p=0.004$.
Anxiety: 1.73 ± 0.30 vs 1.63 ± 0.44; $t=2.026$, $p=0.044$.
Hostility: 1.84 ± 0.42 vs 1.72 ± 0.37; $t=2.248$, $p=0.026$.
Phobic anxiety: 1.58 ± 0.32 vs 1.33 ± 0.32; $t=5.833$, $p<0.001$.
Paranoid ideation: 1.53 ± 0.30 vs 1.42 ± 0.28; $t=2.819$, $p=0.005$.
Psychoticism: 1.67 ± 0.40 vs 1.40 ± 0.23; $t=6.006$, $p<0.001$.
Global Severity Index: 1.74 ± 0.37 vs 1.56 ± 0.38; $t=3.589$, $p<0.001$.

Pittsburgh Sleep Quality Index (PSQI): Emergency department vs General
Subjective sleep quality: 1.52 ± 0.48 vs 0.83 ± 0.22; $t=13.304$, $p<0.001$.
Sleep latency: 1.62 ± 0.43 vs 0.87 ± 0.21; $t=15.980$, $p<0.001$.
Sleep duration: 1.70 ± 0.55 vs 1.21 ± 0.36; $t=7.695$, $p<0.001$.
Sleep efficiency: 0.89 ± 0.22 vs 0.65 ± 0.15; $t=9.322$, $p<0.001$.
Sleep disturbances: 1.11 ± 0.32 vs 0.73 ± 0.19; $t=10.495$, $p<0.001$.
Use of sleeping medications: 1.28 ± 0.35 vs 0.70 ± 0.23; $t=14.298$, $p<0.001$.
Daytime dysfunction: 0.95 ± 0.19 vs 0.68 ± 0.20; $t=10.366$, $p<0.001$.

Polysomnography: Emergency department vs General
Total sleep duration (min): 326.43 ± 51.36 vs 405.47 ± 51.13; $t=11.514$, $p<0.001$.
Sleep latency (min): 37.35 ± 10.45 vs 21.25 ± 6.76; $t=13.346$, $p<0.001$.
Awakening time (min): 66.42 ± 20.05 vs 48.30 ± 10.41; $t=8.195$, $p<0.001$.
Sleep efficiency (%): 72.36 ± 7.19 vs 78.65 ± 6.84; $t=6.674$, $p<0.001$.

Correlations with noise exposure
Daytime noise: Global Severity Index $r=0.326$, $p<0.001$; PSQI total $r=0.298$, $p=0.021$; Sleep duration $r=-0.138$, $p=0.200$; Sleep latency $r=0.192$, $p=0.141$; Awakening time $r=0.092$, $p=0.484$; Sleep efficiency $r=-0.196$, $p=0.134$.

Night-time noise: Global Severity Index $r=0.435$, $p<0.001$; PSQI total $r=0.515$, $p<0.001$; Sleep duration $r=-0.503$, $p<0.001$; Sleep latency $r=0.422$, $p<0.001$; Awakening time $r=0.261$, $p<0.001$; Sleep efficiency $r=-0.293$, $p<0.001$.

Sánchez Onrubia et al.,[69]<br><br>2025<br><br>Spain	Cross-sectional, descriptive study<br><br>N/A<br><br>Sex, age, marital status, years of experience, job seniority in emergency departments, job satisfaction, work-life balance, burnout, sleep quality<br><br>n=176 (n=74 in the first data collection (T1), and n=102 in the second (T2))<br><br>6/8	To know job satisfaction, work-family balance, sleep quality, and burnout in nursing staff after the introduction of the 12-hour rotating shift in the emergency department	Maslach Burnout Inventory (MBI)<br><br>Pittsburgh Sleep Quality Index (PSQI)<br><br>Custom-tailored job satisfactions and work-life balance questions	*Sample characteristics*<br>Sex: T1 Male n=10 (13.5%), Female n=64 (86.5%) (p=0.496). T2 Male n=11 (10.8%), Female n=91 (89.2%) (p=0.601; T1 vs T2 p=0.581).<br>Age (years, mean ± SD): T1 overall 44.72 ± 10.61; T2 overall 44.76 ± 11.07<br>Marital status: T1 Single n=16 (21.6%), Partner n=22 (29.7%), Married n=35 (47.3%), Divorced n=1 (1.4%) (p=0.308). T2 18 (17.6%) / 27 (26.5%) / 56 (54.9%) / 1 (1.0%) (p=0.645; T1 vs T2 p=0.542).<br>Years of experience: T1 15.40 ± 8.44; T2 16.38 ± 9.48<br>Job seniority in emergency departments: T1 5.47 ± 5.57; T2 5.11 ± 6.41<br><br>*Descriptive outcomes*<br>Job satisfaction:<br>Current job (this hospital): T1 n=48 (65.8%); T2 n=67 (70.5%); T1 vs T2 p=0.035.<br>Schedule flexibility: T1 52 (71.2%); T2 64 (66.7%); T1 vs T2 p=0.644.<br>Professional development opportunities: T1 40 (54.8%); T2 53 (55.8%); T1 vs T2 p=0.345.<br>Autonomy: T1 43 (58.9%); T2 58 (61.1%); T1 vs T2 p=0.447.<br>Professional status: T1 44 (61.2%); T2 69 (71.9%); T1 vs T2 p=0.367.<br>Salary: T1 27 (37.5%); T2 38 (40.4%); p=0.827.<br>Vacations: T1 28 (39.4%); T2 42 (43.8%); T1 vs T2 p=0.160.<br>Sick-leave rights: T1 44 (62.0%); T2 55 (58.5%); T1 vs T2 p=0.552.<br>Profession choice: T1 60 (82.2%); T2 87 (90.6%); T1 vs T2 p=0.215.<br>Would not leave the emergency department next year due to job dissatisfaction (retention intention): T1 65 (89.0%); T2 79 (82.3%); T1 vs T2 p=0.210.<br><br>Work-life balance:<br>Enjoy family & friends: T1 48 (65.8%); T2 63 (66.3%); T1 vs T2 p=0.501.<br>Enjoy other leisure time: T1 41 (56.2%); T2 46 (48.4%); T1 vs T2 p=0.506.<br>Do housework: T1 58 (79.5%); T2 81 (85.3%); T1 vs T2 p=0.278.<br>Time for oneself: T1 28 (38.4%); T2 33 (34.7%); T1 vs T2 p=0.280.<br>Time for other social needs: T1 26 (35.6%); T2 34 (35.8%); T1 vs T2 p=0.761.<br>Flexibility to swap shifts with colleagues: T1 41 (56.2%); T2 57 (60.6%); T1 vs T2 p=0.831.<br><br>Overall burnout (based on ≥2 domains at the level):<br>T1: Low n=40 (71.4%), Medium n=11 (19.6%), High n=5 (8.9%).<br>T2: Low n=56 (71.8%), Medium n=15 (17.9%), High n=8 (10.3%).

				T1 vs T2 p=0.972.<br><br>Burnout by domain levels and overall categories:<br>T1: emotional exhaustion high n=11 (15.3%), depersonalization high n=13 (19.1%), personal accomplishment low n=47 (69.1%).<br>T2: emotional exhaustion high n=19 (20.4%), depersonalization high n=16 (17.4%), personal accomplishment low n=73 (76.8%)<br><br>Overall sleep quality: T1 7.66 ± 3.82; T2 8.46 ± 4.05; T1 vs T2 p=0.255.<br><br>Sleep quality components:<br>Subjective sleep quality: T1 1.27 ± 0.71; T2 1.51 ± 0.75; T1 vs T2 p=0.074.<br>Sleep latency: T1 1.60 ± 1.03; T2 1.84 ± 0.95; T1 vs T2 p=0.320.<br>Sleep duration: T1 1.34 ± 1.01; T2 1.30 ± 0.87; T1 vs T2 p=0.775.<br>Habitual sleep efficiency: T1 0.95 ± 1.03; T2 0.87 ± 1.00; T1 vs T2 p=0.271.<br>Sleep disturbances: T1 1.20 ± 0.48; T2 1.27 ± 0.50; T1 vs T2 p=0.551.<br>Use of sleeping medication: T1 0.41 ± 0.85; T2 0.54 ± 1.01; T1 vs T2 p=0.736.<br>Daytime dysfunction: T1 0.87 ± 0.83; T2 1.13 ± 0.94; T1 vs T2 p=0.199.
Sánchez-Zaballos & Mosteiro-Díaz,[50]<br><br>2021<br><br>Spain	Cross-sectional, descriptive study<br><br>N/A<br><br>Sex, age, marital status, work, years of professional experience, job seniority in emergency departments, resilience<br><br>n=320<br><br>8/8	To determine the resilience of professional health workers in emergency services and its relationships with sociodemographic and working conditions	Resilience Scale-25 (RS-25)	*Sample characteristics*<br>Sex: women n=262 (81.87%), men n=58 (18.13%).<br>Age: 43.5 ± 8.9 years.<br>Marital status: Married/in a partnership n=210 (65.63%), Single n=80 (25.00%), Divorced n=26 (8.12%), Widowed n=4 (1.25%).<br>Professional category: Physicians n=86 (26.9%), Nurses n=154 (48.1%), Nursing assistants n=80 (25.0%)<br>Night work: Not working night shifts n=49 (15.31%); including night shifts n=271 (84.69%).<br>Years of professional experience: 16.8 ± 8.4 years.<br>Job seniority in emergency departments: 9.1 ± 6.9 years.<br><br>*Descriptive outcomes*<br>RS-25 total: 133.52 ± 7.22 (authors classify as “moderately low to moderate”).<br>RS-25 level categories: Very low n=45 (14.1%), Moderate to moderately low n=220 (62.5%), Moderately high to high n=75 (23.4%)<br><br>*Group comparisons*<br>Overall resilience:

				By professional category: Physicians 134.72 ± 18.26, Nurses 128.74 ± 15.76, Nursing assistants 130.60 ± 21.11; Kruskal-Wallis χ^2=8.84, p=0.01.<br><br>By marital status (whole sample): Single 124.38 ± 21.20, Married/partnered 133.40 ± 16.27, Divorced 125.89 ± 19.92; ANOVA F=3.69, p=0.01 (Bonferroni: single < partnered, p=0.02).<br><br>By night work (whole sample): Not night shifts 126.36 ± 21.58 vs Including night shifts 131.48 ± 17.37; Mann-Whitney Z=−2.10, p=0.03.<br><br>Nursing assistants by night work: Not night 118.20 ± 24.94 vs Including night 135.50 ± 17.43; t=−3.08, p<0.001.<br><br>Physicians by marital status: Single 119.00 ± 33.43, Married/partnered 139.46 ± 9.82, Divorced 134.00 ± 10.93; ANOVA F=7.16, p<0.001.<br><br>*Resilience levels*<br>By professional category: Physicians: very low n=6 (7.0%), moderate n=60 (69.8%), high n=20 (23.3%); Nurses: n=24 (15.6%) / 104 (67.5%) / 26 (16.9%); Nursing assistants: n=15 (18.8%) / 36 (45.0%) / 29 (36.3%); χ^2=18.27, p<0.001.<br><br>By sex: Female: very low n=41 (15.65%), moderate n=154 (58.78%), high n=67 (25.77%); Male: n=4 (6.90%) / 46 (79.31%) / 8 (13.79%); χ^2=8.60, p=0.01.<br><br>By performance area: Hospital ED: very low n=44 (16.1%), moderate n=167 (60.9%), high n=63 (23.0%); EMUs: n=1 (2.2%) / 33 (71.7%) / 12 (26.1%); χ^2=6.29, p=0.04.<br><br>Nursing assistants only by night work: Not night: n=9 (50.0%) / 6 (33.3%) / 3 (16.7%); Including night: n=5 (8.3%) / 30 (50.0%) / 25 (41.7%); χ^2=16.63, p<0.001.<br><br>Physicians only by resilience capacity: Age (means) very low 35.5 ± 8.68, moderate 44.54 ± 7.25, high 42.16 ± 9.65; ANOVA F=3.59, p=0.03.
Schablon et al., [49]<br><br>2022	Cross-sectional, descriptive study<br><br>N/A	To generate data on the frequency of violence by patients and accompanying	Custom-tailored workplace violence and perceived stress questions	*Sample characteristics*<br>Gender: women n=202 (57.9%), men n=146 (41.8%; 1 missing).<br>Age: 20–29 n=100 (28.7%), 30–39 n=98 (28.1%), 40–49 n=80 (22.9%), 50–59 n=59 (16.9%), ≥60 n=11 (3.2%; 1 missing).

Germany	Gender, age, occupation, violence exposure, perceived stress, burnout<br><br>n=349<br><br>8/8	relatives and the correlation between experienced aggression, a possible risk of burnout and a high sense of stress	Copenhagen Burnout Inventory (CBI)<br><br>Resilience Scale-13 (RS-13)	*Descriptive outcomes*<br>Exposure to violence in the last 12 months:<br>Physical violence from patients: n=305 (87.4%); relatives: n=225 (64.5%).<br>Verbal abuse from patients: n=339 (97.1%); relatives: n=329 (94.3%).<br>Change during COVID access restrictions: decreased 28.4% (n=99/349), same 45.0% (n=157/349), increased 25.8% (n=90/349).<br><br>Perceived stress due to violence (categorical). Low n=68 (19.5%), moderate n=195 (55.9%), high n=79 (22.6%; 7 missing).<br><br>Perceived preparation by workplace (categorical). Insufficient n=159 (45.6%), moderate n=154 (44.1%), good n=34 (9.7%; 2 missing).<br><br>Resilience (RS-13 categories). Low n=101 (28.9%), moderate n=80 (22.9%), high n=155 (44.4%; 13 missing).<br><br>Burnout:<br>Work-related CBI: moderate–high = 42% ⇒ n=147<br>Patient-related CBI: moderate–high = 17% ⇒ n=59<br><br>*Associations*<br>Burnout and perceived stress:<br><br>Gender: male CBI (work) 40.4 ± 19.0; CBI (patient) 27.7 ± 17.1; perceived stress 5.4 ± 2.5. Female 46.4 ± 20.2; 31.3 ± 18.8; 5.9 ± 2.2.<br><br>Age groups: 20–29 45.1 ± 21.1; 32.7 ± 17.0; 5.8 ± 2.4. 30–39 45.5 ± 21.2; 30.1 ± 20.1; 5.6 ± 2.3. 40–49 43.2 ± 17.6; 29.8 ± 18.6; 5.8 ± 2.6. 50–59 40.5 ± 18.5; 25.1 ± 16.0; 5.5 ± 2.2. ≥60 40.9 ± 19.8; 26.3 ± 14.5; 5.1 ± 1.7.<br><br>Occupation: physician 44.1 ± 19.1; 26.8 ± 18.2; 5.4 ± 2.6. Nurse 44.8 ± 19.5; 31.0 ± 17.3; 5.8 ± 2.1. Paramedic 30.2 ± 16.6; 22.9 ± 16.2; 4.2 ± 2.4. Other 48.3 ± 23.2; 35.0 ± 22.4; 6.2 ± 2.5.<br><br>Time in ED: 0–5 y 46.7 ± 21.1; 32.6 ± 16.5; 5.8 ± 2.3. 6–10 y 45.6 ± 18.7; 31.0 ± 18.6; 5.7 ± 2.3. 11–15 y 45.8 ± 21.8; 30.7 ± 20.7; 5.8 ± 2.2. >15 y 38.7 ± 17.7; 25.3 ± 17.2; 5.3 ± 2.5.

				Physical violence from patients (frequency): never 36.5 ± 20.6; 22.3 ± 14.0; 4.4 ± 2.5. Once/year 41.6 ± 18.1; 27.4 ± 15.3; 5.4 ± 2.3. Once/quarter 43.7 ± 20.1; 29.3 ± 18.6; 5.6 ± 2.3. Once/month 46.1 ± 19.5; 31.1 ± 19.6; 6.0 ± 2.2. Once/week 52.8 ± 19.0; 38.6 ± 18.7; 6.8 ± 1.8. Daily 40.0 ± 22.1; 35.8 ± 17.1; 7.0 ± 2.7.<br><br>Physical violence from relatives (frequency): never 39.6 ± 20.1; 25.7 ± 16.3; 4.8 ± 2.3. Once/year 46.0 ± 18.6; 30.8 ± 18.6; 5.8 ± 2.2. Once/quarter 43.2 ± 19.3; 30.6 ± 17.7; 6.0 ± 2.2. Once/month 50.7 ± 21.0; 33.0 ± 21.8; 6.7 ± 2.2. Once/week 49.8 ± 20.0; 38.5 ± 16.6; 7.0 ± 1.4. Daily 30.4 ± 17.7; 29.2 ± 11.8; 5.5 ± 3.5.<br><br>Verbal abuse from patients (frequency): never 30.8 ± 20.5; 16.2 ± 9.8; 3.5 ± 3.0. Once/year 36.6 ± 19.5; 25.5 ± 17.9; 4.8 ± 2.7. Once/quarter 40.8 ± 19.2; 24.5 ± 13.7; 4.8 ± 2.2. Once/month 39.9 ± 20.5; 24.7 ± 15.8; 5.2 ± 2.2. Once/week 44.9 ± 17.9; 32.4 ± 18.1; 6.0 ± 2.3. Daily 52.0 ± 20.5; 37.5 ± 21.0; 6.6 ± 2.0.<br><br>Verbal abuse from relatives (frequency): never 31.2 ± 15.7; 16.2 ± 12.7; 2.6 ± 1.8. Once/year 38.5 ± 18.0; 22.7 ± 15.0; 4.7 ± 2.6. Once/quarter 41.6 ± 18.7; 26.6 ± 15.0; 5.1 ± 2.2. Once/month 41.2 ± 20.6; 26.8 ± 17.0; 5.7 ± 2.2. Once/week 47.2 ± 19.3; 32.2 ± 17.2; 6.0 ± 2.2. Daily 50.6 ± 20.2; 39.3 ± 21.4; 6.7 ± 1.9.
Senken et al., [70]<br><br>2024<br><br>United States of America	Cross-sectional, descriptive study<br><br>N/A<br><br>Roles, job satisfaction<br><br>n=755<br><br>5/8	To use a rapid assessment tool to identify factors that impact emergency worker satisfaction, or "wellness," while on shift in the emergency department and the association with role and level of satisfaction.	Custom-tailored questionnaire about job satisfaction	*Sample characteristics*<br>Roles: Nurses n=400, Attending physicians n=143, Learners n=144 (residents/fellows/med students), Ancillary staff n=68.<br>Other demographics were not collected<br><br>*Descriptive outcomes*<br>Overall satisfaction: Dissatisfied n=467 (62.1%); Satisfied n=288 (38.2%)<br>Satisfaction by role:<br>Nurses (n=400): Dissatisfied n=290 (72.5%), Satisfied n=110 (27.5%).<br>Attending physicians (n=143): Dissatisfied n=78 (54.5%), Satisfied n=65 (45.5%).<br>Learners (n=144): Dissatisfied n=62 (43.1%), Satisfied n=82 (56.9%).<br>Ancillary staff (n=68): Dissatisfied n=37 (54.4%), Satisfied n=31 (45.6%).<br><br>*Factors related to job satisfaction (Dissatisfied n=467 vs Satisfied n=288.)*<br>Admission/transfer process: n=46 (9.9%) vs n=12 (4.2%); $p < 0.01$.<br>Boarding patients: n=85 (18.2%) vs n=8 (2.8%); $p < 0.01$.<br>Equipment/stocking/tools: n=153 (32.8%) vs n=69 (24.0%); $p = 0.01$.<br>Patient flow through department: n=188 (40.3%) vs n=94 (32.6%); $p = 0.04$.

				Teaching/learning: n=41 (8.8%) vs n=62 (21.5%); p < 0.01.<br>Team/coworker interaction: n=182 (39.0%) vs n=245 (85.1%); p < 0.01.
Tan et al., [66]<br><br>2025<br><br>China	Cross-sectional, descriptive study<br><br>N/A<br><br>Gender, age, marital status, shift, effort-reward imbalance, burnout, sleep disorders, somatization, work-family conflict<br><br>n=1540<br><br>8/8	To analyze if work–family conflict moderates the mediating influence emotional exhaustion has on the association between effort-reward imbalance (ERI) and somatic symptoms and sleep disorders.	ERI questionnaire<br><br>Maslach Burnout Inventory (MBI)<br><br>Self-administered Sleep Questionnaire (SSQ)<br><br>Somatization Symptom Self-Rating ScaleChina (SSS-CN)<br><br>Work-Family Behavioral Role Conflict Scale (WFBRC-S)	*Sample characteristics*<br>Gender: Female n=1,211 (78.6%), Male n=329 (21.4%).<br>Age: 32.23 ± 6.80 y (range 20–58).<br>Marital status: Unmarried n=560 (36.4%), Married n=980 (63.6%).<br>Night shift: Yes n=1,359 (88.2%); No n=181 (11.8%).<br>Night shifts/month: 0 n=181 (11.8%), 1–4 n=239 (15.5%), 5–8 n=659 (42.8%), >8 n=461 (29.9%).<br>Working hours/week: ≤40 h n=577 (37.5%), 41–48 h n=780 (50.6%), 49–58 h n=123 (8.0%), ≥59 h n=60 (3.9%).<br><br>*Descriptive outcomes*<br>ERI index: 0.93 ± 0.57; ERI>1 (imbalance): n=403 (26.2%).<br>Emotional exhaustion: 11.30 ± 7.76.<br>Work–family conflict: 42.48 ± 16.21.<br>Somatic symptoms (total): 39.58 ± 13.61<br>Physical disorder 19.13 ± 6.87<br>Psychological disorder 20.46 ± 7.08.<br>Sleep disorders (SSQ total): 8.56 ± 3.12.<br><br>*Correlations*<br>ERI with emotional exhaustion r=0.624 (p<0.01)<br>ERI with work-family conflict r=0.552 (p<0.01)<br>ERI with somatic symptoms r=0.554 (p<0.01)<br>ERI with sleep disorders r=0.335 (p<0.01).
Tang et al.,[62]<br><br>2024<br><br>China	Cross-sectional, descriptive study<br><br>N/A<br><br>Sex, age, marital status, occupation, departmental noise, burnout, intent to leave	To explore the effect of noise in the emergency department on the occupational burnout and the resignation intentions of medical staff.	dBadge2 individual noise analyser<br><br>Maslach Burnout Inventory (MBI)<br><br>Intent to Leave Scale	*Sample characteristics (group A)*<br>Sex: Male n=23 (54.76%), Female n=19 (45.24%)<br>Age: 36.50 [30.75–39.25] years (median [IQR])<br>Marital status: Married n=25 (59.52%), Unmarried n=17 (40.48%)<br>Working time per shift (hours): 12.00 [11.00–13.00]<br><br>*Descriptive outcomes (group A)*<br>Departmental noise (dB): 58.67 ± 8.27<br>Emotional fatigue: 26.00 [21.75–30.00]<br>Work apathy: 14.00 [11.00–17.00]<br>Sense of achievement (reverse-scored): 31.00 [29.00–33.00]

	n=81 (n=42 in group A/emergency department, n=39 in group B/rehabilitation)<br><br>6/8			Resignation intention I: 5.00 [3.75–6.00]<br>Resignation intention II: 5.00 [3.00–6.00]<br>Resignation intention III: 5.00 [4.00–6.00]
Tong et al., [65]<br><br>2024<br><br>China	Cross-sectional, descriptive study<br><br>N/A<br><br>Gender, age, marital status, years of experience, shift, effort-reward imbalance, somatization, work-family conflict<br><br>n=1540<br><br>8/8	To explore the interaction between effort-reward imbalance (ERI) and work-family conflict (WFC) among emergency nurses, with a particular focused on the mediating role of WFC and the moderating effect of intrinsic effort.	ERI scale<br><br>Somatization Symptom Self-Assessment Scale (SSD-CN)<br><br>Work Family Behavioral Role Conflict Scale (WFBRC-S)	*Sample characteristics*<br>Gender: Female n=1,211 (78.6%), Male n=329 (21.4%).<br>Age: 32.23 ± 6.80 years (range 20–58).<br>Marital status: Unmarried n=560 (36.4%), Married n=980 (63.6%).<br>Years of experience: 1–2 n=242 (15.7%), 3–10 n=742 (48.2%), 11–20 n=421 (27.3%), >20 n=135 (8.8%).<br>Night shift: Yes n=1,359 (88.2%), No n=181 (11.8%).<br>Night shifts/month: 0 n=181 (11.8%), 1–4 n=239 (15.5%), 5–8 n=659 (42.8%), >8 n=461 (29.9%).<br>Weekly hours: ≤40 n=577 (37.5%), 41–48 n=780 (50.6%), 49–58 n=123 (8.0%), ≥59 n=60 (3.9%).<br><br>*Descriptive outcomes*<br>ERI ratio: 0.932 ± 0.57<br>ERI prevalence n=1,240 (80.5%)<br>Intrinsic effort: 12.60 ± 4.74 (range 5–25).<br>WFC total: 42.48 ± 16.21 (work to family 18.27 ± 6.88; family to work 24.21 ± 10.75).<br>Symptoms:<br>Overall symptoms 39.58 ± 13.61<br>Somatic 19.13 ± 6.87<br>Mental 20.46 ± 7.08.<br><br>*Correlations*<br>ERI with: WFC r=0.552 (p<0.01)<br>Overall symptoms r=0.554 (p<0.01)<br>Somatic r=0.547 (p<0.01)<br>Mental r=0.533 (p<0.01)<br>Intrinsic effort r=0.712 (p<0.01).<br><br>WFC with:

				Overall symptoms r=0.655<br>Somatic r=0.660<br>Mental r=0.618 (all p<0.01).
Ulmann et al.,[39]<br><br>2025<br><br>Switzerland	Cross-sectional, descriptive study<br><br>N/A<br><br>Gender, age, years of experience, job seniority in emergency departments, self-rated physical, mental and social health<br><br>n=539<br><br>8/8	To record the self-assessed health status of emergency nurses from acute hospitals in German-speaking Switzerland	Custom-tailored questionnaire based on sub-surveys of the Swiss Health Survey (SGB)	*Sample characteristics*<br>Gender: Female n=469 (87.0%), Male n=63 (11.7%), Diverse n=3 (0.6%).<br>Age (years): 40.0 ± 10.7<br>Age (groups): 20–29 n=101 (18.7%), 30–39 n=177 (32.8%) 40–49 n=137 (25.4%), 50–59 n=97 (18.0%), 60–66 n=18 (3.3%).<br>Years of experience: 19.6 ± 9.95<br>Years of experience (groups): 0–5 n=25 (4.6%), 6–10 n=82 (15.2%), 11–20 n=210 (39.0%), >20 n=209 (38.8%).<br>Job seniority in emergency departments (years) 10.7 ± 8.54<br>Job seniority in emergency departments (groups) 0–5 n=195 (36.2%), 6–10 n=115 (21.3%), 11–20 n=145 (26.9%), >20 n=78 (14.5%).<br><br>*Descriptive outcomes*<br>Self-rated health<br>Categories: Very good/good n=434 (80.5%); Moderate n=89 (16.5%); Bad/very bad n=15 (2.8%).<br><br>Physical health<br>Back/low-back pain: Not at all n=144 (26.7%), A little n=306 (56.8%), Severe n=83 (15.4%).<br>General weakness/fatigue/low energy: Not at all n=73 (13.5%), A little n=311 (57.7%), Severe n=154 (28.6%).<br>Abdominal pain/pressure: Not at all n=377 (69.9%), A little n=137 (25.4%), Severe n=20 (3.7%).<br>Diarrhea/constipation/both: Not at all n=348 (64.6%), A little n=152 (28.2%), Severe n=32 (5.9%).<br>Sleep-onset/maintenance problems: Not at all n=149 (27.6%), A little n=266 (49.4%), Severe n=120 (22.3%).<br>Headache/pressure/face pain: Not at all n=225 (41.7%), A little n=236 (43.8%), Severe n=75 (13.9%).<br>Palpitations: Not at all n=394 (73.1%), A little n=125 (23.2%), Severe n=17 (3.2%).<br>Chest pain/pressure: Not at all n=468 (86.8%), A little n=61 (11.3%), Severe n=7 (1.3%).<br>Fever: Not at all n=457 (88.1%), A little n=42 (7.8%), Severe n=13 (2.4%).

Shoulder/neck/arm pain: Not at all n=174 (32.3%), A little n=238 (44.2%), Severe n=124 (23.0%).
Treatment due to physical complaints: n=316 (58.2%).

Mental health
"Little interest/pleasure": Not at all n=150 (27.8%), Several days n=311 (57.7%), >Half the days n=57 (10.6%), Nearly every day n=14 (2.6%).
Depressed mood/hopelessness: n=274 (50.8%) / 210 (39.0%) / 36 (6.7%) / 9 (1.7%) (ordered as above).
Sleep problems (insomnia/hypersomnia): n=114 (21.2%) / 263 (48.8%) / 113 (21.0%) / 42 (7.8%).
Fatigue/low energy: n=65 (12.1%) / 303 (56.2%) / 112 (20.8%) / 51 (9.5%).
Appetite decrease/increase: n=260 (48.2%) / 192 (35.6%) / 57 (10.6%) / 21 (3.9%).
Negative self-perception: n=295 (54.7%) / 194 (36.0%) / 32 (5.9%) / 7 (1.3%).
Concentration difficulties: n=283 (52.5%) / 203 (37.7%) / 33 (6.1%) / 8 (1.5%).
Psychomotor change (slowed/restless): n=410 (76.1%) / 101 (18.7%) / 11 (2.0%) / 4 (0.7%).
Suicidal thoughts/self-harm thoughts: n=507 (94.1%) / 19 (3.5%) / 1 (0.2%) / 1 (0.2%).
"Indications of major depression": None/minimal n=206 (38.2%), Mild to severe n=320 (59.4%).
Psychological treatment within the last year: n=39 (7.2%).

Social health
Number of close persons: 0 n=1 (0.2%), 1–2 n=81 (15.0%), 3–5 n=265 (49.2%), ≥6 n=186 (34.5%).
People available any time to discuss problems: Yes, several persons n=450 (83.5%); Yes, one person n=78 (14.5%); No n=5 (0.9%).
Participation in social activities: ≥1×/week n=170 (31.6%), ≥1×/month n=180 (33.4%), A few times/year n=90 (16.7%), Rarely/never n=93 (17.2%).

Associations
Age and physical/mental/social indicators:
General weakness/fatigue (physical) V=0.166, $p<0.05$; youngest (20–29) and oldest (≥60) reported severe most often; 40–49 least often.
Abdominal pain/pressure V=0.132, $p<0.05$; higher in youngest/oldest.
Diarrhea/constipation V=0.133, $p<0.05$; higher in youngest/oldest.
Headache/face pain V=0.128, $p<0.05$; most frequent in 20–39.

				Treatment due to physical complaints V=0.178, p<0.05; lowest in 40–49.<br>Fatigue/low energy (mental item) V=0.133, p<0.05; decreases with age.<br>"Major depression" indication V=0.176, p<0.05; decreases with age; ≥60 least frequent.<br>Number of close persons V=0.156, p<0.05; tends to decrease with age (50–59 and ≥60 most often report 0–2 persons).<br><br>Job seniority in emergency departments and symptoms:<br>Headache/face pain V=0.112, p<0.05; declines with more ED years.<br>Fatigue/low energy (mental item) V=0.110, p<0.05; declines with more ED years.<br>PHQ-9 "major depression" indication V=0.161, p<0.05; lowest in >20 ED years.<br>Other tested symptom links; not significant at α=0.05
Vitale et al., [59]<br><br>2021<br><br>Italy	Cross-sectional, descriptive study<br><br>N/A<br><br>Sex, role, job seniority in emergency departments, shift, violence, job satisfaction, burnout<br><br>n=222<br><br>6/8	To evaluate potential risk factors in the development of burnout syndrome in physicians and registered nurses belonging to the Italian Emergency Medicine Departments.	Custom-tailored violence questionnaire<br><br>Maslach Burnout Inventory (MBI)	*Sample characteristics*<br>Sex: Female n=127 (57.2%), Male n=95 (42.8%).<br>Role: Physicians n=47 (21.2%), Registered nurses n=175 (78.8%).<br>Job seniority in emergency departments: 1–10 n=154 (69.4%), 11–20 n=47 (21.2%), 21–30 n=12 (5.4%), >31 n=9 (4.1%).<br>Shift pattern: H12 n=37 (16.7%), H24 n=185 (83.3%).<br><br>*Descriptive outcomes*<br>Job satisfaction: Weak n=45 (20.3%), Moderate n=58 (26.1%), Strong n=119 (53.6%).<br>Perceived safety of workplace: Weak n=83 (37.4%), Moderate n=101 (45.5%), Strong n=38 (17.1%).<br><br>Violence exposure:<br>Suffered aggression Yes n=191 (86.0%), No n=31 (14.0%).<br>Type (among exposed): Physical n=66 (29.73%), Verbal n=125 (56.31%).<br>Timing (among exposed): Morning/afternoon n=146 (65.76%), Night n=45 (20.27%).<br>Reported the incident (among exposed): Yes n=127 (57.20%), No n=64 (28.83%).<br><br>Burnout:<br>Emotional exhaustion: Weak n=75 (33.8%), Moderate n=65 (29.3%), Strong n=82 (36.9%).<br>Depersonalization: Weak n=62 (27.9%), Moderate n=44 (19.8%), Strong n=116 (52.3%).<br>Personal accomplishment (low accomplishment = worse): Weak n=69 (31.1%), Moderate n=63 (28.4%), Strong n=90 (40.5%)

				Associations<br>Sex and emotional exhaustion (p=0.034):<br>Female (n=127): Emotional exhaustion Weak n=45 (20.27%), Moderate n=31 (13.96%), Strong n=51 (22.97%).<br>Male (n=95): Emotional exhaustion Weak n=30 (13.51%), Moderate n=34 (15.31%), Strong n=31 (13.96%).<br><br>Shift work and Personal accomplishment (p=0.012):<br>H12 (n=37): Personal accomplishment Weak n=7 (3.15%), Moderate n=8 (3.60%), Strong n=22 (9.91%).<br>H24 (n=185): Personal accomplishment Weak n=62 (27.93%), Moderate n=55 (24.77%), Strong n=68 (30.63%).<br><br>Suffered aggression and Personal accomplishment (p=0.043):<br>No (n=31): Personal accomplishment Weak n=14 (6.31%), Moderate n=7 (3.15%), Strong n=10 (4.50%).<br>Yes (n=191): Personal accomplishment Weak n=55 (24.77%), Moderate n=56 (25.22%), Strong n=80 (36.04%).<br><br>Suffered aggression and depersonalization (p=0.029)<br><br>Job satisfaction:<br>And depersonalization (p=0.004)<br>And personal accomplishment (p=0.002)
Wang et al.,[47]<br><br>2023<br><br>China	Cross-sectional, descriptive study<br><br>N/A<br><br>Gender, age, staff shortage, self-perceived medical errors, job satisfaction, self-efficacy, medical errors, effort-	To determine the prevalence and the associated factors of self-perceived medical errors among Chinese emergency department nurses	Job Satisfaction scale derived from Leiden Quality of Work Questionnaire<br><br>Generalized Self-efficacy Scale (GSE)<br><br>Custom-tailored self-perceived	*Sample characteristics*<br>Gender: female n=15,779 (89.75%), male n=1,803 (10.25%).<br>Age: 29.92 ± 6.75 years.<br>Staff shortage: Yes n=8,834 (50.24%), No n=8,748 (49.76%).<br>Self-perceived medical error (last 3 months): n=4,445/17,582 (25.28%); No error n=13,137 (74.72%).<br>Workplace verbal abuse in past year (times): 0 n=5,351 (30.43%), 1–3 n=7,369 (41.91%), 4–6 n=1,914 (10.89%), ≥7 n=2,948 (16.77%).<br><br>*Descriptive outcomes*<br>Self-perceived physical health: Good n=3,960 (22.52%), Fair n=9,325 (53.04%), Bad n=4,297 (24.44%).

	reward imbalance, self-perceived health, positive and negative affect<br><br>n=17582<br><br>8/8		medical errors questions<br><br>Effort–Reward Imbalance (ERI)<br><br>Patient Health Questionnaire (PHQ-9)<br><br>Positive and Negative Affect Schedule (PANAS)	Effort–Reward Imbalance (ERI): Mean 1.24 ± 0.59; ERI>1 Yes n=10,489 (59.66%), No n=7,093 (40.34%).<br>Over-commitment: 17.59 ± 2.65.<br>PHQ-9: 7.53 ± 4.98; major depressive symptoms n=4,576 (26.03%). Negative affect (PANAS subset): 16.26 ± 3.74.<br>Job satisfaction (Leiden QWL): 13.22 ± 3.34.<br>General self-efficacy (GSE): 26.00 ± 6.03.
Wu et al., [48]<br><br>2024<br><br>China	Cross-sectional, descriptive study<br><br>N/A<br><br>Age, gender, years of experience, patients admitted per day, shifts, injury-related questions, positive and negative affect, perceived health, work-family conflict, effort-reward imbalance<br><br>n=10457<br><br>8/8	To assess the occupational injury of Chinese emergency departments physicians and to identify its associated factors.	Custom-tailored injury-related questions<br><br>Positive and Negative Affect Schedule (PANAS) scale<br><br>Patient Health Questionnaire-9 (PHQ-9)<br><br>Work-Family Conflict Scale<br><br>Effort-Reward Imbalance Scale	*Sample characteristics*<br>Age: 35.0 [31.0–41.0] years (median [IQR]).<br>Gender: Male n=7,632 (73.0%), Female n=2,825 (27.0%).<br>Years of experience: <1 y n=1,448 (13.9%), 1–5 y n=3,965 (37.9%), 6–10 y n=2,458 (23.5%), ≥11 y n=2,586 (24.7%).<br>Patients admitted/day: 15.0 [7.0–30.0].<br>Night shifts/month: None n=494 (4.7%), 1–5 n=1,539 (14.7%), 6–10 n=5,633 (53.9%), ≥11 n=2,791 (26.7%).<br>Perceived physician shortage: Yes n=7,667 (73.3%).<br><br>*Descriptive outcomes*<br>Sleep quality: Very good n=266 (2.5%), Good n=821 (7.8%), Fair n=3,227 (30.9%), Bad n=3,775 (36.1%), Very bad n=2,368 (22.7%);<br>Negative affect (PANAS-NA 5-item) 17.0 [14.0–20.0]<br>Depressive symptoms (PHQ-9 ≥10) n=3,734 (35.7%).<br>Work–family conflict score: 20.0 [16.0–22.0].<br>Effort–reward imbalance (ERR>1) Yes n=8,197 (78.4%).<br>Over-commitment: 18.0 [17.0–19.0].<br><br>Occupational injury:<br>Prevalence: n=8,484/10,457 (81.13%) injured; No injury n=1,973 (18.87%).<br>Causes among those injured (multiple responses):<br>Verbal violence by patients/families 100% (n=8,484/8,484)<br>Injury while moving patients 76.57% (n=6,496)

				Falls/slips/sprains during visits 71.30% (n=6,049)<br>Needle/sharp injuries 42.20% (n=3,580)<br>Emergency car door clamp 30.70% (n=2,605);<br>Injury while moving equipment 29.60% (n=2,511)<br><br>*Comparisons (injury vs no injury)*<br>Age (years, median [IQR]): 36.0 [31–41] vs 35.0 [29–42], p<0.0001<br>Gender (injury prevalence within category): Male n=6,444/7,632 (84.5%) vs Female n=2,040/2,825 (72.2%), p<0.0001.<br>Sleep (injury prevalence): Very good n=158/266 (59.4%), Good 560/821 (68.2%), Fair 2,399/3,227 (74.3%), Bad 3,236/3,775 (85.7%), Very bad 2,131/2,368 (90.0%), p<0.0001.<br>Patients/day 16.0 [8–30] vs 15.0 [5–30], p=0.0003.<br>Night shifts/month (injury prevalence): None 256/494 (51.8%); 1–5 1,082/1,539 (70.3%); 6–10 4,628/5,633 (82.2%); ≥11 2,518/2,791 (90.2%), p<0.0001.<br>Physician shortage (injury prevalence): Yes 6,487/7,667 (84.6%) vs No 1,997/2,790 (71.6%), p<0.0001.<br>PHQ-9 ≥10: Yes 3,277/3,734 (87.8%) vs No 5,207/6,723 (77.5%), p<0.0001.<br>PANAS 17.0 [14–20] vs 15.0 [13–18], p<0.0001<br>Work–family conflict 20.0 [17–23] vs 18.0 [15–20], p<0.0001<br>Over-commitment 18.0 [17–19] vs 18.0 [16–18], p<0.0001.
Xie et al.,[41]<br><br>2023<br><br>China	Cross-sectional, descriptive study<br><br>N/A<br><br>Age, gender, marital status, years of experience, work hours/day, shift, compassion fatigue, occupational stress, workplace violence, job satisfaction<br><br>n=1014	To develop and evaluate a predictive model for compassion fatigue among emergency department nurses	Compassion Fatigue Scale (CF-CN)<br><br>Occupational Stress Scale for Chinese nurses<br><br>Workplace Violence Scale (WVS)	*Sample characteristics*<br>Age: 29.59 ± 4.67 years (range 20–50).<br>Sex: Male n=124 (12.2%), Female n=890 (87.8%).<br>Marital status: Unmarried n=407 (40.1%), Married n=597 (58.9%), Divorced/Separated n=10 (1.0%).<br>Years of experience: 1–5 n=609 (60.1%), 6–10 n=269 (26.5%), >10 n=136 (13.4%). Work hours/day: ≤8 h n=268 (26.4%), >8 h n=746 (73.6%).<br>Shift predominantly worked: Days n=221 (21.8%), Nights n=793 (78.2%).<br><br>*Descriptive outcomes*<br>Job satisfaction: Very satisfied n=38 (3.7%), Satisfied n=283 (27.9%), Normal n=440 (43.4%), Dissatisfied n=211 (20.8%), Very dissatisfied n=42 (4.1%).<br>Sleep hours/day: ≤6 h n=434 (42.8%), >6 h n=580 (57.2%).<br><br>Compassion fatigue:<br>No compassion fatigue n=244 (24.1%).<br>Compassion fatigue (any subscale beyond critical value): n=770/1,014 (75.9%)

	8/8			Compassion fatigue levels: Low n=88 (8.7%), Moderate n=276 (27.2%), High n=406 (40.0%).<br><br>*Comparisons (compassion fatigue vs no compassion fatigue)*<br>Age (years, median [IQR]): 28 [26–32] vs 31 [27–34], p<0.001.<br>Gender: Male n=98/770 (12.7%) vs n=26/244 (10.7%), p=0.389.<br>Marital status: Unmarried n=332 (43.1%) vs 75 (30.7%); Married n=432 (56.1%) vs 165 (67.6%); Divorced/sep. n=6 (0.8%) vs 4 (1.6%); p=0.002.<br>Work hours/day: >8 h n=585 (76.0%) vs 161 (66.0%); ≤8 h n=185 (24.0%) vs 83 (34.0%), p=0.002.<br>Shift predominantly worked: Nights n=629 (81.7%) vs 164 (67.2%); Days n=141 (18.3%) vs 80 (32.8%), p<0.001.<br>Job satisfaction: Very satisfied n=10 (1.3%) vs 28 (11.5%); Satisfied n=176 (22.9%) vs 107 (43.9%); Normal n=344 (44.7%) vs 96 (39.3%); Dissatisfied n=200 (26.0%) vs 11 (4.5%); Very dissatisfied n=40 (5.2%) vs 2 (0.8%), p<0.001.<br>Sleep hours/day: ≤6 h n=367 (47.7%) vs 67 (27.5%); >6 h n=403 (52.3%) vs 177 (72.5%), p<0.001.<br><br>Occupational stress (mean ± SD):<br>Total score: 101.50 ± 14.07 vs 89.05 ± 15.21, p<0.001.<br>Subscales:<br>Nursing workload & time allocation 16.08 ± 2.70 vs 14.23 ± 3.02, p<0.001<br>Nursing professional & work 21.20 ± 3.10 vs 18.77 ± 3.62, p<0.001<br>Patient care 31.62 ± 4.72 vs 28.30 ± 5.21, p<0.001<br>Management & interpersonal 24.55 ± 4.76 vs 20.73 ± 4.94, p<0.001<br>Environment & equipment 8.04 ± 1.91 vs 7.02 ± 1.89, p<0.001.
Yan et al.,[43]<br><br>2021b<br><br>China	Cross-sectional, descriptive study<br><br>N/A<br><br>Age, gender, marital status, workplace violence, self-perceived health, sleep quality,	To explore the prevalence of turnover intentions of emergency physicians	Custom-tailored turnover intention question<br><br>Workplace Violence Scale (WVS)<br><br>Center for Epidemiological	*Sample characteristics*<br>Age: 37.66 ± 8.06 years<br>Male n=10,650 (69.87%), Female n=4,593 (30.13%<br>Marital status: Married/widowed/divorced n=12,691 (83.26%); Unmarried n=2,552 (16.74%).<br>Workplace violence (past year): Yes n=13,699 (89.87%).<br>Self-perceived health: Good n=4,707 (30.88%), Fair n=7,729 (50.71%), Poor n=2,807 (18.42%).<br>Sleep quality: Good n=2,295 (15.06%), Fair n=7,347 (48.20%), Poor n=5,601 (36.74%).<br>Depressive symptoms (CES-D ≥ 20): n=5,425 (35.59%).

	depression, turnover intention<br><br>n=15243<br><br>8/8		Studies Depression scale (CES-D)	*Descriptive outcomes*<br>Turnover intention (Yes): n=7,584/15,243 (49.75%); No n=7,659 (50.25%).<br><br>*Comparisons (turnover intention yes vs turnover intention no)*<br>Gender: Male n=5,443/10,650 (51.11%) vs Female n=2,141/4,593 (46.61%), p<0.01.<br>Age group: ≤31 n=1,993/4,089 (48.74%); >31–37 n=2,309/4,117 (56.08%); >37–43 n=1,714/3,291 (52.08%); >43 n=1,568/3,746 (41.86%), p<0.01.<br>Marital status: Married/widow/divorced n=6,342/12,691 (49.97%) vs Unmarried n=1,242/2,552 (48.67%), p=0.23.<br>Shift work: Yes n=6,918/13,288 (52.06%) vs No n=666/1,955 (34.07%), p<0.01.<br>Workplace violence (past year): Yes n=7,171/13,699 (52.35%) vs No n=413/1,544 (26.75%), p<0.01.<br>Self-perceived health: Good n=1,581/4,707 (33.59%); Fair n=4,045/7,729 (52.34%); Poor n=1,958/2,807 (69.75%), p<0.01.<br>Sleep quality: Good n=735/2,295 (32.03%); Fair n=3,348/7,347 (45.57%); Poor n=3,501/5,601 (62.51%), p<0.01.<br>Depression (CES-D ≥20): Yes n=3,849/5,425 (70.95%) vs No n=3,735/9,818 (38.04%), p<0.01.
Yan et al., [42]<br><br>2021a<br><br>China	Cross-sectional, descriptive study<br><br>N/A<br><br>Age, gender, marital status, job seniority, shift, burnout, depression, workplace violence<br><br>n=15243<br><br>8/8	To investigate difficulties faced by emergency physicians to further develop emergency care, maintain the stability of the population of emergency physicians and determine the influencing factors that have not previously been examined	Maslach Burnout Inventory (MBI)<br><br>Center for Epidemiological Studies Depression scale (CES-D)<br><br>Workplace violence Scale (WVS)	*Sample characteristics*<br>Age (years) 37.66 ± 8.10<br>Gender: Male n=10,650 (69.87%), Female n=4,593 (30.13%).<br>Marital status: Married/widow/divorced n=12,691 (83.26%), Unmarried n=2,552 (16.74%).<br>Job seniority: ≤3 y n=4,921 (32.28%), >3–6 y n=3,114 (20.43%), >6–11 y n=3,424 (22.46%), >11 y n=3,784 (24.82%).<br>Shift work: Yes n=13,288 (87.17%), No n=1,955 (12.83%).<br><br>*Descriptive outcomes*<br>Workplace violence (WVS, past year): Yes n=13,699 (89.87%), No n=1,544 (10.13%).<br>Self-perceived health: Good n=4,707 (30.88%), Fair n=7,729 (50.71%), Poor n=2,807 (18.42%).<br>Sleeping quality: Good n=2,295 (15.06%), Fair n=7,347 (48.20%), Poor n=5,601 (36.74%).<br>Depression (CES-D ≥20): n=5,425 (35.59%)<br><br>Burnout:

Subscale means: emotional exhaustion 25.78 ± 15.94, depersonalization 8.13 ± 7.85, personal accomplishment 26.80 ± 12.53
Categorical prevalence:
High emotional exhaustion: n=7,130/15,243 (46.8%); Moderate n=3,015 (19.8%); Low n=5,098 (33.4%).
High depersonalization: n=3,672 (24.1%); Moderate n=2,970 (19.5%); Low n=8,601 (56.4%).
Low personal accomplishment: n=9,223 (60.5%); Moderate n=2,908 (19.1%); High (good) n=3,112 (20.4%).
High on all three (overall high burnout): n=2,280/15,243 (15.0%)

Burnout group comparisons
Gender:
Male (n=10,650): emotional exhaustion 25.36 ± 16.05, p<0.001; depersonalization 8.46 ± 8.08, p<0.001; personal accomplishment 26.46 ± 12.77, p<0.001.
Female (n=4,593): emotional exhaustion 26.76 ± 15.64; depersonalization 7.38 ± 7.23; personal accomplishment 27.61 ± 11.91.

Age group:
≤31 (n=4,089): emotional exhaustion 24.96 ± 15.76; depersonalization 8.31 ± 7.72; personal accomplishment 25.45 ± 12.37.
>31–37 (n=4,117): emotional exhaustion 28.04 ± 15.69; depersonalization 9.20 ± 8.22; personal accomplishment 26.05 ± 11.86.
>37–43 (n=3,291): emotional exhaustion 27.00 ± 15.85; depersonalization 8.46 ± 7.92; personal accomplishment 27.05 ± 12.23.
>43 (n=3,746): emotional exhaustion 23.13 ± 16.04; depersonalization 6.48 ± 7.21; personal accomplishment 28.90 ± 13.37.

Marital status: Married/widow/divorced (n=12,691) emotional exhaustion 25.88 ± 15.94 / p=0.099; depersonalization 8.06 ± 7.85 / p=0.012; personal accomplishment 26.99 ± 12.56 / p<0.001. Unmarried (n=2,552) emotional exhaustion 25.31 ± 15.93; depersonalization 8.49 ± 7.84; personal accomplishment 25.87 ± 12.34.

Job seniority: ≤3 y (n=4,921) emotional exhaustion 24.41 ± 15.75 / p<0.001; depersonalization 7.87 ± 7.52 / p<0.001; personal accomplishment 26.03 ± 12.45 / p<0.001. >3–6 y (n=3,114) emotional exhaustion 26.76 ± 15.94; depersonalization 8.67 ± 8.16; personal accomplishment 26.20 ± 12.46. >6–11 y (n=3,424) emotional

				exhaustion 27.79 ± 15.80; depersonalization 8.77 ± 8.18; personal accomplishment 27.01 ± 12.19. >11 y (n=3,784) emotional exhaustion 24.94 ± 16.09; depersonalization 7.44 ± 7.62; personal accomplishment 28.12 ± 12.86.<br><br>Shift work: Yes (n=13,288) emotional exhaustion 26.76 ± 15.87 / p<0.001; depersonalization 8.55 ± 7.98 / p<0.001; personal accomplishment 26.50 ± 12.29 / p<0.001. No (n=1,955) emotional exhaustion 19.14 ± 14.81; depersonalization 5.31 ± 6.19; personal accomplishment 28.87 ± 13.87.<br><br>Workplace violence: Yes (n=13,699) emotional exhaustion 27.13 ± 15.65 / p<0.001; depersonalization 8.65 ± 7.92 / p<0.001; personal accomplishment 26.95 ± 12.07 / p<0.001. No (n=1,544) emotional exhaustion 13.79 ± 13.27; depersonalization 3.54 ± 5.27; personal accomplishment 25.50 ± 15.97.<br><br>Self-perceived health: Good (n=4,707) emotional exhaustion 17.60 ± 14.04 / p<0.001; depersonalization 5.54 ± 6.40 / p<0.001; personal accomplishment 27.70 ± 14.25 / p<0.001. Fair (n=7,729) emotional exhaustion 26.85 ± 14.90; depersonalization 8.31 ± 7.53; personal accomplishment 26.53 ± 11.88. Poor (n=2,807) emotional exhaustion 36.55 ± 14.38; depersonalization 11.99 ± 9.14; personal accomplishment 26.05 ± 11.01.<br><br>Sleeping quality: Good (n=2,295) emotional exhaustion 15.39 ± 13.15 / p<0.001; depersonalization 4.66 ± 5.74 / p<0.001; personal accomplishment 28.55 ± 14.62 / p<0.001. Fair (n=7,347) emotional exhaustion 23.46 ± 14.91; depersonalization 7.58 ± 7.31; personal accomplishment 26.48 ± 12.63. Poor (n=5,601) emotional exhaustion 33.09 ± 14.99; depersonalization 10.28 ± 8.61; personal accomplishment 26.51 ± 11.36.<br><br>Depressive symptoms (CES-D):<br><20 (n=9,818) emotional exhaustion 18.54 ± 12.86 / p<0.001; depersonalization 5.02 ± 5.36 / p<0.001; personal accomplishment 27.40 ± 13.64 / p<0.001.<br>≥20 (n=5,425) emotional exhaustion 38.88 ± 12.18; depersonalization 13.76 ± 8.47; personal accomplishment 25.72 ± 10.12.
Zhang et al., [54]<br><br>2025<br><br>China	Cross-sectional, descriptive study<br><br>N/A	To explore the mechanisms through which workplace violence impacts the occupational health	Workplace violence (WPV) questionnaire adapted from two previous ones	*Sample characteristics*<br>Age 32.23 years (SD not reported).<br>Gender: Female n=1,211 (78.6%), Male n=329 (21.4%).<br>Marital status: Unmarried n=560 (36.4%), Married n=980 (63.6%).<br>Years of experience: 1–2 y n=242 (15.7%), 3–10 y n=742 (48.2%), 11–20 y n=421 (27.3%), >20 y n=135 (8.8%).

Age, gender, marital status, years of experience, working hours per week, night shifts per month, workplace violence, sleep disorders, somatic symptoms<br><br>n=1540<br><br>8/8	of emergency and critical care nurses.	Self-administered Sleep Questionnaire (SSQ)<br><br>Somatic Symptom Scale-China (SSS-CN)	Working hours/week: ≤40 h n=577 (37.5%), 41–48 h n=780 (50.6%), 49–58 h n=123 (8.0%), ≥59 h n=60 (3.9%).<br>Night shifts/month: 0 n=181 (11.8%), 1–4 n=239 (15.5%), 5–8 n=659 (42.8%), >8 n=461 (29.9%).<br><br>*Descriptive outcomes*<br>Dyssomnia: Yes n=913 (59.3%), No n=627 (40.7%).<br>Workplace violence (WPV) in the last 12 months: Yes n=1,309 (85.0%), No n=231 (15.0%). Physical violence: Yes n=943 (61.3%). Emotional violence: Yes n=1,299 (84.4%).<br><br>Occupational health (SSS-CN total):<br>Median 40.00 [29.00, 46.00]<br>Groups: >36 in 63.3% (n=975/1,540) and moderate-to-severe symptoms in 53.4% (n=823/1,540).<br><br>*Correlations*<br>WPV and Somatic symptoms: r=0.479 (95% CI 0.438–0.518), p<.01.<br>Dyssomnia and Somatic symptoms: r=0.600 (0.566–0.631), p<.01.<br>WPV and Dyssomnia: r=0.313 (0.266–0.358), p<.01.<br>Physical disorder r=0.464 with WPV; Anxiety r=0.464 with WPV; Depression r=0.456 with WPV; Anxiety & depression r=0.417 with WPV; all p<.01

Note. Authors' own elaboration. N/A: not applicable; ns: non-significant
